# Identification of *OsPK5* involved in rice glycolytic metabolism and GA/ABA balance for improving seed germination via genome-wide association study

**DOI:** 10.1093/jxb/erac071

**Published:** 2022-02-22

**Authors:** Bin Yang, Mingming Chen, Chengfang Zhan, Kexin Liu, Yanhao Cheng, Ting Xie, Peiwen Zhu, Ying He, Peng Zeng, Haijuan Tang, Daisuke Tsugama, Sunlu Chen, Hongsheng Zhang, Jinping Cheng

**Affiliations:** State Key Laboratory of Crop Genetics and Germplasm Enhancement, Jiangsu Collaborative Innovation Center for Modern Crop Production, Jiangsu Province Engineering Research Center of Seed Industry Science and Technology, Cyrus Tang Innovation Center for Seed Industry, Nanjing Agricultural University, Nanjing 210095, China; Guangzhou Key Laboratory for Research and Development of Crop Germplasm resources, Zhongkai University of Agriculture and Engineering, Guangzhou 510225, China; State Key Laboratory of Crop Genetics and Germplasm Enhancement, Jiangsu Collaborative Innovation Center for Modern Crop Production, Jiangsu Province Engineering Research Center of Seed Industry Science and Technology, Cyrus Tang Innovation Center for Seed Industry, Nanjing Agricultural University, Nanjing 210095, China; State Key Laboratory of Crop Genetics and Germplasm Enhancement, Jiangsu Collaborative Innovation Center for Modern Crop Production, Jiangsu Province Engineering Research Center of Seed Industry Science and Technology, Cyrus Tang Innovation Center for Seed Industry, Nanjing Agricultural University, Nanjing 210095, China; State Key Laboratory of Crop Genetics and Germplasm Enhancement, Jiangsu Collaborative Innovation Center for Modern Crop Production, Jiangsu Province Engineering Research Center of Seed Industry Science and Technology, Cyrus Tang Innovation Center for Seed Industry, Nanjing Agricultural University, Nanjing 210095, China; State Key Laboratory of Crop Genetics and Germplasm Enhancement, Jiangsu Collaborative Innovation Center for Modern Crop Production, Jiangsu Province Engineering Research Center of Seed Industry Science and Technology, Cyrus Tang Innovation Center for Seed Industry, Nanjing Agricultural University, Nanjing 210095, China; State Key Laboratory of Crop Genetics and Germplasm Enhancement, Jiangsu Collaborative Innovation Center for Modern Crop Production, Jiangsu Province Engineering Research Center of Seed Industry Science and Technology, Cyrus Tang Innovation Center for Seed Industry, Nanjing Agricultural University, Nanjing 210095, China; State Key Laboratory of Crop Genetics and Germplasm Enhancement, Jiangsu Collaborative Innovation Center for Modern Crop Production, Jiangsu Province Engineering Research Center of Seed Industry Science and Technology, Cyrus Tang Innovation Center for Seed Industry, Nanjing Agricultural University, Nanjing 210095, China; State Key Laboratory of Crop Genetics and Germplasm Enhancement, Jiangsu Collaborative Innovation Center for Modern Crop Production, Jiangsu Province Engineering Research Center of Seed Industry Science and Technology, Cyrus Tang Innovation Center for Seed Industry, Nanjing Agricultural University, Nanjing 210095, China; State Key Laboratory of Crop Genetics and Germplasm Enhancement, Jiangsu Collaborative Innovation Center for Modern Crop Production, Jiangsu Province Engineering Research Center of Seed Industry Science and Technology, Cyrus Tang Innovation Center for Seed Industry, Nanjing Agricultural University, Nanjing 210095, China; State Key Laboratory of Crop Genetics and Germplasm Enhancement, Jiangsu Collaborative Innovation Center for Modern Crop Production, Jiangsu Province Engineering Research Center of Seed Industry Science and Technology, Cyrus Tang Innovation Center for Seed Industry, Nanjing Agricultural University, Nanjing 210095, China; Asian Natural Environmental Science Center (ANESC), The University of Tokyo, 1-1-1 Midori-cho, Nishitokyo-shi, Tokyo 188-0002, Japan; State Key Laboratory of Crop Genetics and Germplasm Enhancement, Jiangsu Collaborative Innovation Center for Modern Crop Production, Jiangsu Province Engineering Research Center of Seed Industry Science and Technology, Cyrus Tang Innovation Center for Seed Industry, Nanjing Agricultural University, Nanjing 210095, China; State Key Laboratory of Crop Genetics and Germplasm Enhancement, Jiangsu Collaborative Innovation Center for Modern Crop Production, Jiangsu Province Engineering Research Center of Seed Industry Science and Technology, Cyrus Tang Innovation Center for Seed Industry, Nanjing Agricultural University, Nanjing 210095, China; State Key Laboratory of Crop Genetics and Germplasm Enhancement, Jiangsu Collaborative Innovation Center for Modern Crop Production, Jiangsu Province Engineering Research Center of Seed Industry Science and Technology, Cyrus Tang Innovation Center for Seed Industry, Nanjing Agricultural University, Nanjing 210095, China; University of Birmingham, UK

**Keywords:** GA/ABA balance, glycolytic metabolism, GWAS, pyruvate kinase, rice, seed germination

## Abstract

Seed germination plays a pivotal role in the plant life cycle, and its precise regulatory mechanisms are not clear. In this study, 19 quantitative trait loci (QTLs) associated with rice seed germination were identified through genome-wide association studies (GWAS) of the following traits in 2016 and 2017: germination rate (GR) at 3, 5, and 7 days after imbibition (DAI) and germination index (GI). Two major stable QTLs, *qSG4* and *qSG11.1*, were found to be associated with GR and GI over 2 continuous years. Furthermore, *OsPK5*, encoding a pyruvate kinase, was shown to be a crucial regulator of seed germination in rice, and might be a causal gene of the key QTL *qSG11.1*, on chromosome 11. Natural variation in *OsPK5* function altered the activity of pyruvate kinase. The disruption of *OsPK5* function resulted in slow germination and seedling growth during seed germination, blocked glycolytic metabolism, caused glucose accumulation, decreased energy levels, and affected the GA/ABA balance. Taken together, our results provide novel insights into the roles of *OsPK5* in seed germination, and facilitate its application in rice breeding to improve seed vigour.

## Teaser

Pyruvate kinase *OsPK5*is identified as the causal gene for major seed germination QTL *qSG11.1*, and functions by affecting glycolytic metabolism and GA/ABA balance in rice.

## Introduction

Seed germination is the initial process in the plant life cycle and plays a crucial role in seedling growth and development (Rajjou *et al.*, 2012). Seeds with high vigour usually germinate fast and uniformly, showing obvious growth advantages and high resistance to abiotic stresses, whereas low-vigour seeds germinate slowly and irregularly, resulting in sprouting weeds and even death (Wang *et al.*, 2010; Rajjou *et al.*, 2012). Rice (*Oryza sativa* L.) is one of the most important food crops in the world. Increasing the speed and uniformity of seed germination is pivotal for the establishment of strong rice seedlings and achieving high yields. Moreover, direct seeding of rice is becoming increasingly popular due to its advantages, in terms of cost and labour savings (Liu *et al.*, 2015; Mahender *et al.*, 2015). Hence, the identification of key genes and elucidation of the molecular mechanisms associated with rice seed germination are important objectives in rice breeding and field production.

Seed germination is defined as starting with the uptake of water by a quiescent dry seed and ending with radicle protrusion (Bewley, 1997). Large amounts of energy and nutrition need to be provided by seed reserves when seeds germinate, to establish seedling growth (Bewley, 1997; Yan *et al.*, 2014). Oxygen uptake increases with water uptake during seed germination, and three respiratory pathways, glycolysis, the pentose phosphate pathway, and the tricarboxylic acid (TCA) cycle, are activated (Rajjou *et al.*, 2012; He and Yang, 2013). Generally, glycolysis produces pyruvate under aerobic conditions. The utilization of pyruvate then occurs within the mitochondria to produce acetyl-CoA, which is important for generating energy via the TCA cycle using sugars, especially glucose molecules (Weitbrecht *et al.*, 2011; He and Yang, 2013). Phytohormones such as abscisic acid (ABA) and gibberellin (GA) play vital roles during seed germination (Penfield and King, 2009; Du *et al.*, 2015; Guo *et al.*, 2013). GA promotes seed germination by activating amylase and protease (He and Yang, 2013; Yang *et al.*, 2019). ABA is induced by glucose and inhibits seed germination (Bewley, 1997; Song *et al.*, 2012). Furthermore, antagonism exists between GA and ABA, and the GA/ABA balance is also reported to be an important factor regulating seed germination (Shu *et al.*, 2016).

To date, hundreds of quantitative trait loci (QTLs) for seed germination have been identified (Fujino *et al.*, 2004; Wang *et al.*, 2010; Li *et al.*, 2011; Jiang *et al.*, 2011; Xie *et al.*, 2014; Cheng *et al.*, 2015; Guo *et al.*, 2019), and some genes related to seed germination have been cloned by map-based cloning in rice (Fujino *et al.*, 2008; Abe *et al.*, 2012; He *et al.*, 2019*b*). *qLTG3-1*, controlling low-temperature germinability, was isolated and shown to be tightly associated with the vacuolation of tissues covering the embryo (Fujino *et al.*, 2008). *qSE3/OsHAK21*, encoding a K^+^ transporter gene, was shown to promote rice seed germination and seedling establishment through regulating ABA and reactive oxygen species (ROS) content in germinating seeds under salinity stress (He *et al.*, 2019*b*). The GA biosynthesis-related gene *OsGA20ox1* is considered a candidate gene for a major QTL controlling seedling vigour (Abe *et al.*, 2012). Furthermore, *OsFbx352*, encoding an F-box domain protein, plays a regulatory role in the regulation of the glucose-induced suppression of seed germination by targeting ABA metabolism (Song *et al.*, 2012). *OsIPMS1* encodes isopropylmalate synthase, and provides more energy for rapid seed germination and the vigorous growth of seedlings (He *et al.*, 2019*a*). *OsIAGLU* encodes indole-3-acetate beta-glucosyltransferase, which has been reported to regulate seed vigour by mediating the crosstalk between auxin (IAA) and ABA in rice (He *et al.*, 2020). These results imply that seed germination is influenced by hormones and energy metabolism.

A genome-wide association study (GWAS) based on linkage disequilibrium (LD) is a comprehensive and powerful approach for evaluating single-nucleotide polymorphisms (SNPs) directly associated with complex traits (Huang *et al.*, 2010; Zhao *et al.*, 2011). Given the development of high-throughput sequencing technology, GWAS has been widely applied to detect novel loci for complex rice traits, such as flowering time (Zhao *et al.*, 2011; Yano *et al.*, 2016), stress tolerance (Lv *et al.*, 2016; Li *et al.*, 2017), seed vigour (Cheng *et al.*, 2015; Wang *et al.*, 2018), and yield (Huang *et al.*, 2010; Zhao *et al.*, 2011). Based on 413 diverse rice accessions collected from 82 countries, Rice Diversity Panel 1 (RDP1), comprising 700 000 SNP variants, was constructed for high-resolution genotyping for GWAS (Zhao *et al.*, 2011; McCouch *et al.*, 2016). RDP1 has been employed for the identification of associations with loci for multiple traits, such as starch structure (Butardo *et al.*, 2017), low-temperature germination (Wang *et al.*, 2018), panicle exsertion and the uppermost internodes (Zhan *et al.*, 2019). The 3000 Rice Genomes Project was carried out based on 3000 rice accessions with global representation of genetic and functional diversity (Li *et al.*, 2014). The estimated effect of nucleotide polymorphisms was also determined to rapidly identify candidate genes associated with traits via GWAS (Yano *et al.*, 2016). Thus, GWAS might be an efficient method for identifying key genes regulating rice seed germination.

In this study, we conducted a GWAS of the germination rate (GR) at 3, 5, and 7 days after imbibition (DAI), and the germination index (GI) across the 2 years using a portion of RDP1. Through further comparisons and analyses, we identified the key loci and found that the pyruvate kinase (PK) gene *OsPK5*, located within *qSG11.1*, was significantly associated with seed germination. Therefore, the physiological and molecular changes in the *ospk5* mutants were analysed to elucidate the *OsPK5* regulatory mechanism during seed germination in rice. Our results could contribute to understanding the genetic and molecular mechanisms of seed germination for the breeding of rice varieties with high-vigour seeds in the future.

## Materials and methods

### Plant materials and growth

The seeds of 263 *O. sativa* accessions from RDP1 identified by Zhao *et al.* (2011) at the McCouch laboratory, Cornell University, USA used in this study were obtained from Dr Jian Hua, Cornell University, USA. These accessions were divided into 55 *tropical japonica* (*TRJ*), 63 *temperate japonica* (*TEJ*), 7 *aromatic* (*ARO*), 49 *aus* (*AUS*), 56 *indica* (*IND*), and 33 *ADMIX* accessions (Supplementary Table S1). Three *ospk5* mutants (*ospk5-1*, *ospk5-2* and *ospk5-3*) were generated in the *japonica* background (*Oryza sativa* L. ‘Nipponbare’) using the CRISPR/Cas9 system (Xing *et al.*, 2014).

All seedlings were planted in the field at Jiangpu Experimental Station of Nanjing Agricultural University (Jiangsu Province, China) in mid-June 2016 and 2017. Field management was performed following local standard methods (Cheng *et al.*, 2015). All seeds were harvested in their maturity stage, dried at 30 °C for 3 d for desiccation and stored at 25 °C for one month, to break seed dormancy for the germination assay.

### Evaluation of seed germination

Fifty healthy seeds of each accession or mutant line were placed on Petri dishes (9 cm diameter) with double sheets of filter paper and 10 ml distilled water. All Petri dishes were incubated in a constant temperature (25 °C) incubator with a 12 h/12 h light-dark cycle for 7 d. The number of germinated seeds was observed daily. Germination was defined as the emergence of the radicle through the hull by ≥2 mm. Seedlings were considered to have developed when the root length reached the seed length and the shoot length reached half of the seed length. Seed germination was measured based on the GR at 3, 5, and 7 DAI and GI. GR was expressed as GR = (total germinated seeds/50) × 100%, and GI was calculated as *GI = ∑(Gt/t)*, where *Gt* is the number of germinated seeds on day *t* (Wang *et al.*, 2010). Three replicates of each accession or mutant line were included in this study.

### Genome-wide association study (GWAS)

A high-density array of 700 000 SNPs described by McCouch *et al.* (2016) was used in this study. SNPs with a minor allele frequency (MAF) ≤5% and a missing ratio ≥25% were filtered (Yano *et al.*, 2016) using TASSEL 5.2.40 software (Bradbury *et al.*, 2007). The final set included 403 950 SNPs, which were subsequently employed for GWAS. Linear mixed-model association studies were implemented using the EMMAX in Linux (Kang *et al.*, 2010). The significance threshold for all trait associations was set to *P* ≤1.0e-5, as indicated by a red horizontal line in the Manhattan plot at –log_10_*P* ≥5, according to Crowell *et al.* (2016). The GWAS results for seed germination were visualized in Manhattan and quantile-quantile plots using the R package qqman. Clear peak signals with significant SNP clusters (at least three SNPs) were considered to represent one associated locus between any two significant SNPs within a 200 kb interval (Lv *et al.*, 2016). The most significant (i.e. the highest –log_10_*P*) SNP in a cluster was considered to be a lead SNP. The loci with the same lead SNP IDs or common physical positions were integrated into a single QTL associated with seed germination.

### Candidate gene analysis of *qSG11.1*

To identify the causal genes of *qSG11.1*, candidate genes were predicted within a 200 kb genomic region (±100 kb of each significant SNP) based on the ‘Nipponbare’ reference genome (Lv *et al.*, 2016) according to the MSU Rice Genome Annotation Project Release 7 database (http://rice.plantbiology.msu.edu). The expression patterns of candidate genes were analysed in the imbibed seeds of rice using the Expression Atlas database (https://www.ebi.ac.uk/gxa/home). According to Yano *et al.* (2016), all the significant SNPs in the candidate region of *qSG11.1* based on their location in the reference genome were categorized into five functional groups using the RiceVarMap v2.0 database (http://ricevarmap.ncpgr.cn/; Zhao *et al.*, 2015). Group I consisted of significant SNPs (–log_10_*P* ≥5) causing amino acid substitutions; Group II comprised significant SNPs located in promoter regions and 5ʹ non-coding sequences; Group III contained significant SNPs that were located within a coding region but were not predicted to change an amino acid, or were located within an intron or a 3ʹ non-coding sequence; Group IV included significant SNPs that were located in intergenic regions; and Group V contained SNPs that were not significantly associated with seed germination.

### Linkage disequilibrium (LD) and haplotype analyses

LD was estimated based on squared allele frequency correlations (*r*^2^) for pairs of SNPs. LD plots were visualized using the LD heatmap R package. For the key *qSG11.1* locus associated with seed germination, the haplotypes were classified based on the linkage relationships in a functional range covering the entire *OsPK5* gene and its promoter. The haplotypes comprising at least 10 investigated varieties were selected for comparative analysis (Dong *et al.*, 2016).

### Phylogenetic analysis

The amino acid sequences of the OsPK5 homologs were aligned using ClustalX (http://www.clustal.org). A phylogenetic tree was generated based on the Neighbor-joining method (1000 replications of bootstrap tests) using MEGA 5.2 software (http://www.megasoftware.net/; Tamura *et al.*, 2011). The resulting tree was visualized using the online tool EvolView (https://www.evolgenius.info/evolview/) (Zhang *et al.*, 2012).

### Pyruvate kinase (PK) activity assay

To validate the causal gene of *qSG11.1*, ten extreme accessions (five from Hap1 with high GRs and GI, and five from Hap2 with low GRs and GI; Supplementary Table S2) as well as the *ospk5* mutants and WT (‘Nipponbare’) were selected to determine PK activity. PK activity was measured using commercial assay kits according to the manufacturer’s instructions (Comin Biotechnology Co. Ltd. Suzhou, Jiangsu, China). PK activity was determined via the coupling reaction of lactate dehydrogenase, which catalyses the transformation of pyruvate and nicotinamide adenine dinucleotide (NADH) to produce lactic acid and NAD^+^. The oxidation of NADH was monitored according to the absorbance value at a 340 nm wavelength by using a SpectraMax M3 Microplate Reader (Molecular Devices, San Jose, NM, USA). PK activity was calculated based on the fresh weight (FW) using the following formula: PK activity (nmol min^-1^ g^-1^) = 3216 ×A_340_/FW. Three biological replicates were conducted.

### Generation and identification of transgenic plants

The CRISPR/Cas9 plasmid was constructed based on a previously reported strategy (Xing *et al.*, 2014). Two target sites of *OsPK5* were designed with CRISPR-PLANT (http://omap.org/crispr). The target fragment of 964 bp was amplified from the pCBC-MT1T2 vector using OsPK5-BsF/BsR and OsPK5-F0/R0 primers, cloned into the pBUE411 vector, and then validated using the TaU3-FD2, TaU3-RD, and OsU3-FD3 primers. The pCBC-MT1T2 and pBUE411 vectors were obtained from Qijun Chen’s laboratory (College of Biological Sciences, China Agricultural University, China). Transgenic rice plants were generated using the *Agrobacterium*-mediated co-cultivation method in the background of ‘Nipponbare’. The genomic DNA of mutant seedlings was extracted using the cetyltrimethylammonium bromide (CTAB) method (Murray and Thompson, 1980). The DNA fragments surrounding the two target sites of *OsPK5* were amplified from genomic DNA using the primers OsPK5-CRISPR-F1/R1 and OsPK5-CRISPR-F2/R2, and then directly sequenced to identify the mutants. T_2_ plants of the CRISPR/Cas9 mutants were used for phenotype analysis. All the primers are listed in Supplementary Table S3.

### RNA extraction and qRT-PCR

To determine whether two haplotypes affect *OsPK5* expression, seeds of ten extreme accessions after 24 h of imbibition (Supplementary Table S2) were selected to compare the transcript levels of *OsPK5*. Dry seeds, germinated seeds, leaf, sheath, panicle, stem, stem node and roots were used to explore the expression pattern of *OsPK5* in *japonica* ‘Nipponbare’ (WT) plants. Total RNA from each plant tissue was isolated using the TransZol Plant reagent kit (TransGen Biotech Co. Ltd., Beijing, China) following the manufacturer’s protocol. First-strand cDNA synthesis was performed with ~500 ng of total RNA using the HiScript^®^ II Reverse Transcriptase system (Vazyme Biotech Co. Ltd. Nanjing, China). qRT–PCR experiments were performed using the Roche LightCycler 480 Real-time System (Roche, Swiss Confederation). The rice *ACTIN* gene (*OsActin*, *LOC_Os03g50885*) and *18S RIBOSOMAL RNA* were used as internal references to normalize gene expression using the comparative CT method described by Livak and Schmittgen (2001). The gene-specific primers used for qRT–PCR are listed in Supplementary Table S3. Three biological replicates were performed.

### Seed metabolite analysis

For the analysis of seed metabolites involved in glycolysis, seeds of the *ospk5* mutants and WT (‘Nipponbare’) were collected at 24 and 48 hours after imbibition (HAI). Phosphoenolpyruvate (PEP), pyruvate (Pyr), glucose, sucrose, fructose, soluble sugar, α-amylase activity and ATP were extracted and measured using commercial assay kits according to the manufacturer’s instructions (MDBio, Inc., Qingdao, Shangdong, China). Three biological replicates were performed.

### Hormone quantification

Approximately 1 g of *ospk5* mutant and WT seeds collected at 48 HAI was rapidly frozen in liquid nitrogen and homogenized into a powder. The hormones ABA, GA_1_, and GA_4_ were extracted from each sample using the method described by He *et al.* (2019*b*). The hormone concentrations were then quantified using a high-performance liquid chromatography (HPLC) system (Waters Instruments Inc., USA). Three biological replications were performed.

### Data analysis

Phenotype data and variance were calculated with Excel 2017 software. The significant differences were tested using Student’s *t*-test or Fisher’s least significant difference (LSD) test at the 5% and 1% levels of probability. Broad-sense heritability was calculated using the method described by Zhan *et al.* (2019). The correlation coefficients for multiple traits related to seed germination were calculated using the R package Corrplot.

## Results

### Phenotypic variation in seed germination

**Fig. 1.**
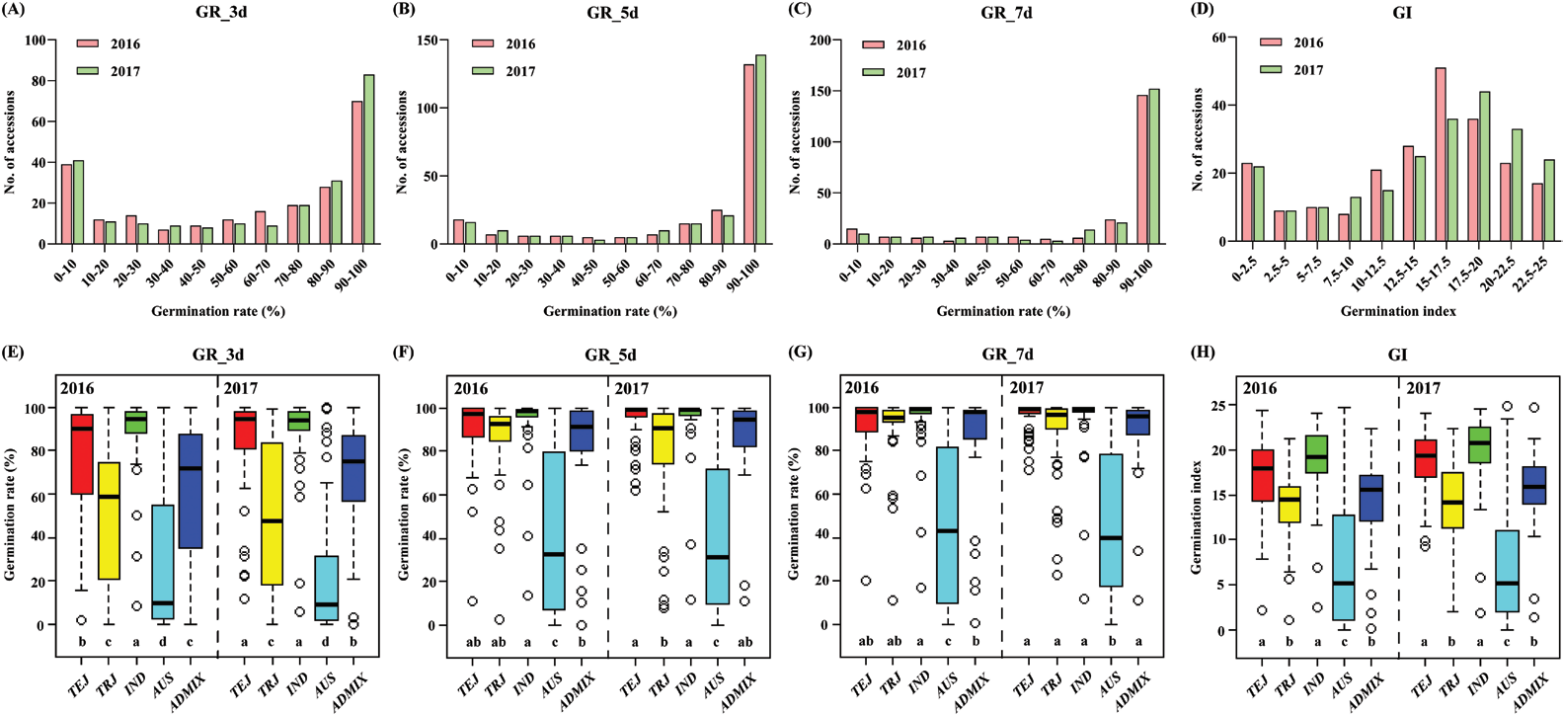
Phenotypic variation and distribution of GRs at 3, 5, and 7 DAI and GIs in RDP1. (A-D) Histogram showing the distributions of GR_3d (A), GR_5d (B), GR_7d (C), and GIs (D) in 2016 and 2017. (E-H) Boxplots of GR_3d (E), GR_5d (F), GR_7d (G), and GI (H) in the *TEJ*, *TRJ*, *IND*, *AUS* and *ADMIX* sub-groups in 2016 and 2017. The *P* values were calculated using Student’s *t*-test. Different lowercase letters indicate significant differences at *P*<0.05. Differences between sub-groups were analysed by Fisher’s least significant difference (LSD) test. *ARO* was excluded from the analysis due to the low sample size.

To characterize the variation in seed germination in rice, a set of 263 natural accessions were selected for the evaluation of their germination traits, including their GRs at 3, 5, and 7 DAI and GI, in 2016 and 2017 (Supplementary Table S1). Phenotypic statistics showed that GRs at 3, 5, and 7 DAI ranged from 0–100% in both years and that GI ranged from 0–24.75 in 2016 and from 0–24.83 in 2017 (Fig. 1; Supplementary Table S4). In 2016 and 2017, the average GRs at 3 DAI were 59.97% and 62.43%, with coefficients of variation (CVs) of 0.6 and 0.59, respectively. The average GRs at 5 DAI in 2016 and 2017 were 77.79% and 78.57%, with CVs of 0.4 and 0.39, respectively; and the average GRs at 7 DAI were 80.71% and 82.34%, with CVs of 0.38 and 0.34, respectively. Overall, GR showed lower average values and larger variations at 3 DAI than at 5 and 7 DAI. The average GIs were 14.1 and 14.91, with CVs of 0.47 and 0.46 in 2016 and 2017, respectively (Fig. 1; Supplementary Table S4). The GRs at 3, 5, and 7 DAI showed skewed distributions with higher values (Fig. 1A-C), and the GI showed approximatively normal distributions in the 2 years (Fig. 1D). These data revealed large phenotypic variation in GR and GI traits in this population.

By comparing the values of various sub-groups, we found that the GR at 3 DAI in *TEJ* and *IND* were significantly higher (*P*<0.05) than those in *TRJ*, *AUS* and *ADMIX* (Fig. 1E), whereas the GR at 5 and 7 DAI and GI in *AUS* were significantly lower (*P*<0.05) than those in *TEJ, TRJ*, *IND* and *ADMIX* in both 2016 and 2017 [Fig. 1F-H; *ARO* not shown due to the small number (7) of accessions]. Larger phenotypic variations in the GR at 3, 5, and 7 DAI and GI were observed in *AUS* than in the other sub-groups (Fig. 1E-H). These results indicate that the variations in seed germination are significantly affected by sub-group structures.

The correlation coefficients among the seed germination traits between the 2 years were calculated. Positive correlations were observed among the GR at 3, 5, and 7 DAI and GI in both years (Supplementary Fig. S1). Two-way analysis of variance (ANOVA) revealed that both the GR and GI showed broad-sense heritability higher than 90%, and the G × E interactions were all significant (*P*<0.001; Supplementary Table S4), suggesting that seed germination is regulated by both genetic and environmental factors.

### Loci associated with seed germination according to GWAS

**Fig. 2.**
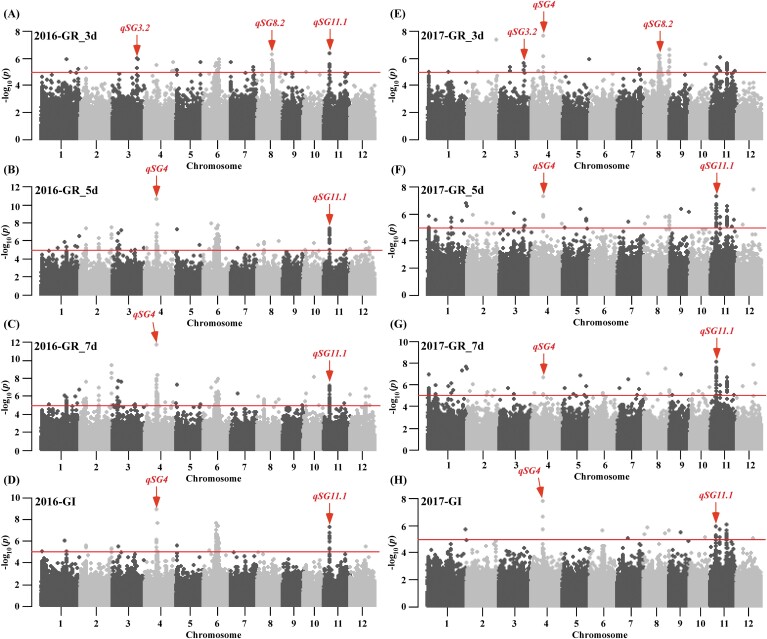
Genome-wide association analysis of GRs at 3, 5, and 7 DAI and GI in 2016 and 2017. Manhattan plots of GR_3d, GR_5d, and GR_7d and GIs in 2016 (A-D) and 2017 (E-H). The horizontal red lines indicate a statistical significance threshold of *P*<1 × 10^-5^, according to Crowell *et al*. (2016). The red arrows indicate that the four QTLs were detected in both years.

According to the method used by Crowell *et al.* (2016), GWAS of the GR at 3, 5, and 7 DAI and GI was conducted in the 2 years using the Efficient Mixed-Model Association eXpedited (EMMAX) model with –log_10_*P* ≥5 as the significance threshold (Fig. 2; Supplementary Fig. S2). In total, 47 loci were shown to be associated with GR and GI in this study (Supplementary Table S5, Fig. S3). In 2016, four, eight, and 12 loci were associated with the GR at 3, 5, and 7 DAI, respectively, and four loci were associated with GI. In 2017, six, five, and five loci were associated with the GR at 3, 5, and 7 DAI, respectively, and three loci were associated with GI. Based on the lead SNP ID or position, these loci could be integrated into 19 QTLs (Supplementary Table S5, Fig. S3), 11 of which were located in regions of selective sweep or improvement during rice domestication and breeding (Supplementary Table S6). Comparison with previously reported QTLs showed that 14 QTLs co-localized with reported QTLs, while the remaining 5 QTLs (*qSG2.3*, *qSG3.1*, *qSG4*, *qSG8.2* and *qSG11.2*) might be novel QTLs identified in this study (Supplementary Table S5, Fig. S3). Four QTLs (*qSG3.2*, *qSG4*, *qSG8.2* and *qSG11.1*) were detected in both 2016 and 2017 (Fig. 2; Supplementary Table S5), and five QTLs (*qSG4*, *qSG6.1*, *qSG6.2*, *qSG11.1*, and *qSG11.3*) were associated with both GR and GI (Supplementary Table S5, Fig. S3). Interestingly, *qSG4* and *qSG11.1* were simultaneously associated with GR and GI over 2 continuous years, revealing that they might be the key loci for rice seed germination. *qSG11.1*, on chromosome 11, also co-localized with *qSD-11,* as reported by Guo *et al.* (2004). Hence, the function of the key *qSG11.1* locus was further characterized in this study.

### *OsPK5* is the causal gene of *qSG11.1* for seed germination

**Fig. 3.**
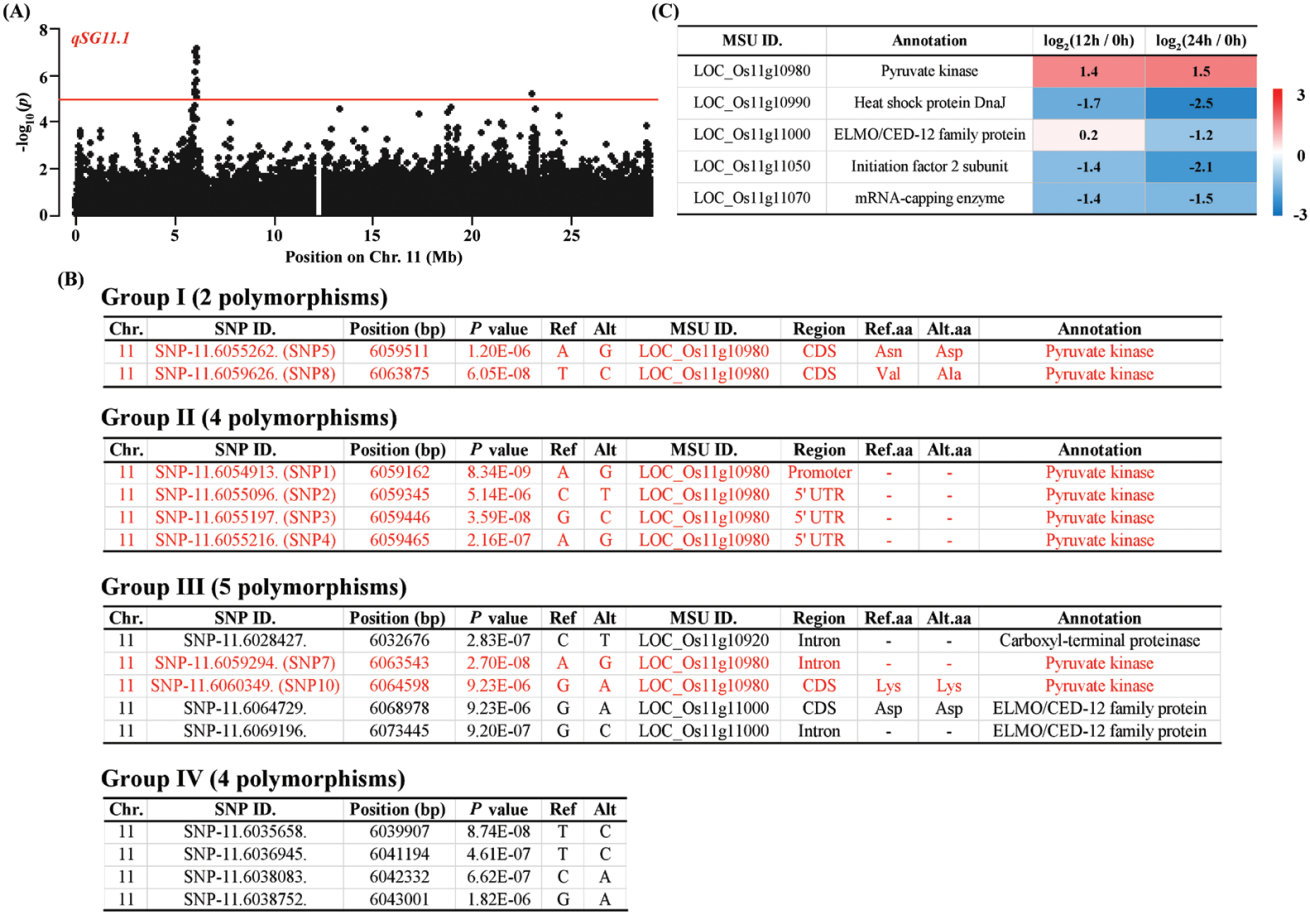
Identification of candidate genes for *qSG11.1*. (A) Local Manhattan plot for chromosome 11. (B) Significant SNP analysis in the candidate region of *qSG11.1*. All 15 significant SNPs (–log_10_*P*≥5) in the candidate regions of *qSG11.1* were classified into five groups (Groups I–V, V is not shown) as described by Yano *et al.* (2016). Among these SNPs, eight significant SNPs (SNP1-5, SNP7-8, and SNP10) shown in red were located in the *OsPK5* gene and its promoter. (C) Heat map showing differentially expressed genes with at least a 2-fold change in the candidate region of *qSG11.1* in seeds at 12 and 24 h after imbibition relative to dry seeds (0 h).

To identify the causal gene of *qSG11.1* controlling seed germination (Fig. 3A), we analysed the candidate genes within a 200 kb region of *qSG11.1* with the ‘Nipponbare’ reference genome (http://rice.plantbiology.msu.edu). A total of 24 candidate genes were identified after the removal of genes encoding transposons and retrotransposons (Supplementary Table S7). To assess these candidate genes, all the significant SNPs in the region of *qSG11.1* were classified into five groups (I-V) according to their functions (Yano *et al.* 2016). The results showed that *LOC_Os11g10980* was located within Group I, and that its two significant SNPs (–log_10_*P* ≥5; SNP5 and SNP8) were non-synonymous variants in the coding DNA sequence (CDS) region (Fig. 3B; Supplementary Table S8). In addition, *LOC_Os11g10980* had four significant SNPs within Group II (–log_10_*P* ≥5; SNP1, SNP2, SNP3 and SNP4) and two significant SNPs within Group III (–log_10_*P* ≥5; SNP7 and SNP10; Fig. 3B; Supplementary Table S8). We also analysed the expression patterns of 24 candidate genes based on transcription profiling from the Expression Atlas database (https://www.ebi.ac.uk/gxa/home). The up-regulated expression of *LOC_Os11g10980* and down-regulated expression of *LOC_Os11g10990*, *LOC_Os11g1100*, *LOC_Os11g11050* and *LOC_Os11g11070* showed at least a 2-fold change in 12 h- and 24 h-imbibed seeds relative to dry seeds (Fig. 3C). Taken together, the results indicated that *LOC_Os11g10980* might be the causal candidate gene of *qSG11.1*.

**Fig. 4.**
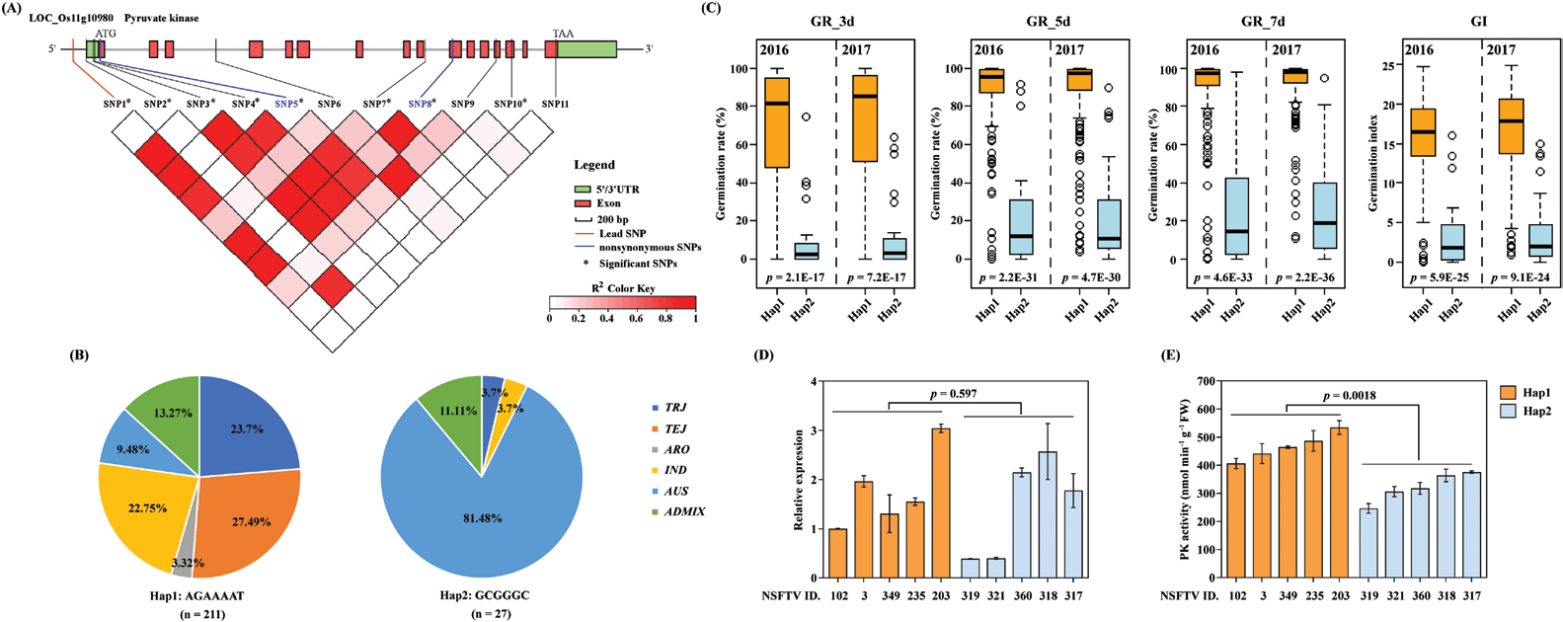
LD and haplotype analysis of *OsPK5*. (A) Gene model of *OsPK5* and linkage disequilibrium (LD) analysis. In the gene model, the filled green and red boxes represent untranslated regions (UTRs) and exons, respectively; the red and blue lines indicate the lead SNP and non-synonymous variants, respectively; stars represent significant SNPs. As a measure of LD, the colour intensity of the box corresponds to the size of the *r*^2^ value, as shown in the legend. (B) Distribution frequencies of the two *OsPK5* haplotypes in different sub-groups. (C) Boxplots of GR_3d, GR_5d, and GR_7d based on the haplotypes of *OsPK5* in 2016 and 2017. Box plots represent the interquartile range (IQR), the whiskers represent 1.5 times the IQR, the thick line in the middle of each box represents the median, and the circles represent outlier points. (D) Comparison of transcript levels between the two haplotypes (Hap1 and Hap2) of *OsPK5*. The relative expression of *OsPK5* was calibrated against those of the *OsActin* gene and quantified by qRT–PCR. (E) Comparison of pyruvate kinase activity between the two haplotypes (Hap1 and Hap2) of *OsPK5*. X-axis labels show the NSFTV ID of rice accession. Differences between haplotypes were analysed by Student’s *t*-test (*P* value indicated).

*LOC_Os11g10980*, encoding a PK, was designated as *OsPK5*. Homology analysis showed that *OsPK5* was homologous to *OsPK1* (Supplementary Figs S4, S5). In a previous study, the germination of the *ospk1* mutant was shown to be delayed by nearly 2 d compared with that of the WT (Zhang *et al.*, 2012*b*). The results of calculating the LD with all SNPs of *OsPK5* showed that five significant SNPs (–log_10_*P* ≥5), SNP3, SNP4, SNP5, SNP7, and SNP8, located in *OsPK5*, were linked with the lead SNP (SNP1; *r*^2^> 0.8; Fig. 4A; Supplementary Table S8). Therefore, SNP1 and its five significantly linked SNPs (–log_10_*P* ≥5) were used for the haplotype analysis of *OsPK5*. Two haplotypes were detected (Fig. 4B). The accessions of Hap1 (AGAAAT type) were distributed in the *TEJ*, *TRJ*, *IND*, *ADMIX*, *AUS* and *ARO* sub-groups, with frequencies of 27.49%, 23.7%, 22.75%, 13.27%, 9.48% and 3.32%, respectively (Fig. 4B). However, most accessions of Hap2 (GCGGGC type) were distributed in *AUS*, with a frequency of 81.48% (Fig. 4B). By comparison, the average GRs at 3, 5, and 7 DAI and GI of Hap1 were significantly higher (*P*<0.01) than those of Hap2 in both years (Fig. 4C), suggesting that Hap1 was a potentially superior haplotype of *OsPK5* for improving rice seed germination.

To investigate the effect of these SNPs on *OsPK5* expression, we selected five Hap1 accessions with extremely high GR and GI, and five from Hap2 with extremely low GR and GI (Supplementary Table S2), to measure the expression of *OsPK5* during seed germination by quantitative reverse transcription PCR (qRT–PCR). However, there were no significant differences (*P*=0.597 and *P*=0.185) in *OsPK5* expression between Hap1 and Hap2 (Fig. 4D; Supplementary Fig. S6). In the high-SNP-density dataset of RDP1, there are two non-synonymous variants (SNP5 and SNP8) in the coding region of *OsPK5*. To confirm this, we re-sequenced the coding region of *OsPK5* from six accessions with two different haplotypes. Besides SNP5 and SNP8, a new variant was identified in the coding region of *OsPK5*, but did not cause amino acid changes (Supplementary Table S9). By predicting the protein structure (http://www.sbg.bio.ic.ac.uk/phyre2/), two non-synonymous variants in the coding region of *OsPK5* resulted in a difference in the three-dimensional structure of the proteins (Supplementary Fig. S7), which might affect PK enzyme activity. Therefore, PK activity was further measured in the above ten extreme rice accessions during seed germination. The results showed that the PK activity of the Hap1 accessions was significantly higher (*P*=0.0018) than that of the Hap2 accessions (Fig. 4E). These data suggest that the natural variation in *OsPK5* function altered PK activity and might be closely associated with seed germination.

### Expression pattern of *OsPK5*

**Fig. 5.**
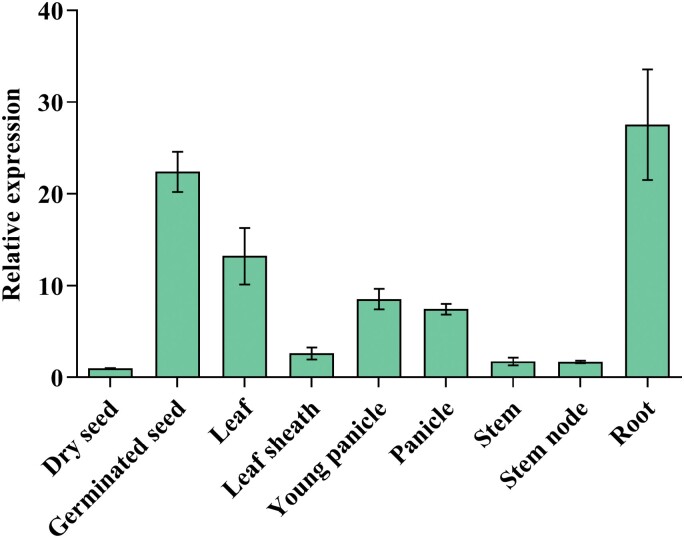
Expression pattern of *OsPK5* in different tissues. The relative expression was calibrated against the expression of the *OsActin* gene and quantified by qRT–PCR.

To expand our understanding of the physiological function of *OsPK5*, the expression patterns of *OsPK5* were further analysed via qRT–PCR. The detection of *OsPK5* expression in different tissues revealed that *OsPK5* was highly expressed in roots, germinated seeds, leaves, and panicles, and relatively low in dry seeds, leaf sheaths, stems and stem nodes (Fig. 5; Supplementary Fig. S8). These results indicate that *OsPK5* may play essential roles in rice seed germination.

### OsPK5 regulates seed germination

**Fig. 6.**
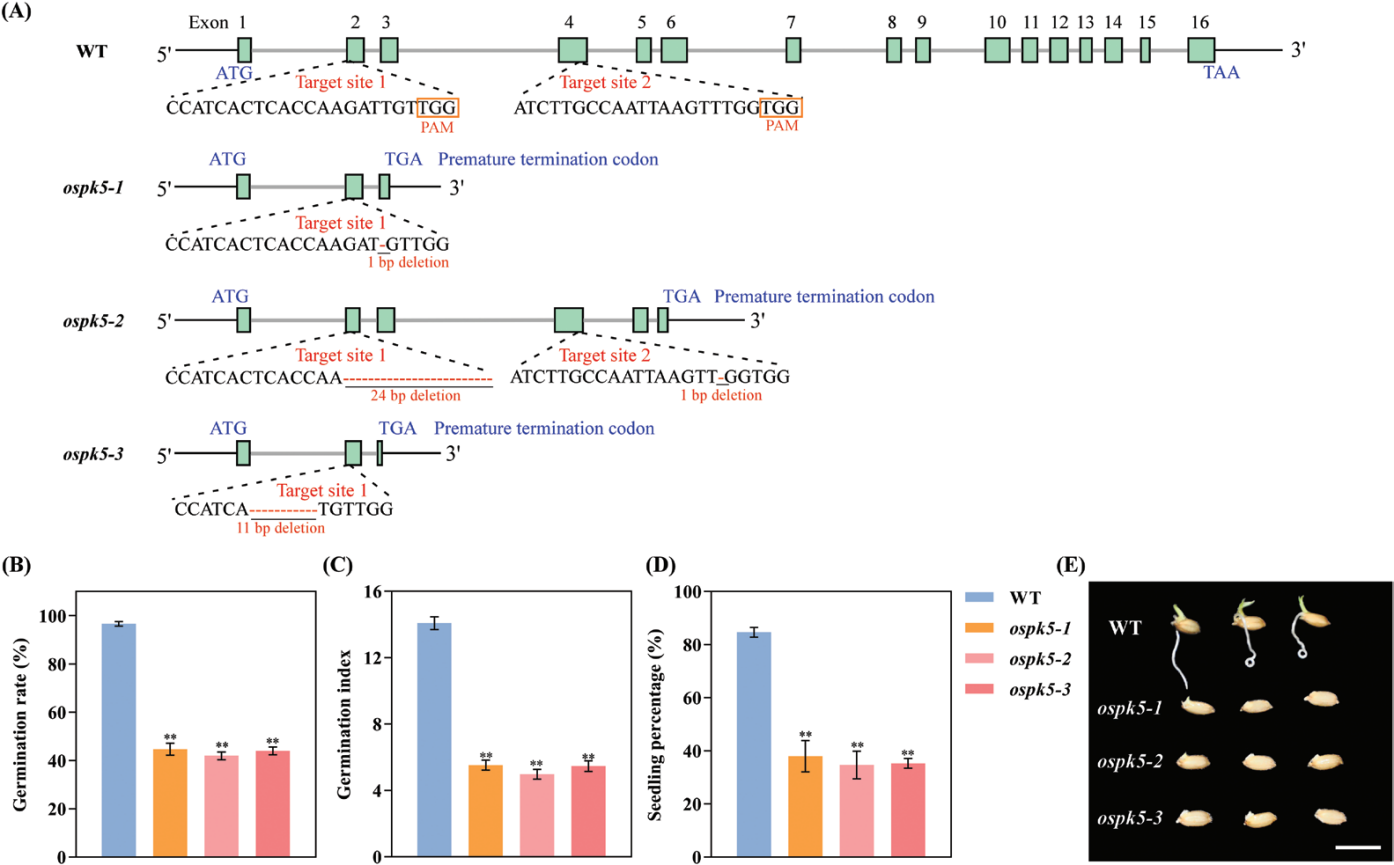
Seed germination is regulated by *OsPK5*. (A) Schematic representation of the generation of *OsPK5* mutant lines via the CRISPR/Cas9 system. Blue rectangles represent the exons of *OsPK5*. (B-D) Comparison of germination rates (B), germination indices (C), and seedling percentages (D), between the mutant lines (*ospk5-1*, *ospk5-2* and *ospk5-3*) and WT at 7 d after imbibition. (E) Seed germination phenotype of mutant lines and WT after 4 d of imbibition. Scale bars =1 cm. Differences between the mutant lines and WT were analysed by Student’s *t*-test. ** indicates a significant difference between mutant lines and WT at *P*<0.01.

To characterize the function of *OsPK5* in seed germination, the CRISPR/Cas9 system was employed to generate mutants of *OsPK5* in *japonica* ‘Nipponbare’, and three *ospk5* mutants were obtained, *ospk5-1*, *ospk5-2* and *ospk5-3*. *ospk5-1* contained a ‘T’ deletion in the second exon of *OsPK5*; *ospk5-2* contained a 24 bp (-GATTGTTGGGACCCTTGGGCCCAA-) deletion in the second exon and a ‘T’ deletion in the fourth exon of *OsPK5*; and *ospk5-3* contained an 11 bp (-CTCACCAAGAT-) deletion in the second exon of *OsPK5* (Fig. 6A). As predicted based on these nucleotide sequences, the amino acid sequence of OsPK5 contained only 75, 170, and 55 amino acids in *ospk5-1*, *ospk5-2* and *ospk5-3*, respectively, suggesting that these mutations cause premature termination of the translation of *OsPK5* (Supplementary Fig. S9). This indicated that the three *ospk5* mutants might lack a functional OsPK5.

The progeny of the three homozygous mutants were selected to evaluate their traits related to seed germination, GR, GI and seedling percentage. The results showed that the GRs and GIs of *ospk5-1*, *ospk5-2* and *ospk5-3* were significantly reduced (*P*<0.01) compared with those of WT during seed germination (Fig. 6B, C, E; Supplementary Fig. S10A). There was also a significant reduction (*P*<0.01) in seedling percentage in *ospk5-1*, *ospk5-2* and *ospk5-3* relative to WT (Fig. 6D; Supplementary Fig. S10B). These results demonstrated that the *ospk5* mutations resulted in slow germination and seedling growth during seed germination.

### Disruption of *OsPK5* blocks glycolytic metabolism during seed germination

**Fig. 7.**
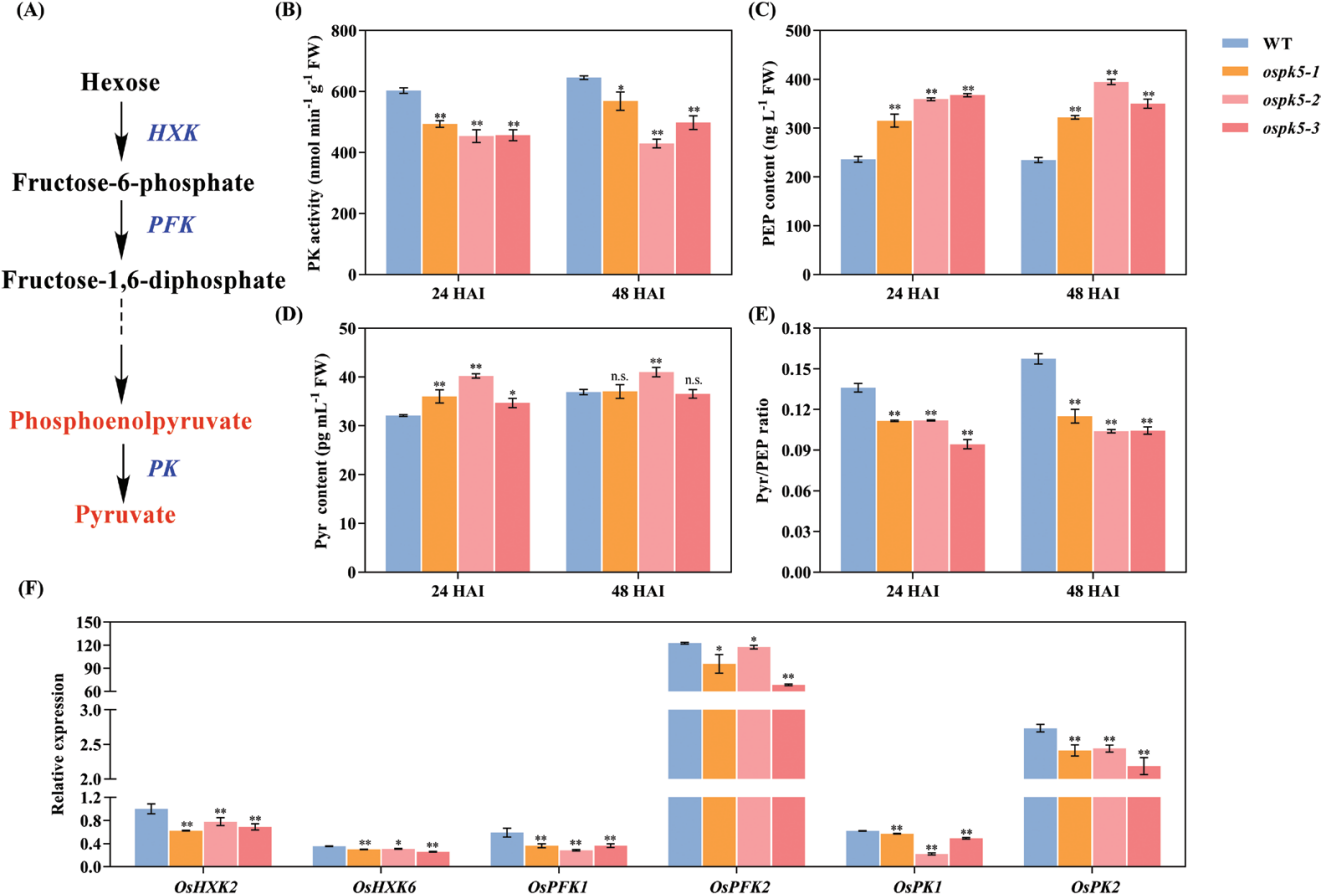
Glycolytic metabolite analysis of *ospk5* mutants and WT during seed imbibition. (A) The glycolysis pathway in plants. Hexokinase (HXK) catalyses the conversion of hexose to fructose 6-phosphate. Phosphofructokinase (PFK) converts fructose 6-phosphate to fructose 1,6-bisphosphate. Pyruvate kinase (PK) catalyses the irreversible final step of glycolysis from phosphoenolpyruvate to pyruvate. (B-E) Comparison of PK activity (B), PEP content (C), Pyr content (D), and Pyr/PEP ratio (E) at different imbibition stages (24 and 48 hours after imbibition, HAI) between mutant lines (*ospk5-1*, *ospk5-2* and *ospk5-3*) and WT. (F) Expression analyses of genes related to the glycolysis pathway at 24 HAI in WT and mutant lines. The relative expression was calibrated against the expression of the *OsActin* gene and quantified by qRT–PCR. Differences between the mutant lines and WT were analysed by Student’s *t*-test. * and ** indicate significant differences between mutant lines and WT at *P*<0.05 and *P*<0.01, respectively. n.s. indicates not statistically significant.

PK is a key regulatory enzyme of glycolytic metabolism and catalyses the final step of glycolysis, irreversibly converting phosphoenolpyruvate (PEP) to pyruvate (Pyr; Fig. 7A). Thus, we measured PK activity and the PEP and Pyr contents of the *ospk5* mutants and WT during seed germination. The results showed that the amount of PK activity was significantly decreased (*P*<0.05) in the *ospk5* mutants compared with those in WT at 24 and 48 HAI (Fig. 7B), whereas the PEP content was significantly increased (*P*<0.01) in the *ospk5* mutants relative to WT at 24 and 48 HAI (Fig. 7C). The Pyr content was also significantly increased (*P*<0.05) in the *ospk5* mutants relative to WT at 24 HAI (Fig. 7D). The ratio of Pyr/PEP was significantly decreased (*P*<0.01) in the *ospk5* mutants relative to WT at 24 and 48 HAI (Fig. 7E). These data reveal that the disruption of *OsPK5* blocks glycolytic metabolism in *ospk5* mutants during seed germination.

qRT–PCR was further used to determine the transcript levels of *OsHXK2* and *OsHXK6* (encoding hexokinase), *OsPFK1* and *OsPFK2* (encoding phosphofructokinase), and *OsPK1* and *OsPK2*; these are six key genes in the regulation of glycolysis (Fig. 7A). The results showed that the transcript levels of *OsHXK2*, *OsHXK6*, *OsPFK1*, *OsPFK2*, *OsPK1*, and *OsPK2* were significantly lower (*P*<0.05) in the *ospk5* mutants than in WT at 24 HAI (Fig. 7F; Supplementary Fig. S11). These data suggest that the disruption of *OsPK5* restricted the transcriptional expression of genes related to glycolytic metabolism in *ospk5* mutants during seed germination.

### Disruption of *OsPK5* enhances glucose accumulation and decreases ATP during seed germination

**Fig. 8.**
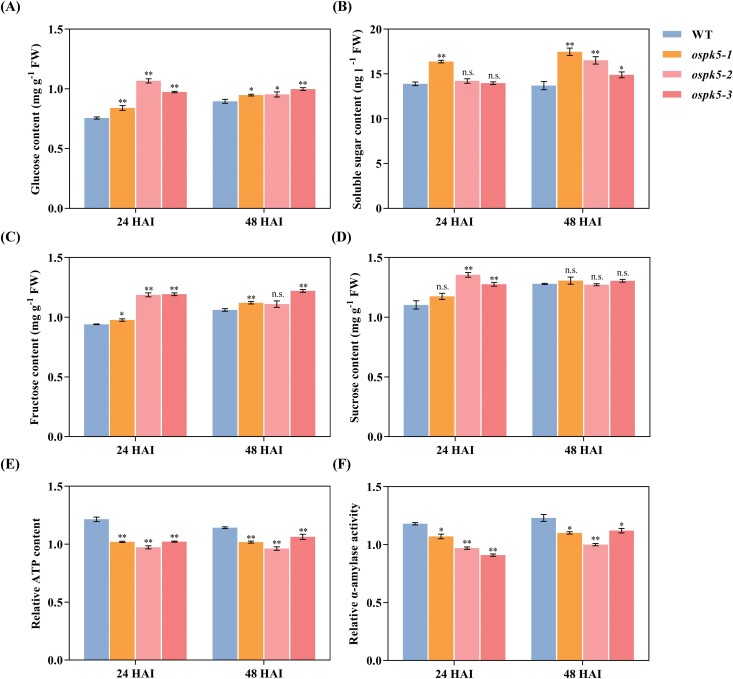
Sugar content analysis of *ospk5* mutants and WT during seed imbibition. (A-F) Comparison of glucose content (A), soluble sugar content (B), fructose content (C), sucrose content (D), relative ATP content (E), and relative α-amylase activity (F) at different imbibition stages (24 and 48 hours after imbibition, HAI) between mutant lines (*ospk5-1*, *ospk5-2* and *ospk5-3*) and WT. The relative content or activity is based on the dried seeds as a reference. Differences between the mutant lines and WT were analysed by Student’s *t*-test. * and ** indicate significant differences between mutant lines and WT at *P*<0.05 and *P*<0.01, respectively. n.s. indicates not statistically significant.

To further examine the regulation of glycolytic metabolism by *OsPK5*, the carbohydrate precursors and energy production of glycolysis were assayed during seed germination. The glucose content of the *ospk5* mutants was significantly higher (*P*<0.05) than that of the WT at 24 and 48 HAI (Fig. 8A). The soluble sugar content at 48 HAI and the fructose content at 24 HAI in the *ospk5* mutant, and the sucrose content at 24 HAI in the *ospk5-2* and *ospk5-3* mutants, were also significantly higher (*P*<0.05) than those of WT plants (Fig. 8B-D). The relative content of adenosine triphosphate (ATP) and α-amylase activity were significantly lower (*P*<0.05) in the *ospk5* mutants than in WT at 24 and 48 HAI (Fig. 8E, F). These data indicate that glucose accumulation and energy decline during seed germination occurred in the *ospk5* mutants.

### OsPK5 affects GA/ABA balance during seed germination

**Fig. 9.**
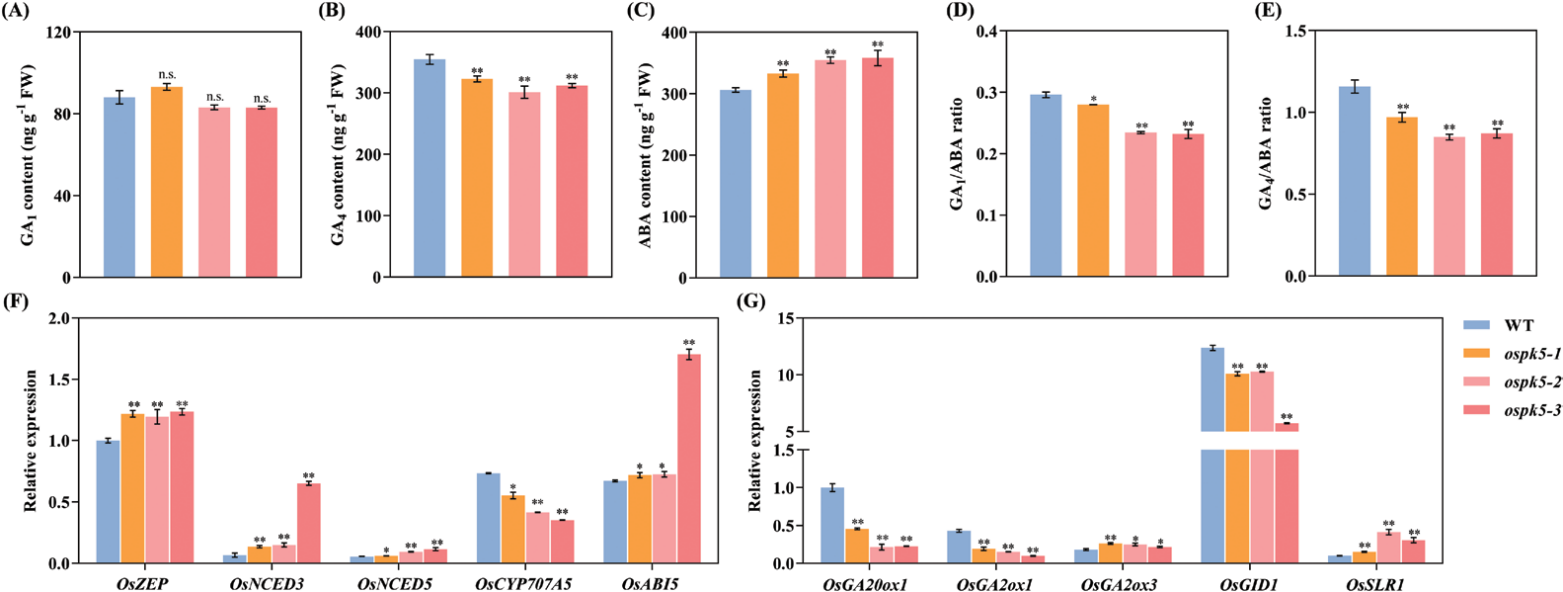
Hormone content analysis of *ospk5* mutants and WT during seed imbibition. (A-C) Comparison of the GA_1_ (A), GA_4_ (B) and ABA (C) hormone concentrations at 48 HAI between mutant lines (*ospk5-1*, *ospk5-2* and *ospk5-3*) and WT. (D, E) Comparison of GA_1_/ABA (D) and GA_4_/ABA (E) ratios at 48 HAI between mutant lines and WT. (F, G) Expression analyses of genes related to the ABA (F) and GA (G) metabolism pathways at 24 HAI. ABA biosynthesis genes: *OsZEP*, *OsNCED3* and *OsNCED5*; ABA catabolism gene: *OsCYP707A5*; ABA signalling gene: *OsABI5*; GA biosynthesis genes: *OsGA20ox1*; GA catabolism genes: *OsGA2ox1* and *OsGA2ox3*; GA signalling genes: *OsGID1* and *OsSLR1*. The relative expression was calibrated against the expression of the *OsActin* gene and quantified by qRT–PCR. HAI represents hours after imbibition. Differences between the mutant lines and WT were analysed by Student’s *t*-test. * and ** indicate significant differences between mutant lines and WT at *P*<0.05 and *P*<0.01, respectively. n.s. indicates not statistically significant.

Excess glucose can affect the metabolic pathways of ABA and GA, which are involved in controlling seed germination (Zhu *et al.*, 2009; Hsu *et al.*, 2014). In this study, significant accumulation (*P*<0.05) of glucose was observed in the *ospk5* mutants (Fig. 8A). We presumed that the delayed germination of the *ospk5* mutants might be associated with changes in ABA, GA and the GA/ABA balance. Hence, ABA and GA content in imbibed seeds of the *ospk5* mutants and WT at 48 HAI was quantified. The data showed that the GA_1_ content was not significantly different (*P>*0.05) between the *ospk5* mutants and the WT (Fig. 9A), but there was a significant increase (*P*<0.01) in the ABA content and a significant reduction (*P*<0.01) in the GA_4_ content in the seeds of the *ospk5* mutants relative to WT (Fig. 9B, C). Additionally, the ratios of GA_1_/ABA and GA_4_/ABA were both significantly lower (*P*<0.05) in the seeds of the *ospk5* mutants than in WT (Fig. 9D, E).

We further monitored the transcription patterns of the key genes involved in the ABA and GA pathways, including genes related to ABA biosynthesis (*Zeaxanthin epoxidase*, *OsZEP*; *9-cis-epoxycaretonoid dioxygenase*, *OsNCED3* and *OsNCED5*), ABA catabolism (*ABA 8’-hydroxylase*, *OsCYP707A5*), ABA signalling (*ABA insensitive 5*, *OsABI5*), GA biosynthesis (*GA 20-oxidase*, *OsGA20ox1*), GA catabolism (*GA 2-oxidase*, *OsGA2ox1* and *OsGA2ox3*) and GA signalling (*GA-insensitive dwarf 1*, *OsGID1*; *Slender rice 1*, *OsSLR1*). The results revealed significantly higher (*P*<0.05) transcript levels of *OsZEP*, *OsNCED3*, *OsNCED5,* and *OsABI5* and significantly lower (*P*<0.05) transcript levels of *OsCYP707A5* in the *ospk5* mutants than in WT at 48 HAI (Fig. 9F; Supplementary Fig. S12A). Moreover, there were significantly higher (*P*<0.05) transcript levels of *OsGA2ox3* and *OsSLR1* and lower (*P*<0.01) transcript levels of *OsGA20ox1*, *OsGA2ox1* and *OsGID1* in the *ospk5* mutants than in WT at 48 HAI (Fig. 9G; Supplementary Fig. S12B). These observations suggest that *OsPK5* participates in the regulation of the GA/ABA balance during seed germination in rice.

## Discussion

### Phenotype analysis and GWAS of seed germination traits

Seed germination is an important trait in rice seedling growth. In this study, using 263 accessions included in RDP1 (Zhao *et al.*, 2011), we investigated seed germination based on the following traits across the 2 years: of GR at 3, 5, and 7 DAI, and GI (Fig. 1; Supplementary Table S1). Large phenotypic variations in the GR and GI traits were found in this population (Supplementary Table S4), which was consistent with findings from a previous study related to low temperature germination (Wang *et al.*, 2018). The evaluated seed germination traits showed high heritability and large variation (Supplementary Table S4), in agreement with other recent reports in rice (Ghosal *et al.*, 2020; Thapa *et al.*, 2020). The mean GR and GI values were lowest in *AUS* among the rice sub-groups in both years (Fig. 1), similar to the results regarding low-temperature germination reported by Wang *et al.* (2018) and Yang *et al.* (2020). In addition, a few accessions of *AUS* showed very low GRs below 10%, even at 7 DAI (Supplementary Table S1), possibly due to the strong seed dormancy of these accessions of *AUS*, similar to wild rice (*Oryza rufipogon*).

In the last two decades, hundreds of QTLs for seed germination in rice have been identified (Fujino *et al.*, 2004; Wang *et al.*, 2010; Jiang *et al.*, 2011; Li *et al.*, 2011; Xie *et al.*, 2014; Cheng *et al.*, 2015; Guo *et al.*, 2019). In the 2 years of our study, a total of 19 QTLs for GRs at 3, 5, and 7 DAI, and GI, were identified by GWAS (Fig. 2; Supplementary Table S5; Fig. S3), suggesting their favourable alleles may be useful for improving germination ability in rice. By comparison, 11 QTLs overlapped with previously reported rice domestication and improvement regions (Supplementary Table S6; Molina *et al.*, 2011; Huang *et al.*, 2012; Xie *et al.*, 2015; Cui *et al*., 2019), indicating that these loci were likely selected during the process of rice domestication and breeding. Besides, 14 QTLs identified in this study overlapped or co-localized with previously reported QTLs (Supplementary Table S5), suggesting the reliability of GWAS for identifying QTLs for rice seed germination. Five novel QTLs (*qSG2.3*, *qSG3.1*, *qSG4*, *qSG8.2,* and *qSG11.2*) were identified in this study (Supplementary Table S5), which demonstrates that more genes in RDP1 contribute to variation in seed germination than previously known.

### *OsPK5*, as the causal gene of *qSG11.1*, contributes to seed germination

The key *qSG11.1* locus of interest in this study contained enormously significant SNPs and overlapped with a previously reported QTL, *qSD-11* (Guo *et al.*, 2004). We therefore focused on *qSG11.1* to explore its causal genes in rice seed germination. Within the *qSG11.1* locus, we found that SNP1 was located in the promoter region of *OsPK5*, and two significant SNPs (SNP5 and SNP8) in the CDS region were non-synonymous variants (Fig. 3B). Moreover, the expression of *OsPK5* was significantly up-regulated in 12 h- and 24 h-imbibed seeds compared with those in dry seeds, based on the transcriptome data (Fig. 3C). Therefore, we speculated that *OsPK5* encoding PK was the causal gene of *qSG11.1* regulating seed germination. Furthermore, two kinds of *OsPK5* haplotypes were identified. The GR and GI of Hap1 were significantly higher than those of Hap2 (Fig. 4C), suggesting that Hap1 shows a high seed germination capacity. A significant difference in PK activity was observed between Hap1, with high-GR and GI accessions, and Hap2, with low-GR and GI accessions (Fig. 4E), confirming that natural variation in *OsPK5* function might have a significant influence on the seed germination of distinct rice accessions.

At least 14 and 10 putative PK isozymes are found in the Arabidopsis and rice genomes, respectively. In Arabidopsis, *pkp1* and *pkp2* mutants and double mutants thereof show reduced seed oil content and lower germination rates (Andre and Benning, 2007; Baud *et al.*, 2007). In rice, *OsPK1* is involved in plant morphological development, and *OsPK1* mutation results in dwarfism, a reduced seed setting rate, altered monosaccharide metabolism, and delayed seed germination (Zhang *et al*., 2012*a*, *b*). *OsPK2/PKpα1* affects starch compound granule formation, starch synthesis, grain filling, and the germination rates of seeds after 1 year of storage (Cai *et al*., 2018*a*, *b*). The OsPK3-OsPK1/OsPK4 complexes, which are two mitochondria-associated PK complexes, are involved in the regulation of grain filling via the stage-specific fine-tuning of sucrose translocation (Hu *et al.*, 2020). In this study, the disruption of *OsPK5* resulted in slow germination and seedling growth during seed germination (Fig. 6; Supplementary Fig. S10). The function of *OsPK5* is similar to those of *OsPK1* and *OsPK2* in rice (Zhang *et al.*, 2012*b*; Cai *et al.*, 2018*a*) and *PKp1* and *PKp2* in Arabidopsis (Andre and Benning, 2007; Baud *et al.*, 2007). The quantification of grain traits, including grain length, grain width, grain thickness and grain weight, revealed no significant difference between the *ospk5* mutants and WT (Supplementary Fig. S13A-D). By further comparing other agronomic traits between the *ospk5* mutants and WT, it was found that there were no significant differences in the heading date, plant height, seed setting rate, and number of effective tillers per plant (Supplementary Fig. S13E-H). It reveals that *OsPK5* was different from the findings for other *OsPK* genes, and might play specific roles in seed germination without adverse effects on other agronomic traits. Hence, we will generate the overexpressed lines of *OsPK5* to confirm it in the future.

### OsPK5 regulates seed germination through glycolytic metabolism

Previous studies have shown that glycolysis is crucial in rice seed germination (He and Yang, 2013; He *et al.*, 2019*a*) and Arabidopsis (Andre and Benning, 2007; Rajjou *et al.*, 2008). As the final step in the glycolytic pathway, PK catalyses the transformation of PEP and adenosine diphosphate (ADP) into Pyr and ATP (Grodzinski *et al.*, 1999; Andre and Benning, 2007). We found that PK activity and Pyr/PEP ratio was significantly decreased in the *ospk5* mutants relative to WT during seed germination (Fig. 7B, E). This is consistent with the results observed following the mutation of rice *OsPK1* or Arabidopsis *PKP1* (Andre *et al.*, 2007; Zhang *et al.*, 2012*b*). These findings imply that PK activity positively correlates with the Pyr/PEP ratio (Andre *et al.*, 2007) and that the disruption of *OsPK5* resulted in PK deficiency by restricting the conversion of PEP into Pyr during seed germination. The significant increases of Pyr in the *ospk5* mutants (Fig. 7D) were similar to the trend observed in the *pkp1* mutants (Andre *et al.*, 2007), possibly because Pyr cannot be utilized due to a lack of ATP. Moreover, the expression of four key genes regulating glycolysis (*OsHXK2*, *OsHXK6*, *OsPFK1* and *OsPFK2*) were reduced in the *ospk5* mutants relative to WT during seed germination (Fig. 7F; Supplementary Fig. S11), suggesting that the accumulation of PEP could contribute to inhibiting the activity of hexokinase (HXK) and phosphofructokinase (PFK) in seeds (Plaxton and Podestá, 2006). A reduction in *OsPK1* and *OsPK2* expression was observed (Fig. 7F; Supplementary Fig. S11), suggesting that the *OsPK5* mutation might also partly affect the function of other *OsPKs* during seed germination. Therefore, these results suggest that the disruption of *OsPK5* alters glycolytic metabolism during seed germination.

Previous studies showed that *ospkpa1* grains were characterized by a marked decrease in the sugar content during seed development in rice (Cai *et al.*, 2018*b*). Soluble sugar accumulation is also greatly decreased in developing *ospk3* seeds in rice (Hu *et al.*, 2020). Conversely, the accumulation of sugar is observed in developing and germinating seeds of Arabidopsis *pkp1* (Andre *et al.*, 2007; Andre and Benning, 2007). In this study, a significantly increased glucose content was observed in the *ospk5* mutants during seed germination (Fig. 8A), compared with that in WT. The accumulation of soluble sugar, sucrose, and fructose was also observed in *ospk5* (Fig. 8B-D). These results are in agreement with the strong correlation between sugar content in germinating seeds and the delayed germination of Arabidopsis *pkp1* plants (Andre and Benning, 2007), suggesting that the loss of function of *OsPK5* may make it defective in catalysing the transformation of endogenous sugar into fuel for germination. Indeed, impaired sugar metabolism could be inferred from the fact that the *ospk5* mutants showed a lower content of ATP than WT plants during seed germination (Fig. 8E). A reduction in α-amylase activity was observed in the *ospk5* mutants relative to WT during seed germination (Fig. 8F), which was presumably due to repression by increasing sugar content derived from starch hydrolysis by α-amylases in germinating seeds (Ranjhan *et al.*, 1992; Poschet *et al.*, 2011). Therefore, it is possible that the accumulation of sugar in seed tissue resulted from a reduction in the glycolytic flux in the *ospk5* mutants, and was responsible for subsequent germination defects.

### OsPK5 improves seed germination by increasing the GA/ABA ratio

ABA and GA play essential roles in seed germination, and it seems likely that glucose and other sugars exert their suppressive effects through the biosynthesis, catabolism or signalling pathways of these hormones (Yuan and Wysocka-Diller, 2006; Xing *et al.*, 2009; Rajjou *et al.*, 2012; Song *et al.*, 2012). In this study, endogenous glucose accumulated at high concentration during seed germination in the *ospk5* mutants (Fig. 8A). Given the significant accumulation of glucose in the *ospk5* mutants, we assumed that GA, ABA, and the GA/ABA balance might be altered by excess glucose to delay seed germination. Accordingly, the results showed (Fig. 9B-E) that *OsPK5* mutation increased ABA concentration but decreased the concentration of GA_4_, which resulted in a reduction in the GA/ABA ratio during seed germination. In this study, the ABA biosynthesis-related genes *OsZEP*, *OsNCED3*, and *OsNCED5* were significantly up-regulated in the *ospk5* mutants, and the ABA catabolic gene *CYP707A5* was significantly down-regulated in the *ospk5* mutants (Fig. 9F). These findings indicated that the glucose-mediated delay in seed germination or post-germination growth was directly related to the induction of several ABA biosynthesis genes and the repression of an ABA catabolic gene (Cheng *et al.*, 2002; Gibson, 2005; Xing *et al.*, 2009; Zhu *et al.*, 2011). In addition, we found that the disruption of *OsPK5* considerably enhanced ABA signalling by increasing *OsABI5* transcription, and reduced GA signalling by decreasing *OsGID1* transcription and increasing *OsSLR1* transcription (Fig. 9F, G), which was consistent with the findings that glucose delays germination by activating the ABA signalling pathway and inhibiting the GA signalling pathway (Yuan and Wysocka-Diller, 2006). These results indicate that the *OsPK5* mutation affects the GA/ABA balance, which may be mediated by endogenous glucose.

In summary, 19 QTLs associated with seed germination were identified by GWAS of GR and GI in the 2 year study, among which two key QTLs were identified: *qSG4* and *qSG11.1*, on chromosomes 4 and 11, respectively. Furthermore, *OsPK5*, encoding a PK, was found to be a crucial regulator of seed germination in rice and can be considered a causal gene of the key *qSG11.1* locus. The disruption of *OsPK5* blocked glycolytic metabolism, caused glucose accumulation, decreased energy levels, and affected the GA/ABA balance during rice seed germination. In contrast to other *OsPK* genes reported previously (Zhang *et al.*, 2012*b*; Cai *et al.*, 2018*a*), *OsPK5* has a specific function in seed germination without adverse effects on other agronomic traits, which is particularly beneficial in rice breeding. Taken together, our results provide novel insights into the roles of *OsPK5* in seed germination, and may have potential application in rice breeding to improve seed vigour.

## Supplementary data

The following supplementary data are available at *JXB* online.

Fig. S1. Pairwise correlation analysis of GRs at 3, 5, and 7 DAI and GIs between the 2 years. The colour and size of the circles represent the degree of correlation between pairwise comparisons.

Fig. S2. Quantile-quantile plots of GRs at 3, 5, and 7 DAI and GIs. Quantile-quantile plots showing the observed associations between SNPs and seed germination compared with expected associations. The red straight line in the quantile-quantile plot represents the expected null distribution of *P*-value and blue dots represent the observed distribution of *P*-values.

Fig. S3. Distribution of 19 QTLs on 12 chromosomes according to physical position.

Fig. S4. Phylogenetic tree analyses of pyruvate kinases in rice and Arabidopsis.

Fig. S5. Comparison of amino acid sequences between OsPK5 and OsPK1 in rice.

Fig. S6. *OsPK5* expression in the two haplotypes during seed germination as determined by qRT–PCR with *18S RIBOSOMAL RNA* as the reference gene.

Fig. S7. Protein structure prediction of two different haplotypes of OsPK5 using Phyre2.

Fig. S8. *OsPK5* expression in different tissues as determined by qRT–PCR with *18S RIBOSOMAL RNA* as the reference gene.

Fig. S9. Comparison of amino acid sequences between *ospk5* mutants and WT rice. Identical and conserved amino acids are shaded in blue and red, respectively.

Fig. S10. Dynamic seed germination phenotype between *ospk5* mutants and WT rice.

Fig. S11. Expression of genes involved in glycolysis pathway as determined by qRT–PCR with *18S RIBOSOMAL RNA* as the reference gene.

Fig. S12. Expression of genes involved in ABA and GA metabolism pathways as determined by qRT–PCR with *18S RIBOSOMAL RNA* as the reference gene.

Fig. S13. Comparison of agronomic traits between *ospk5* mutants and WT in rice.

Table S1. Information on the 263 rice accessions used for association analysis.

Table S2. Phenotypic data of the extreme accessions containing Hap1 or Hap2 of *OsPK5*.

Table S3. The primer pairs used in this study.

Table S4. Descriptive statistics of GRs at 3, 5, and 7 DAI and GIs over 2 years.

Table S5. Details of the significant QTLs for GRs at 3, 5, and 7 DAI and GIs for 2 years.

Table S6. GWAS signals overlapped with the selective sweep regions or improvement regions during rice domestication and breeding.

Table S7. The list of 947 candidate genes in the 19 significant QTLs.

Table S8. The polymorphic sites of *OsPK5* in the high-SNP-density dataset of RDP1.

Table S9. Verification of the polymorphic sites in the coding region of *OsPK5* between Hap1 and Hap2 by resequencing.

erac071_suppl_Supplementary_Figures_S1-S13_Tables_S1-S9

## Data Availability

All data supporting the findings of this study are available within the paper and within its supplementary data published online.
