# Supplementary material for: Identification of OsPK5 involved in rice glycolytic metabolism and GA/ABA balance for improving seed germination via genome-wide association study
Source: J Exp Bot. 2022 Feb 22;73(11):3446–61. doi: 10.1093/jxb/erac071 (PMC9162179; doi:10.1093/jxb/erac071)
Supplement: erac071_suppl_Supplementary_Figures_S1-S13_Tables_S1-S9 [file erac071_suppl_supplementary_figures_s1-s13_tables_s1-s9.pdf]

Supplemental Figure S1-13

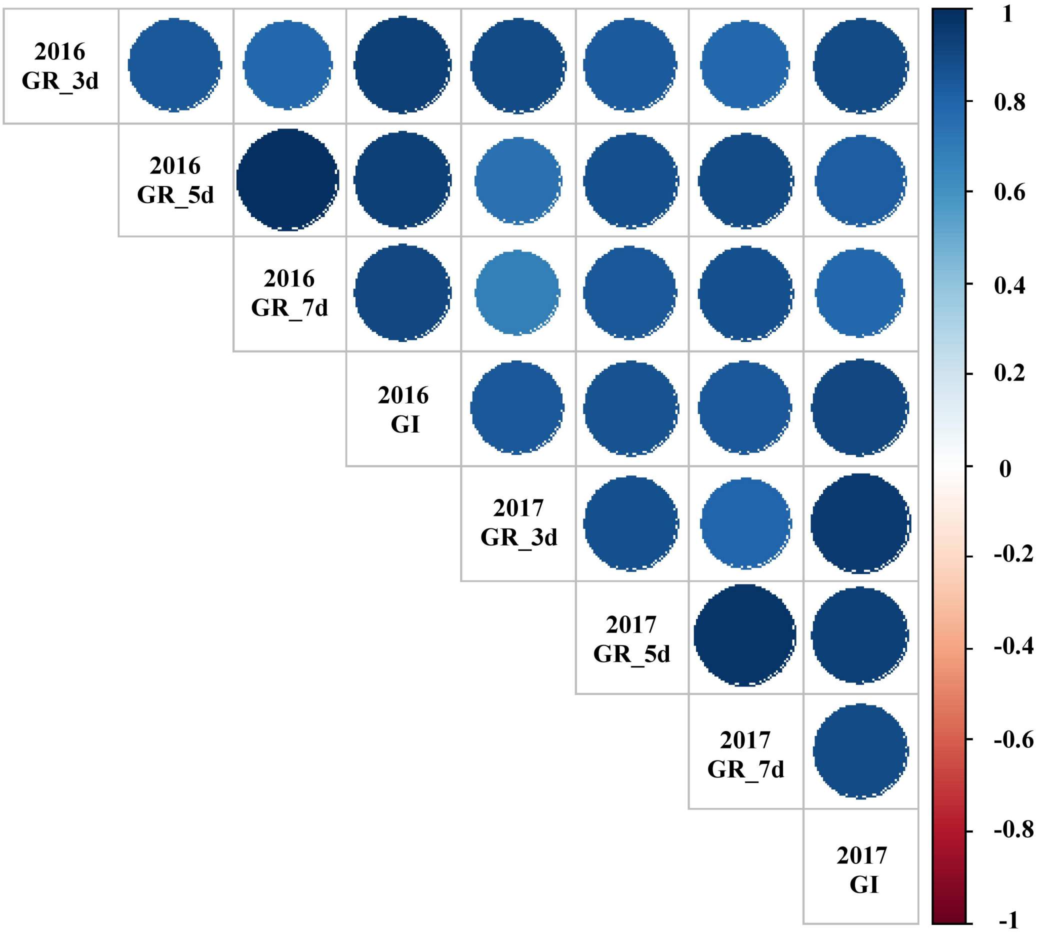

Fig. S1. Pairwise correlation analysis of GRs at 3, 5, and 7 DAI and GIs between the two years.

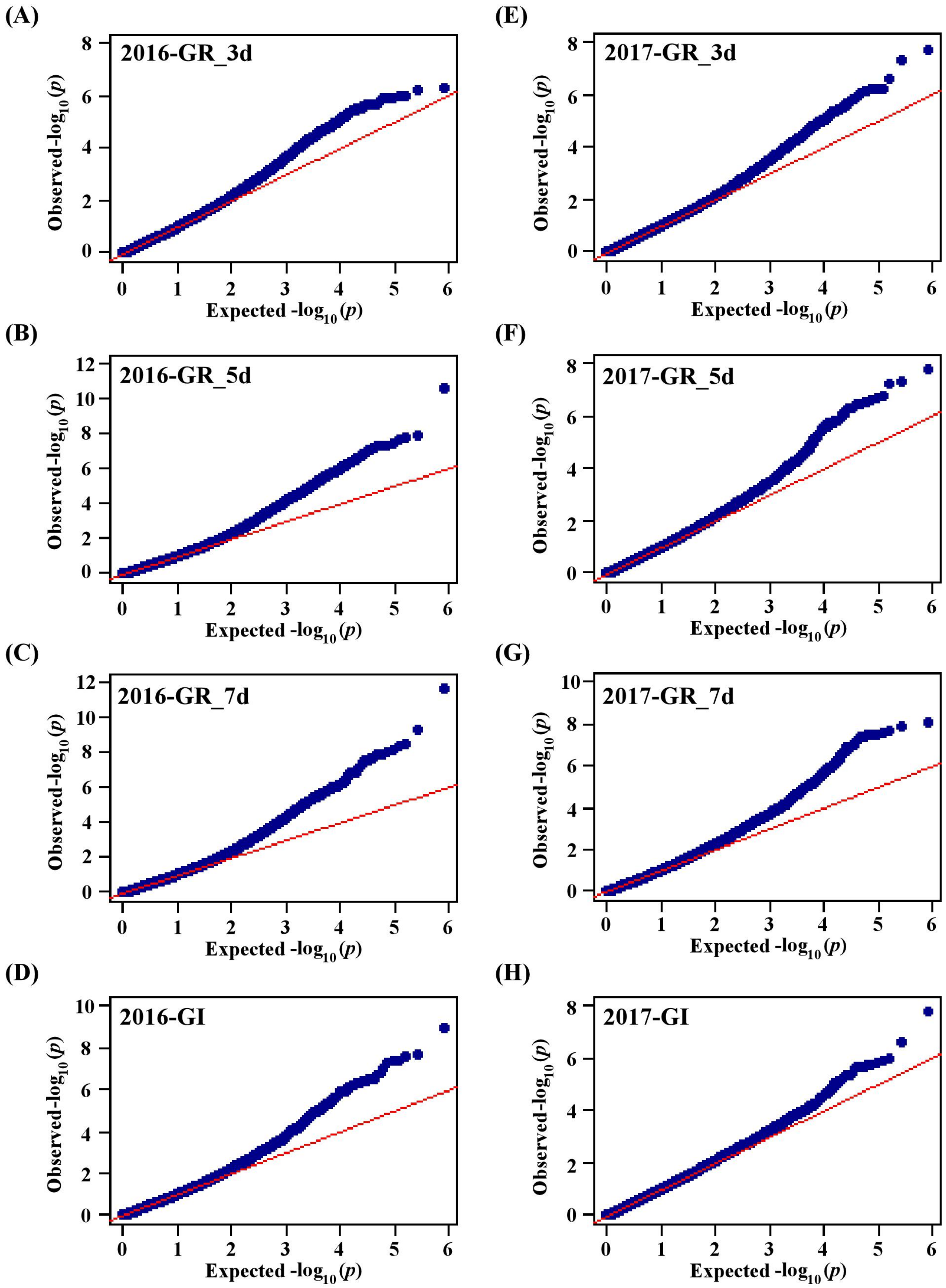

Fig. S2. Quantile-quantile plots of GRs at 3, 5, and 7 DAI and GIs. Quantile-quantile plots of GR_3d, GR_5d, GR_7d and GI in 2016 (A-D) and 2017 (E-H).

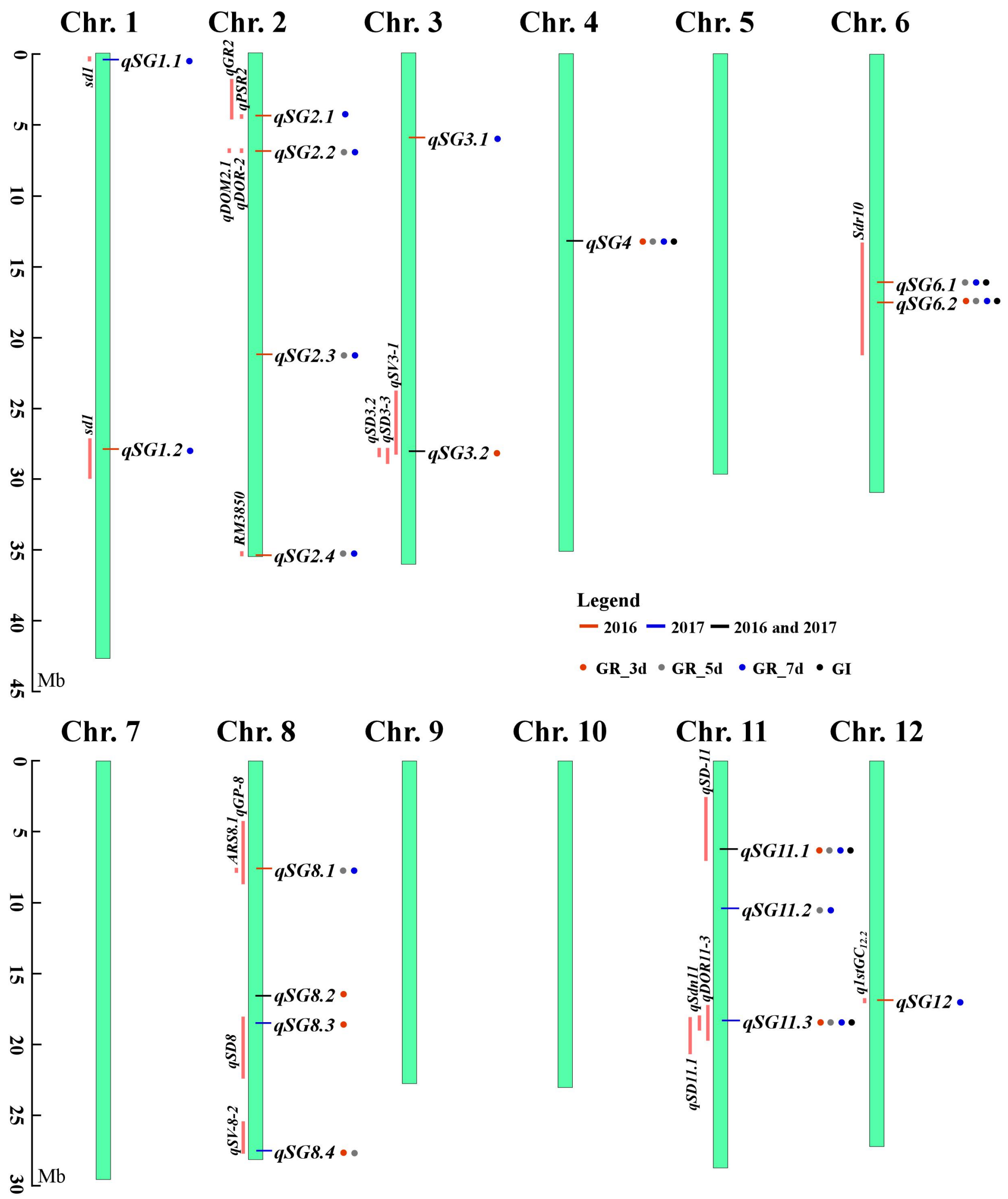

Fig. S3. Distribution of 19 QTLs on 12 chromosomes according to physical position. The QTLs and their associated traits are displayed on the right of the chromosomes with the physical position of the lead SNP of each locus. The colors of the lines indicate the years in which the QTLs were identified. Red, 2016; blue, 2017; and black, both years. The colors of dots indicate the traits for which the QTLs were identified. Red, GR_3d; Gray, GR_5d; Blue, GR_7d; and black, GI. The colocalization of known QTLs are shown in the light red column on the left of the chromosomes according to the physical interval.

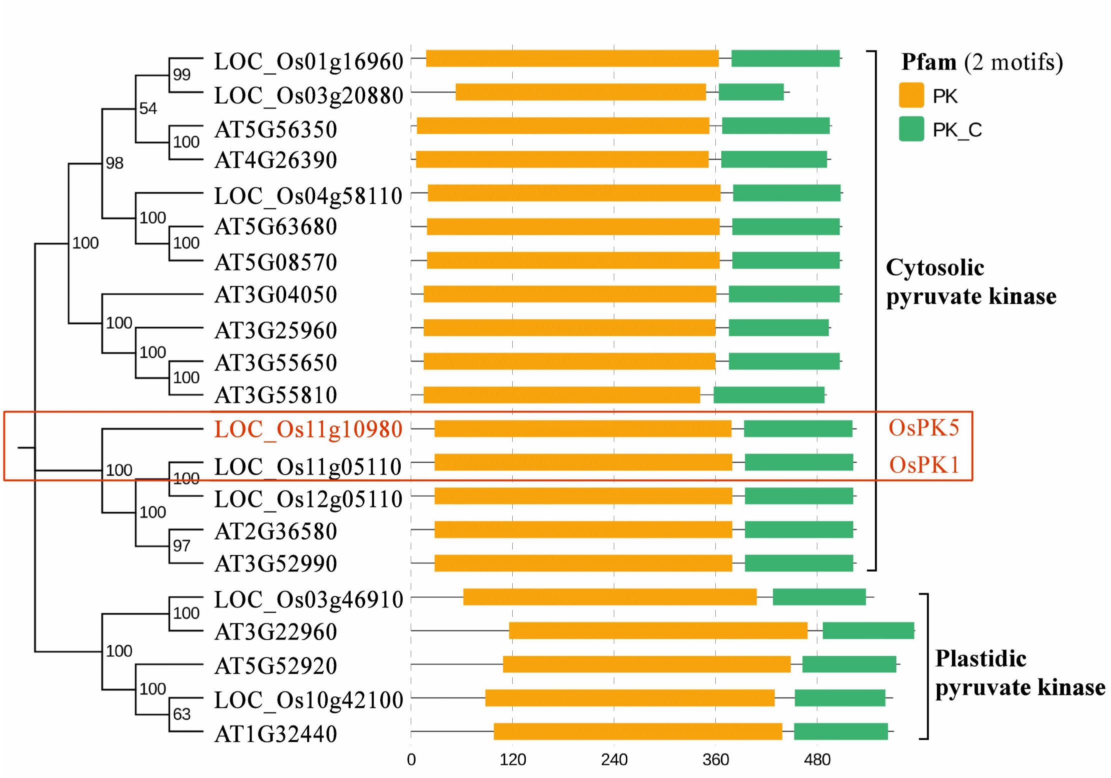

Fig. S4. Phylogenetic tree analyses of pyruvate kinases in rice and *Arabidopsis*.

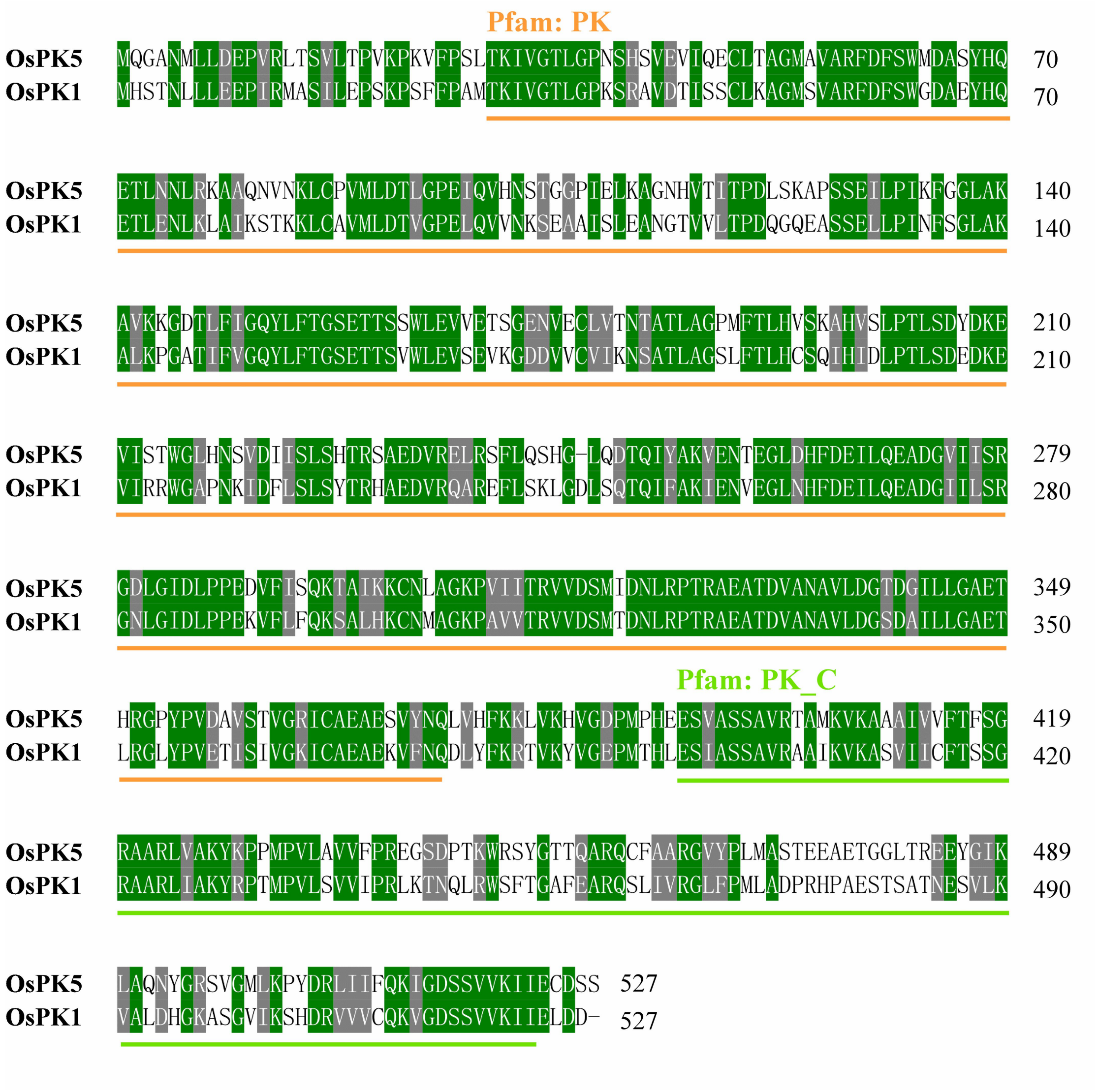

Fig. S5. Comparison of amino acid sequences between OsPK5 and OsPK1 in rice.

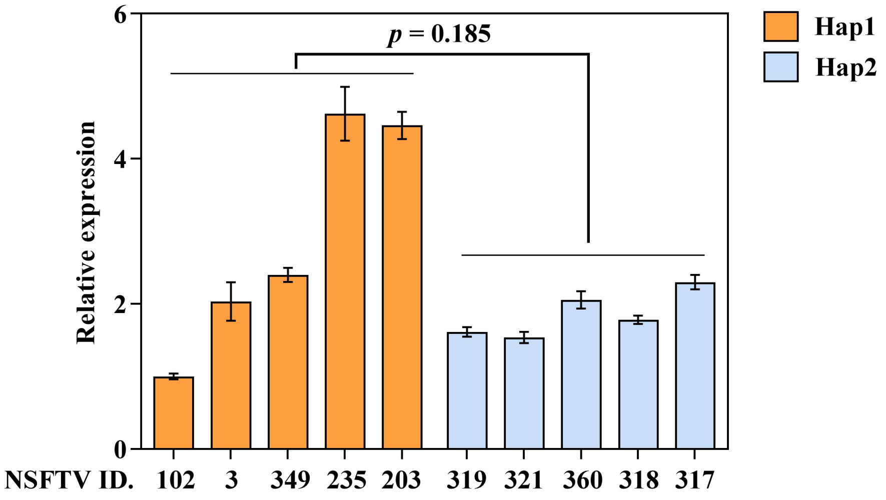

Fig. S6. The expression levels of *OsPK5* in the two haplotypes during seed germination as determined by qRT-PCR with *18S* ribosomal RNA as the reference gene. Differences between haplotypes were analyzed by Student's *t*-test.

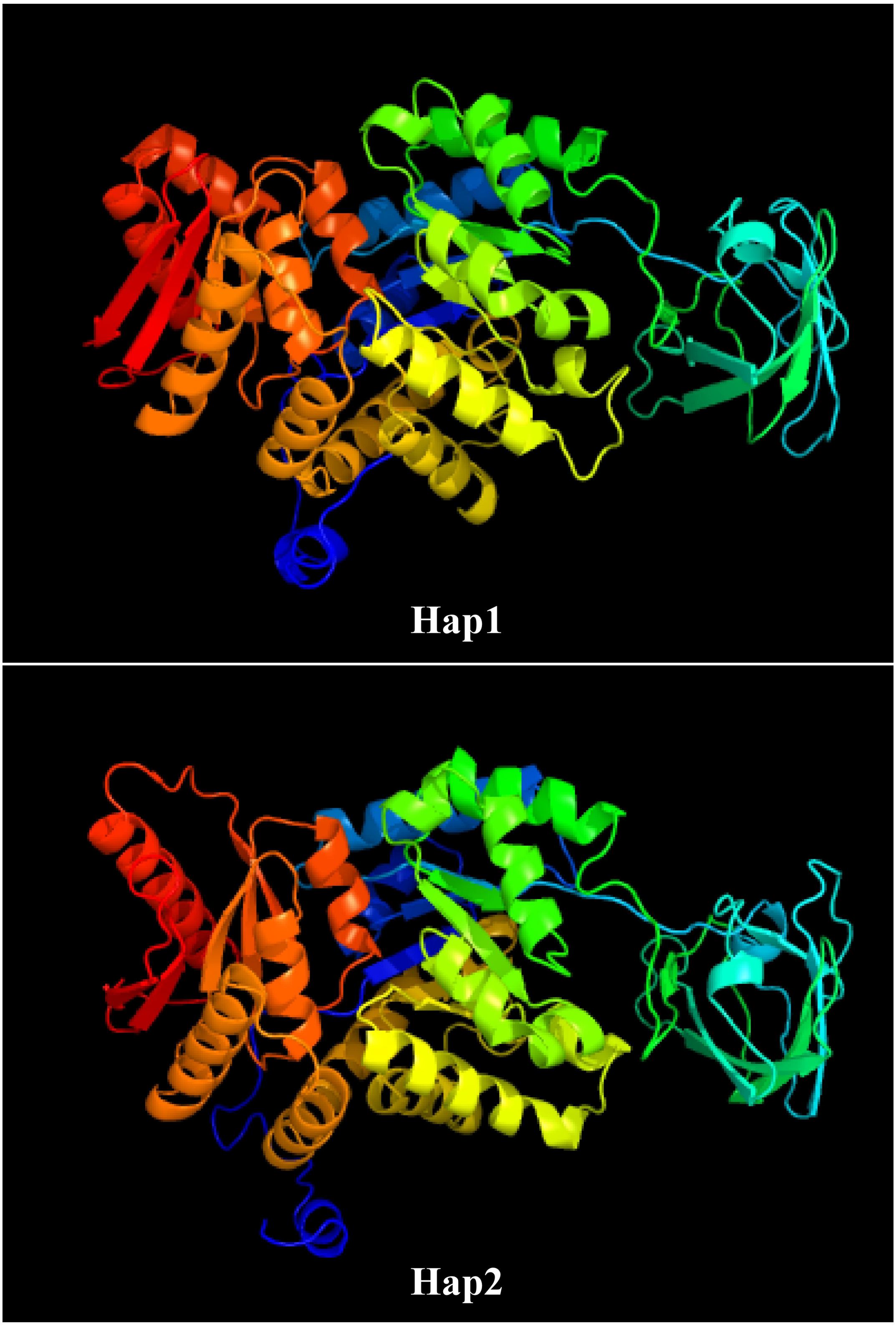

Fig. S7. Protein structure prediction of two different haplotypes of OsPK5 using Phyre2.

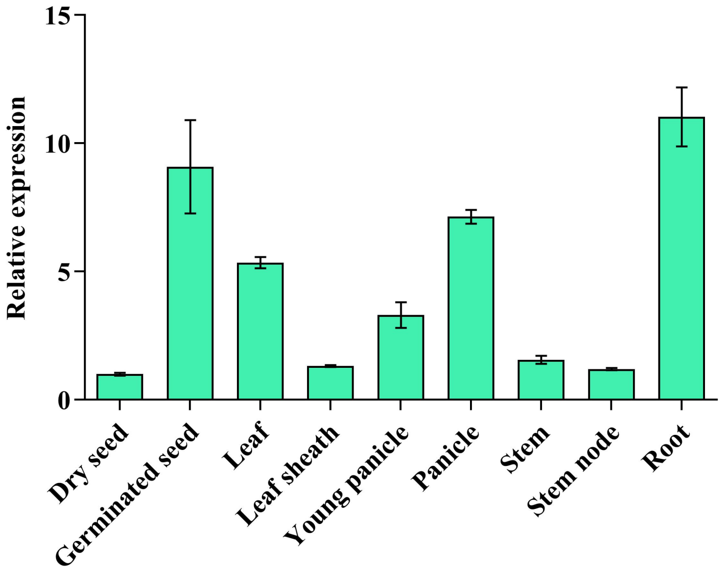

Fig. S8. The expression levels of *OsPK5* in different tissues as determined by qRT-PCR with *18S* ribosomal RNA as the reference gene.

```
WT        MQGANMLLDEPVRLTSVLTPVKPKVFPSLTKIVGTLGPNSHSVEVIQECLTAGMAVARFDFSWMDASYHQ
ospk5-1   MQGANMLLDEPVRLTSVLTPVKPKVFPSLTK--------------MLGPLGPTHTPSRLFKNASLLGWQS
ospk5-2   MQGANMLLDEPVRLTSVLTPVKPKVFPSLT--------NSHSVEVIQECLTAGMAVARFDFSWMDASYHQ
ospk5-3   MQGANMLLDEPVRLTSVLTPVKPKVFPSCWD--------------PWAQLTLRRGYSRMPHCWDGSRTV-

WT        ETLNNLRKAAQNVNKLCPVMLDTLGPEIQVHNSTGGPIELKAGNHVTITPDLSKAPSSEILPIKFGGLAK
ospk5-1   HGLISHGWMLHIIRRPLTI---------------------------------------------------
ospk5-2   ETLNNLRKAAQNVNKLCPVMLDTLGPEIQVHNSTGGPIELKAGNHVTITPDLSKAPSSEILPIKLVVLQK
ospk5-3   ----------------------------------------------------------------------

WT        AVKKGDTLFIGQYLFTGSETTSSWLEVVETSGENVECLVTNTATLAGPMFTLHVSKAHVSLPTLSDYDKE
ospk5-1   ----------------------------------------------------------------------
ospk5-2   LSRRVILFLLVNISSQEVKQHLRGLRLWKLLEKMLNVL--------------------------------
ospk5-3   ----------------------------------------------------------------------

WT        VISTWGLHNSVDIISLSHTRSAEDVRELRSFLQSHGLQDTQIYAKVENTEGLDHFDEILQEADGVIISRG
ospk5-1   ----------------------------------------------------------------------
ospk5-2   ----------------------------------------------------------------------
ospk5-3   ----------------------------------------------------------------------

WT        DLGIDLPPEDVFISQKTAIKKCNLAGKPVIITRVVDSMIDNLRPTRAEATDVANAVLDGTDGILLGAETH
ospk5-1   ----------------------------------------------------------------------
ospk5-2   ----------------------------------------------------------------------
ospk5-3   ----------------------------------------------------------------------

WT        RGPYPVDAVSTVGRICAEAESVYNQLVHFKKLVKHVGDPMPHEESVASSAVRTAMKVKAAAIVVFTFSGR
ospk5-1   ----------------------------------------------------------------------
ospk5-2   ----------------------------------------------------------------------
ospk5-3   ----------------------------------------------------------------------

WT        AARLVAKYKPPMPVLAVVFPREGSDPTKWRSYGTTQARQCFAARGVYPLMASTEEAETGGLTREEYGIKL
ospk5-1   ----------------------------------------------------------------------
ospk5-2   ----------------------------------------------------------------------
ospk5-3   ----------------------------------------------------------------------

WT        AQNYGRSVGMLKPYDRLIIFQKIGDSSVVKIIECDSS 527 aa
ospk5-1   -------------------------------------  75 aa
ospk5-2   ------------------------------------- 170 aa
ospk5-3   -------------------------------------  55 aa
```

Fig. S9. Comparison of amino acid sequences between *ospk5* mutants and WT rice.

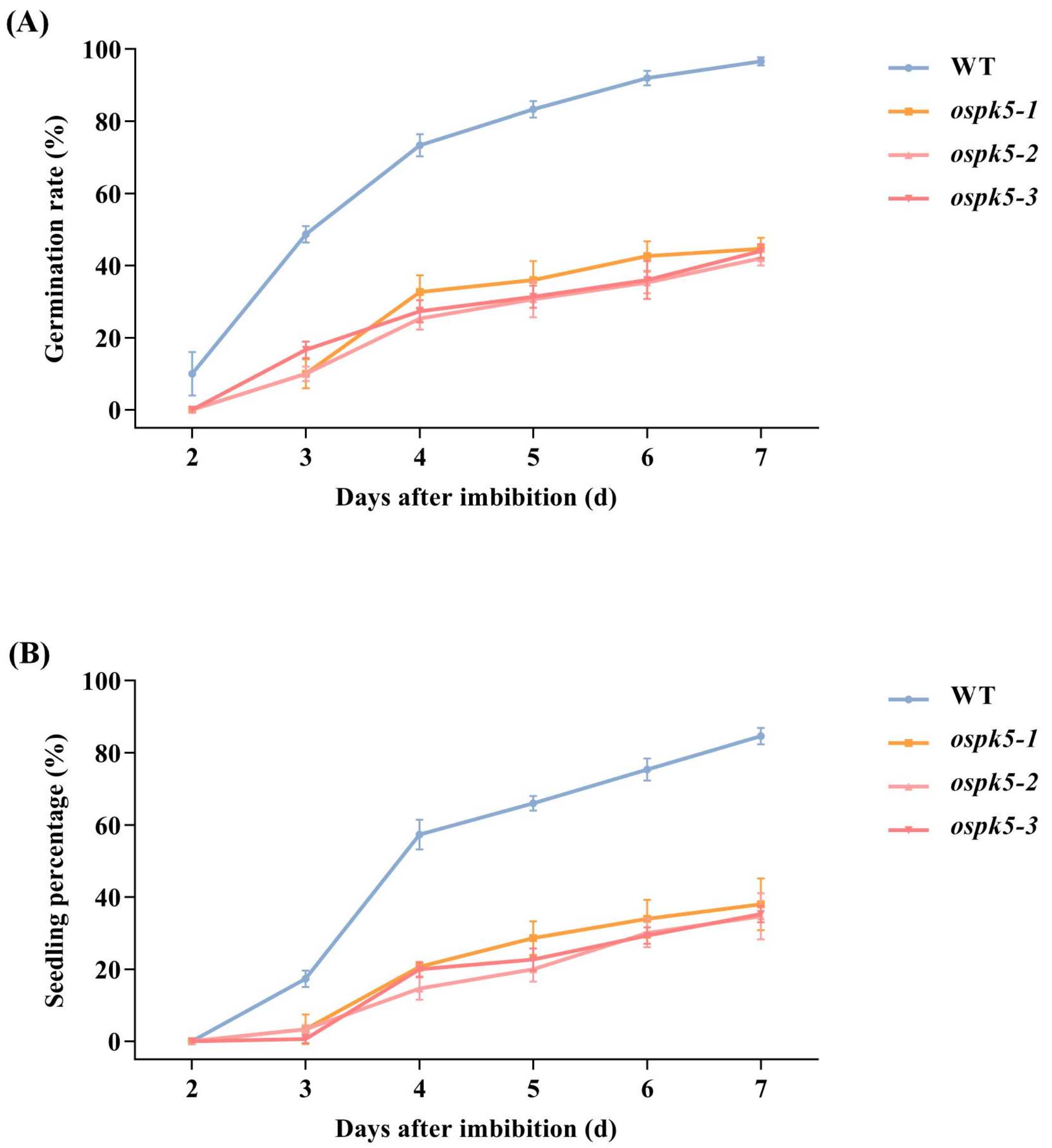

Fig. S10. Dynamic seed germination phenotype between *ospk5* mutants and WT rice. (A) Germination rate. (B) Seedling percentage.

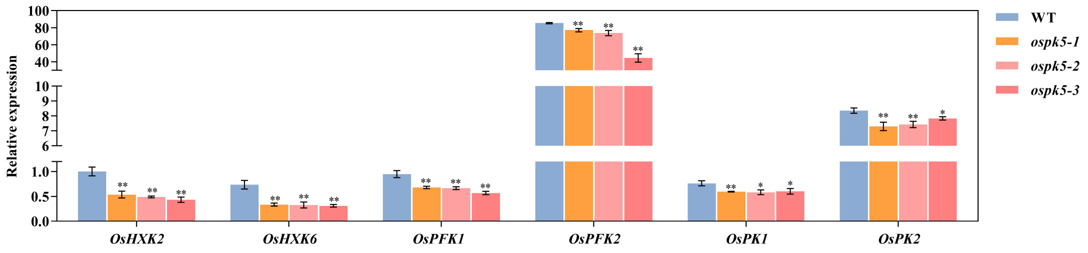

Fig. S11. The expression levels of genes related to the glycolysis pathway as determined by qRT-PCR with *18S* ribosomal RNA as the reference gene. * and ** indicate significant differences between mutant lines and WT at the 5% and 1% levels, respectively.

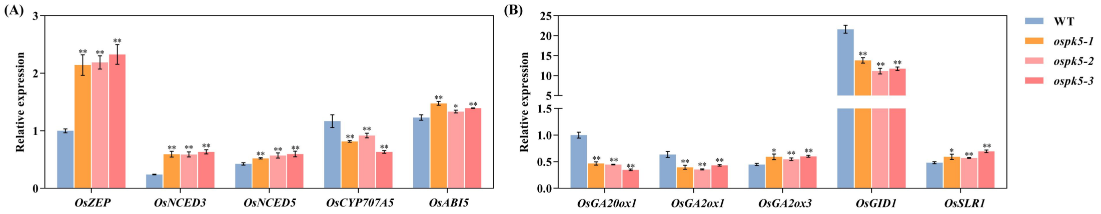

Fig. S12. The expression levels of genes related to the ABA and GA metabolism pathways as determined by qRT-PCR with *18S* ribosomal RNA as the reference gene. (A) ABA metabolism pathway. (B) GA metabolism pathway. * and ** indicate significant differences between mutant lines and WT at the 5% and 1% levels, respectively.

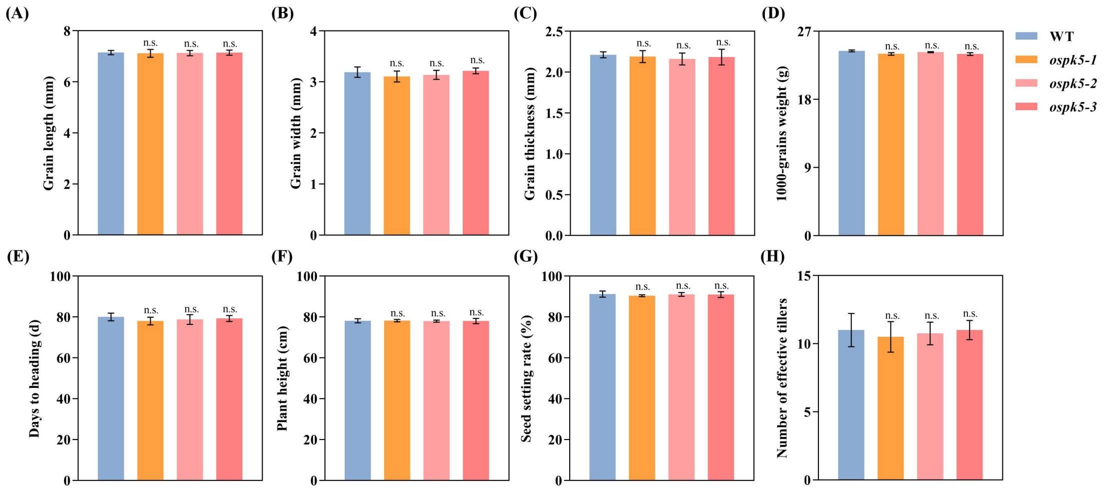

Fig. S13. Comparison of agronomic traits between *ospk5* mutants and WT in rice. (A) Grain length. (B) Grain width. (C) Grain thickness. (D) Grain weight. (E) Days to heading. (F) Plant height. (G) Seed setting rate. (H) Number of effective tillers. n.s. indicates not statistically significant.

Supplemental Table S1-9

Table S1. Information on the 263 rice accessions used for the association analysis.

NSFTV ID.	Subgroups	Accession name	Country of origin	2016				2017			
				GR_3d	GR_5d	GR_7d	GI	GR_3d	GR_5d	GR_7d	GI
NSFTV1	*TEJ*	Agostano	Italy	92	96	98	17	88	98	99	18
NSFTV3	*IND*	Ai-Chiao-Hong	China	93	99	99	19	96	97	98	23
NSFTV4	*AUS*	NSF-TV 4	India	3	33	43	5	14	31	50	6
NSFTV5	*ARO*	NSF-TV 5	India	NA	NA	NA	NA	98	98	98	24
NSFTV6	*AUS*	ARC 7229	India	10	62	75	8	10	65	75	9
NSFTV7	*TRJ*	Arias	Indonesia	2	11	20	2	31	74	85	12
NSFTV8	*TRJ*	Asse Y Pung	Philippines	59	97	100	15	92	99	100	17
NSFTV10	*TEJ*	Baghlani Nangarhar	Afghanistan	3	44	59	6	21	89	96	12
NSFTV13	*AUS*	NSF-TV 13	Pakistan	82	93	95	16	NA	NA	NA	NA
NSFTV15	*TEJ*	Beonjo	South Korea	NA	NA	NA	NA	83	99	99	17
NSFTV17	*IND*	Binulawan	Philippines	71	96	98	16	92	100	100	17
NSFTV18	*AUS*	BJ 1	India	100	100	100	25	100	100	100	25
NSFTV21	*IND*	Byakkoku Y 5006 Seln	Australia	98	100	100	23	97	100	100	24
NSFTV22	*TRJ*	Caawa/Fortuna 6-103-15	China	NA	NA	NA	NA	98	98	99	23
NSFTV23	*TRJ*	Canella De Ferro	Brazil	NA	NA	NA	NA	89	99	100	17

NSFTV25	*TRJ*	Carolina Gold	United States	NA	NA	NA	NA	97	100	100	23
NSFTV26	*TRJ*	Carolina Gold Sel	United States	NA	NA	NA	NA	98	98	98	22
NSFTV27	*TRJ*	NSF-TV 27	Pakistan	97	99	99	18	NA	NA	NA	NA
NSFTV29	*IND*	Chau	Vietnam	91	96	96	16	98	99	99	23
NSFTV30	*IND*	Chiem Chanh	Vietnam	99	100	100	23	96	99	99	21
NSFTV32	*TEJ*	Chodongji	South Korea	97	100	100	21	99	99	99	21
NSFTV33	*AUS*	Chuan 4	China	0	3	7	1	1	24	38	4
NSFTV34	*IND*	NSF-TV 34	India	82	90	90	17	NA	NA	NA	NA
NSFTV36	*TEJ*	CS-M3	United States-CA	57	93	95	14	36	90	98	13
NSFTV39	*ADMIX*	NSF-TV 39	Bangladesh	76	98	98	16	76	94	96	16
NSFTV43	*IND*	Dee Geo Woo Gen	China	99	99	99	21	99	99	99	20
NSFTV44	*AUS*	Dhala Shaitta	Bangladesh	43	62	65	10	56	74	77	12
NSFTV46	*TRJ*	Dourado Agulha	Brazil	97	100	100	19	97	100	100	20
NSFTV49	*AUS*	DV85	Bangladesh	2	3	3	0	2	6	6	1
NSFTV56	*TEJ*	Geumobyeo	South Korea	54	90	94	14	NA	NA	NA	NA
NSFTV57	*IND*	NSF-TV 57	Iran	95	97	99	19	92	97	99	18
NSFTV58	*AUS*	Ghati Kamma Nangarhar	Afghanistan	39	80	85	12	55	75	80	14
NSFTV60	*ADMIX*	Gotak Gatik	Indonesia	0	0	1	0	0	18	34	3
NSFTV62	*TEJ*	Gyehwa 3	South Korea	20	96	98	13	5	34	73	7
NSFTV63	*TEJ*	Haginomae Mochi	Japan	42	100	100	14	NA	NA	NA	NA

NSFTV65	*TRJ*	Honduras	Honduras	99	100	100	20	98	100	100	21
NSFTV67	*TEJ*	Hu Lo Tao	China	NA	NA	NA	NA	98	99	99	22
NSFTV69	*TRJ*	IAC 25	Brazil	99	99	99	23	NA	NA	NA	NA
NSFTV70	*TRJ*	Iguape Cateto	Haiti	NA	NA	NA	NA	99	99	99	21
NSFTV71	*IND*	IR 36	Philippines	89	97	99	20	83	96	99	20
NSFTV72	*IND*	IR 8	Philippines	100	100	100	17	92	99	100	17
NSFTV73	*TRJ*	IRAT 177	French Guiana	NA	NA	NA	NA	100	100	100	19
NSFTV74	*IND*	IRGA 409	Brazil	97	100	100	24	97	98	98	24
NSFTV76	*IND*	Jaya	India	99	100	100	18	98	98	98	22
NSFTV77	*IND*	JC149	India	3	27	34	4	6	17	17	2
NSFTV78	*AUS*	Jhona 349	India	71	83	85	16	84	96	96	17
NSFTV79	*TEJ*	Jouiku 393G	Japan	15	88	96	12	17	54	69	8
NSFTV81	*AUS*	Kalamkati	India	NA	NA	NA	NA	25	64	73	10
NSFTV83	*TEJ*	Kamenoo	Japan	35	86	94	13	NA	NA	NA	NA
NSFTV84	*TRJ*	Kaniranga	Indonesia	29	71	75	10	23	72	75	10
NSFTV85	*AUS*	Kasalath	India	37	53	53	10	91	96	97	19
NSFTV87	*ADMIX*	Keriting Tingii	Indonesia	61	74	77	12	90	100	100	17
NSFTV90	*IND*	Kiang-Chou-Chiu	China	93	95	95	21	98	99	99	24
NSFTV91	*TEJ*	Kibi	Japan	NA	NA	NA	NA	97	100	100	20
NSFTV92	*TRJ*	Kinastano	Philippines	NA	NA	NA	NA	99	99	99	19

NSFTV94	*TEJ*	Koshihikari	Japan	1	35	58	6	2	8	30	3
NSFTV99	*TRJ*	LAC 23	Liberia	76	97	97	16	90	96	96	19
NSFTV100	*ADMIX*	Lacrosse	United States	95	99	99	19	95	100	100	20
NSFTV101	*TRJ*	Lemont	United States	97	100	100	19	95	99	99	20
NSFTV102	*IND*	Leung Pratew	Thailand	99	99	99	20	96	98	98	19
NSFTV103	*TEJ*	Luk Takhar	Afghanistan	69	94	97	15	62	98	100	16
NSFTV104	*TEJ*	Mansaku	Japan	17	99	100	13	46	91	99	13
NSFTV105	*AUS*	Mehr	Iran	3	8	24	2	1	25	42	4
NSFTV106	*IND*	Ming Hui	China	50	65	69	12	59	77	77	14
NSFTV107	*TRJ*	NSF-TV 107	Bangladesh	92	100	100	20	NA	NA	NA	NA
NSFTV108	*TRJ*	Moroberekan	Guinea	NA	NA	NA	NA	85	99	100	17
NSFTV114	*ADMIX*	Nova	United States	87	95	99	17	96	99	99	19
NSFTV115	*TEJ*	NPE 835	Pakistan	78	95	95	16	78	90	90	18
NSFTV116	*TRJ*	NSF-TV 116	Pakistan	99	99	99	24	99	99	99	23
NSFTV117	*IND*	O-Luen-Cheung	China	87	87	87	20	99	100	100	25
NSFTV118	*TEJ*	Oro	Chile	70	93	95	15	NA	NA	NA	NA
NSFTV120	*TRJ*	OS6	Nigeria	99	100	100	19	85	98	98	16
NSFTV121	*TEJ*	Ostiglia	Argentina	20	89	94	12	NA	NA	NA	NA
NSFTV122	*TRJ*	Padi Kasalle	Indonesia	47	92	96	14	52	80	88	13
NSFTV123	*IND*	Pagaiyahan	China	91	100	100	18	92	99	100	19

NSFTV124	*ARO*	Pankhari 203	India	97	99	99	21	96	100	100	19
NSFTV125	*IND*	Pao-Tou-Hung	China	99	99	99	23	100	100	100	24
NSFTV129	*IND*	Peh-Kuh	China	95	99	99	20	95	99	99	19
NSFTV130	*IND*	Peh-Kuh-Tsao-Tu	China	91	98	100	19	91	98	100	18
NSFTV131	*AUS*	Phudugey	Bhutan	NA	NA	NA	NA	14	34	40	5
NSFTV132	*IND*	Rathuwee	Sri Lanka	95	97	97	17	94	99	99	20
NSFTV133	*TEJ*	Rikuto Kemochi	Japan	93	95	95	19	87	90	92	19
NSFTV134	*TEJ*	Romeo	Italy	81	89	91	16	89	95	97	20
NSFTV137	*IND*	RTS14	Vietnam	95	100	100	23	88	99	99	20
NSFTV139	*TRJ*	S4542A3-49B-2B12	United States	96	97	97	21	NA	NA	NA	NA
NSFTV140	*ADMIX*	Saturn	United States	94	97	97	21	95	96	96	21
NSFTV143	*TEJ*	Shinriki	Japan	7	89	98	11	6	91	98	11
NSFTV149	*TRJ*	Sinaguing	Philippines	15	76	88	11	12	65	84	9
NSFTV151	*TEJ*	Suweon	Korea	11	83	99	12	10	52	74	8
NSFTV152	*AUS*	T 1	India	3	31	41	4	6	32	40	5
NSFTV153	*AUS*	T26	India	3	18	23	3	9	40	53	6
NSFTV154	*TEJ*	Ta Hung Ku	China	47	79	87	13	73	95	98	17
NSFTV155	*TEJ*	Ta Mao Tsao	China	0	3	11	1	0	9	23	2
NSFTV156	*IND*	Taichung Native 1	China	100	99	99	23	100	100	100	24
NSFTV157	*TEJ*	Tainan Iku 487	China	NA	NA	NA	NA	97	99	99	20

NSFTV158	*TEJ*	Taipei 309	China	91	98	99	17	NA	NA	NA	NA
NSFTV160	*ARO*	NSF-TV 160	Iran	0	0	0	0	0	4	11	1
NSFTV161	*IND*	TeQing	China	99	99	99	23	99	100	100	23
NSFTV169	*TEJ*	WC 6	China	74	95	95	16	85	96	97	18
NSFTV171	*IND*	ZHE 733	China	97	100	100	18	98	99	99	21
NSFTV172	*IND*	Zhenshan 2	China	97	99	99	19	97	100	100	19
NSFTV174	*TRJ*	Azucena	Philippines	83	95	97	16	65	85	90	15
NSFTV177	*TEJ*	24869	France	59	83	90	13	49	94	96	15
NSFTV178	*AUS*	ARC 6578	India	10	24	25	3	5	21	35	4
NSFTV179	*TEJ*	Bellardone	France	78	93	97	15	NA	NA	NA	NA
NSFTV180	*TEJ*	Benllok	Peru	69	91	95	15	84	96	99	17
NSFTV183	*TRJ*	Boa Vista	El Salvador	98	99	100	19	99	100	100	23
NSFTV185	*TRJ*	British Honduras Creole	Belize	100	100	100	22	100	100	100	23
NSFTV187	*TRJ*	C57-5043	United States	60	86	90	13	NA	NA	NA	NA
NSFTV188	*TRJ*	Coppocina	Bulgaria	NA	NA	NA	NA	96	99	99	20
NSFTV189	*IND*	Criollo La Fria	Venezuela	100	100	100	24	99	99	99	24
NSFTV191	*ARO*	Dom Zard	Iran	3	34	50	5	NA	NA	NA	NA
NSFTV199	*TRJ*	NSF-TV 199	Bolivia	NA	NA	NA	NA	93	97	97	19
NSFTV200	*AUS*	P 737	Pakistan	41	43	43	9	26	28	28	6
NSFTV201	*TRJ*	Pate Blanc Mn 1	Cote D'Ivoire	91	98	99	20	90	100	100	22

NSFTV203	*IND*	Radin Ebos 33	Malaysia	87	100	100	19	89	100	100	20
NSFTV209	*IND*	Tchibanga	Gabon	97	99	99	19	96	99	99	21
NSFTV211	*ADMIX*	Tokyo Shino Mochi	Japan	NA	NA	NA	NA	85	98	98	17
NSFTV213	*TRJ*	WC 3397	Jamaica	21	52	63	8	NA	NA	NA	NA
NSFTV214	*TRJ*	WC 4419	Honduras	NA	NA	NA	NA	34	74	81	11
NSFTV215	*TRJ*	WC 4443	Bolivia	NA	NA	NA	NA	76	100	100	20
NSFTV217	*ADMIX*	YRL-1	Australia	41	91	98	13	58	90	95	15
NSFTV218	*ADMIX*	PI 298967-1	Australia	NA	NA	NA	NA	75	99	99	16
NSFTV221	*ARO*	Sadri Belyi	Azerbaijan	1	1	4	0	NA	NA	NA	NA
NSFTV222	*IND*	Paraiba Chines Nova	Brazil	99	99	99	24	100	100	100	25
NSFTV223	*TRJ*	Priano Guaira	Brazil	99	99	99	23	NA	NA	NA	NA
NSFTV225	*TEJ*	Biser 1	Bulgaria	66	95	97	16	81	97	97	18
NSFTV226	*TRJ*	IRAT 44	Burkina Faso	NA	NA	NA	NA	91	96	96	18
NSFTV227	*ADMIX*	Riz Local	Burkina Faso	0	11	19	2	NA	NA	NA	NA
NSFTV228	*AUS*	CA 902/B/2/1	Chad	73	93	94	15	33	72	77	11
NSFTV231	*IND*	Hunan Early Dwarf No. 3	China	98	99	99	20	98	99	99	21
NSFTV234	*IND*	Aijiaonante	China	99	99	99	19	93	99	100	22
NSFTV235	*IND*	Sze Guen Zim	China	94	97	97	23	97	99	100	24
NSFTV236	*ADMIX*	WC 521	China	21	90	99	12	NA	NA	NA	NA
NSFTV239	*TRJ*	WAB 502-13-4-1	Cote D'Ivoire	61	95	99	15	NA	NA	NA	NA

NSFTV240	*TRJ*	WAB 501-11-5-1	Cote D'Ivoire	86	95	95	18	79	90	90	16
NSFTV241	*IND*	ECIA76-S89-1	Cuba	97	100	100	19	64	91	92	16
NSFTV242	*TRJ*	27	Dominican Republic	65	68	69	15	99	99	99	21
NSFTV245	*TEJ*	Sab Ini	Egypt	11	47	53	7	38	74	82	12
NSFTV247	*TEJ*	Desvauxii	Former Soviet Union	62	90	93	15	75	97	97	17
NSFTV248	*TEJ*	Caucasica	Former Soviet Union	23	78	87	11	NA	NA	NA	NA
NSFTV249	*ADMIX*	Pirinae 69	Former Yugoslavia	70	89	89	15	69	86	86	15
NSFTV252	*IND*	Djimoron	Guinea	31	41	43	7	64	77	78	13
NSFTV255	*IND*	Pai Hok Glutinous	Hong Kong	NA	NA	NA	NA	98	99	99	22
NSFTV256	*TEJ*	Romanica	Hungary	NA	NA	NA	NA	80	96	96	17
NSFTV257	*TEJ*	Agusita	Hungary	100	99	99	19	NA	NA	NA	NA
NSFTV259	*ADMIX*	Sadri Tor Misri	Iran	5	25	33	4	41	69	70	10
NSFTV260	*ARO*	NSF-TV 260	Iran	52	100	100	14	NA	NA	NA	NA
NSFTV261	*AUS*	Shim Balte	Iraq	67	79	81	15	NA	NA	NA	NA
NSFTV263	*TEJ*	Maratelli	Italy	86	92	93	16	95	99	99	19
NSFTV264	*ADMIX*	Baldo	Italy	NA	NA	NA	NA	74	93	97	15

NSFTV266	*ADMIX*	Hiderisirazu	Japan	74	91	97	16	71	81	83	14
NSFTV267	*TEJ*	Hatsunishiki	Japan	26	93	95	13	34	98	99	14
NSFTV272	*ADMIX*	923	Madagascar	69	93	97	15	51	80	86	14
NSFTV273	*ADMIX*	Varyla	Madagascar	100	100	100	19	82	97	98	18
NSFTV274	*TRJ*	Padi Pagalong	Malaysia	NA	NA	NA	NA	69	99	99	16
NSFTV275	*TEJ*	Sri Malaysia Dua	Malaysia	58	99	99	15	20	84	93	12
NSFTV276	*AUS*	Kaukau	Mali	0	5	9	1	14	67	80	10
NSFTV277	*TEJ*	Gambiaka Sebela	Mali	49	93	94	14	30	90	96	13
NSFTV280	*ADMIX*	Saku	Mongolia	85	99	99	16	21	71	92	11
NSFTV281	*TEJ*	Patna	Morocco	55	87	96	14	11	56	77	9
NSFTV282	*TEJ*	Triomphe Du Maroc	Morocco	73	99	99	17	42	85	94	13
NSFTV283	*TEJ*	Chibica	Mozambique	73	94	95	15	49	94	98	14
NSFTV284	*IND*	IR-44595	Nepal	95	99	99	22	91	95	96	21
NSFTV285	*TRJ*	Tox 782-20-1	Nigeria	92	100	100	19	95	99	99	21
NSFTV287	*TEJ*	Zerawchanica Karatalski	Poland	NA	NA	NA	NA	75	88	90	16
NSFTV288	*TEJ*	Italica Carolina	Poland	73	98	98	16	25	82	94	12
NSFTV290	*TEJ*	Amposta	Puerto Rico	15	65	84	10	6	31	52	6
NSFTV291	*TEJ*	Toploea 70/76	Romania	23	75	90	11	14	62	82	10
NSFTV292	*TEJ*	Stegaru 65	Romania	29	71	87	11	30	61	81	10
NSFTV293	*ADMIX*	TOg 7178	Senegal	0	15	15	2	3	11	11	1

NSFTV296	*TEJ*	Dosel	Spain	84	97	97	18	91	97	97	17
NSFTV297	*TEJ*	Bahia	Spain	90	95	97	16	45	95	99	14
NSFTV299	*IND*	SML 242	Suriname	93	95	95	19	92	96	96	22
NSFTV300	*TEJ*	Sml Kapuri	Suriname	94	96	97	19	74	90	92	16
NSFTV302	*TEJ*	WIR 3039	Tajikistan	75	99	99	16	NA	NA	NA	NA
NSFTV303	*TEJ*	Kihogo	Tanzania	63	97	98	15	NA	NA	NA	NA
NSFTV304	*IND*	519	Uruguay	84	91	92	18	72	88	92	16
NSFTV305	*ADMIX*	Doble Carolina Rinaldo Barsani	Uruguay	79	99	99	17	74	95	96	15
NSFTV307	*TEJ*	Uzbekskij 2	Uzbekistan	72	91	94	16	53	97	99	14
NSFTV308	*TRJ*	Llanero 501	Venezuela	NA	NA	NA	NA	99	100	100	22
NSFTV309	*TRJ*	Manzano	Zaire	100	100	100	20	98	99	99	19
NSFTV311	*TEJ*	56-122-23	Thailand	31	97	99	14	9	75	98	11
NSFTV313	*IND*	BR24	Bangladesh	74	81	84	16	81	91	91	19
NSFTV314	*AUS*	CTG 1516	Bangladesh	7	22	27	3	11	29	31	4
NSFTV315	*IND*	Dawebyan	Myanmar	90	98	98	19	94	97	97	20
NSFTV316	*AUS*	DD 62	Bangladesh	3	7	7	1	2	3	3	0
NSFTV317	*AUS*	DJ 123	Bangladesh	0	1	1	0	0	2	4	0
NSFTV318	*AUS*	DJ 24	Bangladesh	0	0	1	0	0	3	4	0
NSFTV319	*AUS*	DK 12	Bangladesh	0	0	0	0	0	3	3	0

NSFTV320	*AUS*	DM 43	Bangladesh	3	10	13	2	2	9	12	1
NSFTV321	*AUS*	DM 56	Bangladesh	0	3	3	0	0	8	18	2
NSFTV322	*AUS*	DM 59	Bangladesh	2	3	3	0	5	8	9	1
NSFTV323	*AUS*	DNJ 140	Bangladesh	25	87	91	12	12	55	70	8
NSFTV324	*AUS*	DV 123	Bangladesh	0	7	10	1	0	10	24	2
NSFTV325	*IND*	EMATA A 16-34	Myanmar	98	100	100	23	NA	NA	NA	NA
NSFTV326	*AUS*	Ghorbhai	Bangladesh	5	14	14	2	3	11	23	2
NSFTV327	*AUS*	Goria	Bangladesh	57	80	81	12	NA	NA	NA	NA
NSFTV328	*AUS*	Jamir	Bangladesh	99	99	99	23	99	99	99	23
NSFTV329	*AUS*	Kachilon	Bangladesh	5	22	22	3	5	14	23	3
NSFTV330	*AUS*	Khao Pahk Maw	Thailand	82	96	97	17	65	91	96	16
NSFTV332	*ADMIX*	KPF-16	Bangladesh	NA	NA	NA	NA	100	100	100	25
NSFTV334	*TEJ*	Lomello	Thailand	22	69	85	11	9	25	47	5
NSFTV335	*ADMIX*	Okshitmayin	Myanmar	46	79	85	13	72	94	95	16
NSFTV336	*AUS*	Paung Malaung	Myanmar	31	50	51	8	0	13	23	2
NSFTV337	*IND*	Sabharaj	Bangladesh	97	99	99	23	98	99	99	24
NSFTV340	*ADMIX*	Berenj	Afghanistan	29	35	39	7	64	72	72	14
NSFTV341	*AUS*	Shirkati	Afghanistan	68	77	79	14	88	99	99	17
NSFTV342	*TRJ*	Cenit	Argentina	96	96	96	20	99	99	99	23
NSFTV344	*ADMIX*	Habiganj Boro 6	Bangladesh	23	81	88	12	55	84	89	13

NSFTV345	*AUS*	DZ 193	Bangladesh	1	1	1	0	0	0	0	0
NSFTV346	*AUS*	Karkati 87	Bangladesh	53	77	82	13	84	95	98	19
NSFTV348	*IND*	China 1039	China	93	97	97	17	93	98	98	21
NSFTV349	*IND*	Chang Ch'Sang Hsu Tao	China	93	99	99	23	98	100	100	24
NSFTV350	*TRJ*	Ligerito	Colombia	26	77	86	11	NA	NA	NA	NA
NSFTV352	*TRJ*	Guatemala 1021	Guatemala	96	97	97	23	100	100	100	24
NSFTV353	*AUS*	ARC 10376	India	8	35	45	5	3	7	8	1
NSFTV355	*TEJ*	ASD 1	India	70	95	99	16	56	97	99	15
NSFTV356	*IND*	JC 117	India	93	100	100	20	94	99	99	22
NSFTV357	*AUS*	9524	India	94	100	100	22	25	71	84	11
NSFTV359	*AUS*	Surjamkuhi	India	0	0	0	0	0	1	1	0
NSFTV360	*AUS*	PTB 30	India	0	1	1	0	2	14	20	2
NSFTV363	*TEJ*	Edomen Scented	Japan	17	95	98	13	27	96	96	13
NSFTV364	*ADMIX*	Rikuto Norin 21	Japan	51	91	98	14	29	69	83	11
NSFTV365	*TEJ*	Shirogane	Japan	NA	NA	NA	NA	90	97	97	20
NSFTV367	*ADMIX*	Sanbyang-Daeme	Korea	NA	NA	NA	NA	87	98	98	21
NSFTV368	*TEJ*	Deokjeokjodo	Korea	89	97	99	17	88	90	90	18
NSFTV369	*AUS*	Sathi	Pakistan	84	95	96	21	77	94	94	18
NSFTV370	*AUS*	Coarse	Pakistan	75	88	95	16	30	54	54	9
NSFTV371	*AUS*	Santhi Sufaid	Pakistan	13	29	45	5	3	9	9	1

NSFTV372	*AUS*	Sufaid	Pakistan	24	51	55	7	9	44	49	6
NSFTV373	*ARO*	Lambayeque 1	Peru	3	45	69	7	1	5	12	1
NSFTV375	*TRJ*	Upland	Ponape Island	NA	NA	NA	NA	95	99	99	21
NSFTV376	*ADMIX*	Breviaristata	Portugal	99	99	99	22	94	100	100	18
NSFTV377	*TRJ*	PR 304	Puerto Rico	89	95	95	21	98	99	99	22
NSFTV378	*AUS*	Kalubala Vee	Sri Lanka	NA	NA	NA	NA	54	85	87	14
NSFTV380	*TEJ*	Tainan-Iku No. 512	China	63	95	99	15	41	91	96	13
NSFTV381	*TRJ*	325	China	29	63	72	10	22	62	71	9
NSFTV384	*TRJ*	318	Turkey	96	100	100	18	94	100	100	18
NSFTV385	*IND*	Nira	United States	97	99	99	21	90	95	95	21
NSFTV386	*ADMIX*	Palmyra	United States	95	99	99	22	100	100	100	21
NSFTV387	*ADMIX*	M-202	United States-CA	88	98	99	19	82	97	97	17
NSFTV388	*ADMIX*	Nortai	United States	89	100	100	17	87	100	100	17
NSFTV391	*TRJ*	Della	United States	65	77	79	14	95	99	99	18
NSFTV392	*TRJ*	Edith	United States	NA	NA	NA	NA	96	99	99	21
NSFTV394	*TRJ*	Lady Wright Seln	United States	NA	NA	NA	NA	94	99	99	21
NSFTV395	*TRJ*	OS 6 (WC 10296)	Zaire	83	87	88	18	81	96	98	17
NSFTV618	*ADMIX*	Pecos	United States	57	85	87	13	83	95	95	16
NSFTV620	*IND*	Jasmine85	Philippines	81	96	99	18	79	95	99	18
NSFTV622	*ADMIX*	Bengal	United States	85	100	100	16	44	95	95	14

NSFTV626	*IND*	C101A51	Colombia	88	97	97	17	93	98	98	20
NSFTV628	*TRJ*	Jefferson	United States	NA	NA	NA	NA	94	100	100	19
NSFTV629	*ADMIX*	Panda	United States	87	90	90	17	80	88	89	19
NSFTV633	*IND*	Jing 185-7	China	9	14	17	2	6	12	12	2
NSFTV634	*IND*	Rondo (4484-1693)	China	79	92	95	16	76	91	91	16
NSFTV635	*TRJ*	Azucena	Philipppines	81	98	99	17	63	82	87	14
NSFTV636	*IND*	Sadu Cho	Korea	77	91	93	16	NA	NA	NA	NA
NSFTV639	*TEJ*	Nipponbare	Japan	6	83	99	11	1	12	49	4
NSFTV641	*TEJ*	Tainung 67	China	1	84	95	10	2	68	97	9
NSFTV642	*IND*	Zhenshan 97B	China	99	99	99	21	NA	NA	NA	NA
NSFTV643	*IND*	Minghui 63	China	NA	NA	NA	NA	19	37	41	6
NSFTV644	*IND*	IR64	Philippines	82	95	95	16	82	99	100	17
NSFTV651	*AUS*	Dular	India	33	55	58	8	5	40	47	5

NA represents the missing data due to the lack of the well-filled seeds in that year.

Table S2. Descriptive statistics of GRs at 3, 5, and 7 DAI and GIs for over two years.

Trait	Year	Range	Mean	SD	CV	Heritability (%)	G × E
GR_3d (%)	2016	0.00 ~ 100.00	59.97	36.18	0.60	94.64	***
	2017	0.00 ~ 100.00	62.43	36.72	0.59		
GR_5d (%)	2016	0.00 ~ 100.00	77.79	31.43	0.40	93.15	***
	2017	0.00 ~ 100.00	78.57	30.95	0.39		
GR_7d (%)	2016	0.00 ~ 100.00	80.71	30.34	0.38	93.41	***
	2017	0.00 ~ 100.00	82.34	28.33	0.34		
GI	2016	0.0 ~ 24.75	14.10	6.58	0.47	95.16	***
	2017	0.0 ~ 24.83	14.91	6.83	0.46		

*** indicates $P < 0.001$, G × E indicates interaction of genotype and environment.

Table S3. Details of the significant QTLs for GRs at 3, 5, and 7 DAI and GIs for two years.

QTLs	Years	Traits	Chr.	Lead SNP ID.	Lead SNP position (bp)	Alleles	*P* value	Position range (bp)	Known QTLs	Reference
qSG1.1	2017	GR_7d	1	SNP-1.225119	226120	A/C	1.05E-07	75947-444235	*sd1*	Li *et al.*, 2006
qSG1.2	2016	GR_7d	1	SNP-1.27737252	27738297	G/T	1.61E-06	27626285-27927824	*sd1*	Lee *et al.*, 2005
qSG2.1	2016	GR_7d	2	SNP-2.3974377	3974381	G/A	2.69E-06	3857418-4074381	*qPSR2/qGR2*	Gao *et al.*, 2008; Li *et al.*, 2011
qSG2.2	2016	GR_5d	2	SNP-2.6794373	6794374	C/T	4.61E-08	6651338-7110119	*qDOR-2/qDOM2.1*	Cai *et al.*, 2000; Yuan *et al.*, 2020
	2016	GR_7d	2	SNP-2.6794373	6794374	C/T	3.08E-08			
qSG2.3	2016	GR_5d	2	SNP-2.21335276	21341145	C/T	4.85E-07	21004089-21490577		
	2016	GR_7d	2	SNP-2.21335276	21341145	C/T	1.27E-07			
qSG2.4	2016	GR_5d	2	SNP-2.35625353	35631223	C/A	3.78E-08	35531223-35759739	*RM3850*	Dang *et al.*, 2014
	2016	GR_7d	2	SNP-2.35625353	35631223	C/A	4.06E-10			
qSG3.1	2016	GR_7d	3	SNP-3.5859478	5860479	G/T	1.29E-07	5724606-5960479		
qSG3.2	2017	GR_3d	3	SNP-3.28022106	28029053	G/A	2.34E-06	27929053-28464760	*qSV-3-1/qSD3-3/qSD*	Zhang *et al.*, 2005; Zhou *et al.*,

	2016	GR_3d	3	SNP-3.28228983	28235930	C/T	1.10E-06		*3.2*	2017; Zhang *et al*., 2020
qSG4	2016	GR_5d	4	SNP-4.13516587	13525874	A/G	2.44E-11	13402800-14165946		
	2016	GR_7d	4	SNP-4.13516587	13525874	A/G	2.11E-12			
	2016	GI	4	SNP-4.13516587	13525874	A/G	1.06E-09			
	2017	GR_3d	4	SNP-4.13516587	13525874	A/G	2.05E-08			
	2017	GR_5d	4	SNP-4.13516587	13525874	A/G	5.22E-08			
	2017	GR_7d	4	SNP-4.13516587	13525874	A/G	2.22E-07			
	2017	GI	4	SNP-4.13516587	13525874	A/G	1.53E-08			
qSG6.1	2016	GI	6	SNP-6.14938334	14939334	C/T	2.37E-08	14026370-16558053	*sdr10*	Marzougui *et al*., 2012
	2016	GR_5d	6	SNP-6.15553592	15554591	G/C	4.77E-08			
	2016	GR_7d	6	SNP-6.16287375	16288374	C/G	1.18E-08			
qSG6.2	2016	GI	6	SNP-6.16909222	16910220	C/T	7.39E-07	16738172-17707880	*sdr10*	Marzougui *et al*., 2012
	2016	GR_5d	6	SNP-6.16973171	16974169	G/A	2.26E-07			

	2016	GR_7d	6	SNP-6.16973171	16974169	G/A	3.02E-07			
	2016	GR_3d	6	SNP-6.17390254	17391252	A/G	1.29E-06			
qSG8.1	2016	GR_5d	8	SNP-8.7671097	7672093	A/C	1.71E-06	7517727-7772093	*qGP-8/ARS8.1*	Wang *et al*., 2010; Magwa *et al*., 2016
	2016	GR_7d	8	SNP-8.7671097	7672093	A/C	2.67E-06			
qSG8.2	2017	GR_3d	8	SNP-8.16357985	16360700	C/T	6.25E-07	16260700-17787604		
	2016	GR_3d	8	SNP-8.16664462	16667177	G/A	5.59E-07			
qSG8.3	2017	GR_3d	8	SNP-8.18614731	18617445	G/T	5.80E-06	18407467-18717445	*qSD8*	Gu *et al*., 2005
qSG8.4	2017	GR_3d	8	SNP-8.28047146	28049861	C/T	2.36E-07	27918488-28413290	*qSV-8-2*	Zhang *et al*., 2005
	2017	GR_5d	8	SNP-8.28310575	28313290	G/T	1.48E-06			
qSG11.1	2016	GR_3d	11	SNP-11.6054913	6059162	A/G	4.79E-07	5932676-6173445	*qSD-11*	Guo *et al*., 2004
	2016	GR_5d	11	SNP-11.6054913	6059162	A/G	4.09E-08			
	2016	GR_7d	11	SNP-11.6054913	6059162	A/G	7.00E-08			
	2016	GI	11	SNP-11.6054913	6059162	A/G	5.54E-08			

	2017	GR_5d	11	SNP-11.6054913	6059162	A/G	5.39E-08			
	2017	GR_7d	11	SNP-11.6054913	6059162	A/G	8.34E-09			
	2017	GI	11	SNP-11.6054913	6059162	A/G	1.17E-06			
qSG11.2	2017	GR_5d	11	SNP-11.10416698	10422154	G/A	1.73E-06	10276271-10522154		
	2017	GR_7d	11	SNP-11.10416698	10422154	G/A	1.10E-06			
qSG11.3	2017	GR_3d	11	SNP-11.17787179	18253321	C/T	2.27E-06	18078203-18870973	*qDOR-11-3/qSdn-11/ qSD11.1*	Cai *et al*., 2000; Wan *et a*l., 2006; Zhang *et al*., 2020
	2017	GR_5d	11	SNP-11.17787179	18253321	C/T	2.71E-07			
	2017	GR_7d	11	SNP-11.18131880	18598072	C/G	2.33E-07			
	2017	GI	11	SNP-11.18131880	18598072	C/G	9.47E-07			
qSG12	2016	GR_7d	12	SNP-12.17323117	17329163	G/A	1.49E-07	17229163-17644680	*q1st* $GC_{12.2}$	Dimaano *et al*., 2020

Table S4. GWAS signals overlapped with the selective sweep regions or improvement regions during rice domestication and breeding.

QTLs	Chr.	Position range (bp)	Overlapped candidate selective-sweep regions (bp)	Overlapped candidate breeding signatures (bp)	Reference
qSG1.1	1	75947-444235		1-180000	Xie *et al*., 2015
qSG2.1	2	3857418-4074381	4044001-4061000		Cui *et al*., 2019
qSG2.2	2	6651338-7110119	6650001-6660000		Cui *et al*., 2019
qSG2.3	2	21004089-21490577	21442138-21542139		Huang *et al*., 2012
qSG3.2	3	27929053-28464760		27550001-28020000	Xie *et al*., 2015
qSG4	4	13402800-14165946	13866001-13876000		Cui *et al*., 2019
qSG6.2	6	16738172-17707880	17591001-17622000		Cui *et al*., 2019
qSG8.2	8	16260700-17787604	16211995-16311995		Huang *et al*., 2012
qSG8.4	8	27918488-28413290	27518000-28406000		Molina *et al*., 2011
qSG11.3	11	18078203-18870973		18560001-18600000	Xie *et al*., 2015
qSG12	12	17229163-17644680	17534001-17561000		Cui *et al*., 2019

Table S5. The list of 947 candidate genes in the 19 significant QTLs.

QTLs	No. of candidate genes	MSU ID.	Annotation
qSG1.1	50	LOC_Os01g01150	RNA recognition motif, putative, expressed
		LOC_Os01g01160	heat shock protein DnaJ, putative, expressed
		LOC_Os01g01170	expressed protein
		LOC_Os01g01180	expressed protein
		LOC_Os01g01190	phosphoesterase family protein, putative, expressed
		LOC_Os01g01280	expressed protein
		LOC_Os01g01290	histone-like transcription factor and archaeal histone, putative, expressed
		LOC_Os01g01295	expressed protein
		LOC_Os01g01302	shikimate kinase, putative, expressed
		LOC_Os01g01307	translocon-associated protein beta domain containing protein, expressed
		LOC_Os01g01340	light-induced protein 1-like, putative, expressed
		LOC_Os01g01350	SNF7 domain containing protein, putative, expressed
		LOC_Os01g01360	peptide transporter PTR2, putative, expressed
		LOC_Os01g01369	3-beta-hydroxysteroid-Delta-isomerase, putative, expressed
		LOC_Os01g01380	expressed protein
		LOC_Os01g01390	expressed protein
		LOC_Os01g01400	expressed protein
		LOC_Os01g01410	TKL_IRAK_C-LEC.1 - TKL_IRAK_C-LEC has homology to C-type lectin receptors, expressed
		LOC_Os01g01420	expressed protein
		LOC_Os01g01430	No apical meristem protein, putative, expressed
		LOC_Os01g01440	expressed protein
		LOC_Os01g01450	stress responsive protein, putative, expressed
		LOC_Os01g01470	no apical meristem protein, putative, expressed
		LOC_Os01g01484	light-mediated development protein DET1, putative, expressed

		LOC_Os01g01500	expressed protein
		LOC_Os01g01510	sas10/Utp3 family protein, expressed
		LOC_Os01g01520	transferase family protein, putative, expressed
		LOC_Os01g01570	kinesin heavy chain, putative, expressed
		LOC_Os01g01580	expressed protein
		LOC_Os01g01600	ethylene-responsive protein related, putative, expressed
		LOC_Os01g01610	IscA-like iron-sulfur assembly protein, mitochondrial precursor, putative, expressed
		LOC_Os01g01620	kinase, pfkB family, putative, expressed
		LOC_Os01g01640	Rer1 protein, putative, expressed
		LOC_Os01g01650	isoflavone reductase homolog IRL, putative, expressed
		LOC_Os01g01660	isoflavone reductase, putative, expressed
		LOC_Os01g01670	expressed protein
		LOC_Os01g01680	expressed protein
		LOC_Os01g01689	phosphatidylinositol 3- and 4-kinase family protein, expressed
		LOC_Os01g01700	anaphase-promoting complex subunit 11, putative, expressed
		LOC_Os01g01710	1-deoxy-D-xylulose 5-phosphate reductoisomerase, chloroplast precursor, putative, expressed
		LOC_Os01g01720	pex14, putative, expressed
		LOC_Os01g01730	serine/arginine repetitive matrix protein 2, putative, expressed
		LOC_Os01g01740	protein kinase domain containing protein, expressed
		LOC_Os01g01760	expressed protein
		LOC_Os01g01770	ZOS1-01 - C2H2 zinc finger protein, expressed
		LOC_Os01g01780	exostosin family protein, putative, expressed
		LOC_Os01g01790	expressed protein
		LOC_Os01g01800	expressed protein
		LOC_Os01g01810	expressed protein
		LOC_Os01g01830	OsPOP1 - Putative Prolyl Oligopeptidase homologue, expressed
qSG1.2	41	LOC_Os01g48210	PAP/25A associated domain containing protein,

	putative, expressed
LOC_Os01g48220	protein of unknown function DUF1421 domain containing protein, expressed
LOC_Os01g48250	OsFBDUF5 - F-box and DUF domain containing protein, expressed
LOC_Os01g48260	expressed protein
LOC_Os01g48270	AAA-type ATPase family protein, putative, expressed
LOC_Os01g48280	ubiquitin-conjugating enzyme, putative, expressed
LOC_Os01g48290	dof zinc finger domain containing protein, putative, expressed
LOC_Os01g48300	ATP synthase protein I related, putative, expressed
LOC_Os01g48310	expressed protein
LOC_Os01g48320	transcription factor like protein, putative, expressed
LOC_Os01g48330	protein kinase domain containing protein, expressed
LOC_Os01g48339	expressed protein
LOC_Os01g48360	expressed protein
LOC_Os01g48370	OsFBT1 - F-box and tubby domain containing protein, expressed
LOC_Os01g48380	PPR repeat domain containing protein, putative, expressed
LOC_Os01g48390	expressed protein
LOC_Os01g48410	ATROPGEF7/ROPGEF7, putative, expressed
LOC_Os01g48420	peroxiredoxin, putative, expressed
LOC_Os01g48430	expressed protein
LOC_Os01g48440	glycosyltransferase family 43 protein, putative, expressed
LOC_Os01g48444	OsIAA5 - Auxin-responsive Aux/IAA gene family member, expressed
LOC_Os01g48446	no apical meristem protein, putative, expressed
LOC_Os01g48470	RUNX1 protein, putative, expressed
LOC_Os01g48500	expressed protein
LOC_Os01g48525	expressed protein
LOC_Os01g48530	expressed protein

		LOC_Os01g48540	glyoxal oxidase-related, putative, expressed
		LOC_Os01g48550	expressed protein
		LOC_Os01g48560	expressed protein
		LOC_Os01g48570	expressed protein
		LOC_Os01g48580	ubiquitin-conjugating enzyme E2, putative, expressed
		LOC_Os01g48590	expressed protein
		LOC_Os01g48600	ubiquitin carboxyl-terminal hydrolase family protein, putative, expressed
		LOC_Os01g48610	expressed protein
		LOC_Os01g48620	expressed protein
		LOC_Os01g48640	expressed protein
		LOC_Os01g48650	expressed protein
		LOC_Os01g48660	expressed protein
		LOC_Os01g48680	two pore calcium channel protein 1, putative, expressed
		LOC_Os01g48690	ribosomal protein rpS20, putative, expressed
		LOC_Os01g48700	transcription factor Dp, putative, expressed
qSG2.1	25	LOC_Os02g07480	transglycosylase SLT domain containing protein, expressed
		LOC_Os02g07490	glyceraldehyde-3-phosphate dehydrogenase, putative, expressed
		LOC_Os02g07495	expressed protein
		LOC_Os02g07550	DEFL1 - Defensin and Defensin-like DEFL family, expressed
		LOC_Os02g07575	expressed protein
		LOC_Os02g07580	expressed protein
		LOC_Os02g07600	DEF3 - Defensin and Defensin-like DEFL family, expressed
		LOC_Os02g07624	gamma-thionin family domain containing protein, expressed
		LOC_Os02g07628	gamma-thionin family domain containing protein, expressed
		LOC_Os02g07630	copper-transporting ATPase, putative, expressed
		LOC_Os02g07640	expressed protein

		LOC_Os02g07650	zinc-binding protein, putative, expressed
		LOC_Os02g07654	expressed protein
		LOC_Os02g07670	URED, putative, expressed
		LOC_Os02g07680	cytochrome P450, putative, expressed
		LOC_Os02g07690	VQ domain containing protein, putative, expressed
		LOC_Os02g07700	expressed protein
		LOC_Os02g07709	expressed protein
		LOC_Os02g07720	cytidylyltransferase domain containing protein, expressed
		LOC_Os02g07730	haloacid dehalogenase-like hydrolase domain-containing protein 3, putative, expressed
		LOC_Os02g07740	RNA binding protein, putative, expressed
		LOC_Os02g07750	cell cycle control protein, putative, expressed
		LOC_Os02g07760	aldehyde dehydrogenase, putative, expressed
		LOC_Os02g07770	Myb transcription factor, putative, expressed
		LOC_Os02g07780	OsSPL4 - SBP-box gene family member, expressed
qSG2.2	47	LOC_Os02g12720	interferon-related developmental regulator family protein, putative, expressed
		LOC_Os02g12730	beta-galactosidase precursor, putative, expressed
		LOC_Os02g12740	GPI-anchor transamidase precursor, putative, expressed
		LOC_Os02g12750	tetraspanin family protein, putative, expressed
		LOC_Os02g12760	integral membrane protein, putative, expressed
		LOC_Os02g12780	cytokinin dehydrogenase precursor, putative, expressed
		LOC_Os02g12790	GATA zinc finger domain containing protein, expressed
		LOC_Os02g12794	elongation factor 1-gamma, putative, expressed
		LOC_Os02g12800	elongation factor 1-gamma, putative, expressed
		LOC_Os02g12810	protein kinase domain containing protein, expressed
		LOC_Os02g12820	helix-loop-helix DNA-binding domain containing protein, expressed
		LOC_Os02g12840	DEAD-box ATP-dependent RNA helicase, putative, expressed
		LOC_Os02g12850	RNA recognition motif containing protein, expressed

LOC_Os02g12860	expressed protein
LOC_Os02g12870	expressed protein
LOC_Os02g12880	calcium-binding EF hand family protein, putative, expressed
LOC_Os02g12890	cytochrome P450, putative, expressed
LOC_Os02g12900	cysteine synthase, putative, expressed
LOC_Os02g12910	receptor-like protein kinase 5 precursor, putative, expressed
LOC_Os02g12930	expressed protein
LOC_Os02g12939	expressed protein
LOC_Os02g12960	expressed protein
LOC_Os02g13020	expressed protein
LOC_Os02g13030	expressed protein
LOC_Os02g13060	expressed protein
LOC_Os02g13080	expressed protein
LOC_Os02g13090	expressed protein
LOC_Os02g13100	protein phosphatase 2C, putative, expressed
LOC_Os02g13110	serine/threonine protein phosphatase 2A 55 kDa regulatory subunit B, putative, expressed
LOC_Os02g13120	expressed protein
LOC_Os02g13130	KH domain-containing protein, putative, expressed
LOC_Os02g13140	xaa-Pro dipeptidase, putative, expressed
LOC_Os02g13150	pollen-specific protein SF21, putative, expressed
LOC_Os02g13160	F-box protein PP2-A15, putative, expressed
LOC_Os02g13170	mitochondrial carrier protein, putative, expressed
LOC_Os02g13180	SKP1, putative, expressed
LOC_Os02g13190	F-box family protein, putative, expressed
LOC_Os02g13200	expressed protein
LOC_Os02g13210	transposable element protein, putative, containing Pfam profile: PF03004, Transposase_24, expressed
LOC_Os02g13220	F-box family protein, putative, expressed
LOC_Os02g13260	F-box family protein, putative, expressed
LOC_Os02g13270	Mpv17 / PMP22 family domain containing protein,

			expressed
		LOC_Os02g13290	phosphoethanolamine/phosphocholine phosphatase, putative, expressed
		LOC_Os02g13300	mitochondrial carrier protein, putative, expressed
		LOC_Os02g13304	expressed protein
		LOC_Os02g13310	homeobox domain containing protein, expressed
		LOC_Os02g13320	expressed protein
qSG2.3	56	LOC_Os02g35010	STE_MEKK_ste11_MAP3K.9 - STE kinases include homologs to sterile 7, sterile 11 and sterile 20 from yeast, expressed
		LOC_Os02g35020	glycosyl transferase, putative, expressed
		LOC_Os02g35039	NAD dependent epimerase/dehydratase family protein, putative, expressed
		LOC_Os02g35060	RNA methyltransferase protein, putative, expressed
		LOC_Os02g35070	SPRY-domain containing protein, putative, expressed
		LOC_Os02g35080	T-complex protein 11, putative, expressed
		LOC_Os02g35090	expressed protein
		LOC_Os02g35100	DDT domain containing protein, putative, expressed
		LOC_Os02g35110	tubulin binding cofactor C, putative, expressed
		LOC_Os02g35130	AIG1, putative, expressed
		LOC_Os02g35140	auxin response factor 7, putative, expressed
		LOC_Os02g35144	zinc finger, C3HC4 type domain containing protein, expressed
		LOC_Os02g35150	splicing factor U2AF, putative, expressed
		LOC_Os02g35160	ligatin, putative, expressed
		LOC_Os02g35170	uncharacterized oxidoreductase yoxD, putative, expressed
		LOC_Os02g35180	OsRR2 type-A response regulator, expressed
		LOC_Os02g35190	chloride channel protein, putative, expressed
		LOC_Os02g35200	VP15, putative, expressed
		LOC_Os02g35210	resistance protein, putative, expressed
		LOC_Os02g35220	expressed protein
		LOC_Os02g35230	expressed protein

LOC_Os02g35240	dehydration-responsive element-binding protein, putative, expressed
LOC_Os02g35260	expressed protein
LOC_Os02g35310	12-oxophytodienoate reductase, putative, expressed
LOC_Os02g35315	expressed protein
LOC_Os02g35320	expressed protein
LOC_Os02g35329	RING-H2 finger protein ATL3F, putative, expressed
LOC_Os02g35365	RING-H2 finger protein ATL3F, putative
LOC_Os02g35347	RING-H2 finger protein ATL3F, putative
LOC_Os02g35440	RING-H2 finger protein ATL4O precursor, putative, expressed
LOC_Os02g35450	DNA repair protein Rad51, putative, expressed
LOC_Os02g35460	ZOS2-10 - C2H2 zinc finger protein, expressed
LOC_Os02g35470	calmodulin binding protein, putative, expressed
LOC_Os02g35480	expressed protein
LOC_Os02g35490	MLO domain containing protein, putative, expressed
LOC_Os02g35500	NAD binding domain of 6-phosphogluconate dehydrogenase containing protein, expressed
LOC_Os02g35530	OsFBK8 - F-box domain and kelch repeat containing protein, expressed
LOC_Os02g35540	expressed protein
LOC_Os02g35560	OsFBX53 - F-box domain containing protein, expressed
LOC_Os02g35590	glutathione S-transferase, putative, expressed
LOC_Os02g35600	PHD finger protein, putative, expressed
LOC_Os02g35610	expressed protein
LOC_Os02g35620	expressed protein
LOC_Os02g35630	AAA family ATPase, putative, expressed
LOC_Os02g35640	SFT2, putative, expressed
LOC_Os02g35650	late embryogenesis abundant protein, putative, expressed
LOC_Os02g35660	PTF1, putative, expressed
LOC_Os02g35690	transcription factor like protein, putative, expressed

		LOC_Os02g35700	nucleoside diphosphate kinase, putative, expressed
		LOC_Os02g35710	expressed protein
		LOC_Os02g35720	signal peptidase complex subunit 2, putative, expressed
		LOC_Os02g35730	expressed protein
		LOC_Os02g35740	expressed protein
		LOC_Os02g35750	pentatricopeptide repeat domain containing protein, putative, expressed
		LOC_Os02g35760	STRUBBELIG-RECEPTOR FAMILY 7 precursor, putative, expressed
		LOC_Os02g35770	homeobox associated leucine zipper, putative, expressed
qSG2.4	39	LOC_Os02g58030	leucine-rich repeat-containing protein 40, putative, expressed
		LOC_Os02g58040	OsFBX75 - F-box domain containing protein, expressed
		LOC_Os02g58060	expressed protein
		LOC_Os02g58070	oxidoreductase, putative, expressed
		LOC_Os02g58080	transporter, major facilitator family, putative, expressed
		LOC_Os02g58090	PSP domain containing protein, expressed
		LOC_Os02g58100	expressed protein
		LOC_Os02g58110	expressed protein
		LOC_Os02g58120	oxidoreductase/ transition metal ion binding protein, putative, expressed
		LOC_Os02g58139	OsSigP1 - Putative Type I Signal Peptidase homologue; employs a putative Ser/His catalytic dyad, expressed
		LOC_Os02g58160	expressed protein
		LOC_Os02g58150	expressed protein
		LOC_Os02g58180	expressed protein
		LOC_Os02g58200	expressed protein
		LOC_Os02g58210	jmjC domain containing protein, expressed
		LOC_Os02g58214	expressed protein
		LOC_Os02g58220	RPA2A - Putative single-stranded DNA binding

			complex subunit 2, expressed
		LOC_Os02g58230	C2 domain containing protein, putative, expressed
		LOC_Os02g58250	expressed protein
		LOC_Os02g58260	metallo-beta-lactamase family protein, putative, expressed
		LOC_Os02g58270	metallo-beta-lactamase family protein, putative, expressed
		LOC_Os02g58280	expressed protein
		LOC_Os02g58290	expressed protein
		LOC_Os02g58300	expressed protein
		LOC_Os02g58310	expressed protein
		LOC_Os02g58330	expressed protein
		LOC_Os02g58340	oligopeptidase, putative, expressed
		LOC_Os02g58350	OsRR3 type-A response regulator, expressed
		LOC_Os02g58360	expressed protein
		LOC_Os02g58370	expressed protein
		LOC_Os02g58380	expressed protein
		LOC_Os02g58390	inactive receptor kinase At2g26730 precursor, putative, expressed
		LOC_Os02g58400	expressed protein
		LOC_Os02g58410	expressed protein
		LOC_Os02g58440	zinc finger C-x8-C-x5-C-x3-H type family protein, expressed
		LOC_Os02g58450	uncharacterized ACR, COG1399 family protein, expressed
		LOC_Os02g58460	beta-catenin-like protein 1, putative, expressed
		LOC_Os02g58470	expressed protein
		LOC_Os02g58480	sucrose synthase, putative, expressed
qSG3.1	40	LOC_Os03g11120	methionyl-tRNA synthetase, putative, expressed
		LOC_Os03g11130	expressed protein
		LOC_Os03g11140	pleckstrin homology domain-containing protein-related taxo, putative, expressed
		LOC_Os03g11150	expressed protein

LOC_Os03g11160	cysteine proteinase inhibitor precursor, putative, expressed
LOC_Os03g11170	cysteine proteinase inhibitor precursor, putative, expressed
LOC_Os03g11180	cysteine proteinase inhibitor 6 precursor, putative, expressed
LOC_Os03g11190	expressed protein
LOC_Os03g11200	pre-mRNA-processing factor 39, putative, expressed
LOC_Os03g11210	DUF538 domain containing protein, putative, expressed
LOC_Os03g11220	keratin, type I cytoskeletal 9, putative, expressed
LOC_Os03g11230	expressed protein
LOC_Os03g11240	nucleotidyltransferase, putative, expressed
LOC_Os03g11250	expressed protein
LOC_Os03g11260	RING zinc finger protein-like, putative, expressed
LOC_Os03g11270	expressed protein
LOC_Os03g11280	expressed protein
LOC_Os03g11290	expressed protein
LOC_Os03g11300	expressed protein
LOC_Os03g11310	PPR repeat domain containing protein, putative, expressed
LOC_Os03g11320	heparanase-like protein precursor, putative, expressed
LOC_Os03g11330	glycosyl transferase 8 domain containing protein, putative, expressed
LOC_Os03g11335	expressed protein
LOC_Os03g11340	leucine-rich repeat resistance protein, putative, expressed
LOC_Os03g11350	UDP-glucoronosyl and UDP-glucosyl transferase, putative, expressed
LOC_Os03g11360	leucine rich repeat containing protein, expressed
LOC_Os03g11370	B3 DNA binding domain containing protein, expressed
LOC_Os03g11380	SNARE domain containing protein, putative, expressed
LOC_Os03g11390	expressed protein

		LOC_Os03g11400	targeting protein-related, putative, expressed
		LOC_Os03g11410	mitochondrial-processing peptidase subunit, mitochondrial precursor, putative, expressed
		LOC_Os03g11420	Os3bglu6 - beta-glucosidase/beta-fucosidase/beta-galactosidase, expressed
		LOC_Os03g11430	expressed protein
		LOC_Os03g11440	protein transport protein Sec61 subunit alpha, putative, expressed
		LOC_Os03g11450	expressed protein
		LOC_Os03g11460	expressed protein
		LOC_Os03g11470	DEAD/DEAH box helicase domain containing protein, expressed
		LOC_Os03g11480	expressed protein
		LOC_Os03g11490	expressed protein
		LOC_Os03g11500	DUF647 domain containing protein, putative, expressed
qSG3.2	64	LOC_Os03g49050	possible lysine decarboxylase domain containing protein, expressed
		LOC_Os03g49070	expressed protein
		LOC_Os03g49126	expressed protein
		LOC_Os03g49132	ZOS3-16 - C2H2 zinc finger protein, expressed
		LOC_Os03g49140	ABC1 protein At2g40090 precursor, putative, expressed
		LOC_Os03g49150	yippee zinc-binding protein, putative, expressed
		LOC_Os03g49160	expressed protein
		LOC_Os03g49170	zinc finger family protein, putative, expressed
		LOC_Os03g49180	alkaline phytoceramidase, putative, expressed
		LOC_Os03g49190	oleosin, putative, expressed
		LOC_Os03g49200	WD domain, G-beta repeat domain containing protein, expressed
		LOC_Os03g49210	BRCA1 C Terminus domain containing protein, expressed

LOC_Os03g49220	adenylosuccinate synthetase, chloroplast precursor, putative, expressed
LOC_Os03g49230	acetyltransferase, GNAT family, putative, expressed
LOC_Os03g49240	expressed protein
LOC_Os03g49250	OsFBO16 - F-box and other domain containing protein, expressed
LOC_Os03g49260	lipoxygenase, putative, expressed
LOC_Os03g49270	THION36 - Plant thionin family protein precursor, expressed
LOC_Os03g49280	THION37 - Plant thionin family protein precursor, putative, expressed
LOC_Os03g49300	THION38 - Plant thionin family protein precursor, putative, expressed
LOC_Os03g49310	THION39 - Plant thionin family protein precursor, expressed
LOC_Os03g49350	lipozygenase protein, putative, expressed
LOC_Os03g49360	expressed protein
LOC_Os03g49380	lipoxygenase, putative, expressed
LOC_Os03g49400	ethylene-insensitive protein, putative, expressed
LOC_Os03g49410	expressed protein
LOC_Os03g49420	HEAT repeat family protein, putative, expressed
LOC_Os03g49430	pre-mRNA-splicing factor, putative, expressed
LOC_Os03g49440	phosphatase, putative, expressed
LOC_Os03g49450	expressed protein
LOC_Os03g49464	tetratricopeptide-like helical, putative, expressed
LOC_Os03g49480	elongation of fatty acids protein 2, putative, expressed
LOC_Os03g49485	expressed protein
LOC_Os03g49490	expressed protein
LOC_Os03g49500	ethylene receptor, putative, expressed
LOC_Os03g49510	phosphatidylinositol-4-phosphate 5-kinase, putative, expressed
LOC_Os03g49520	pinin/SDK/memA protein, putative, expressed
LOC_Os03g49524	anthocyanidin 3-O-glucosyltransferase, putative,

	expressed
LOC_Os03g49550	glucosyltransferase, putative, expressed
LOC_Os03g49560	expressed protein
LOC_Os03g49570	domain of unknown function, DUF250 domain containing protein, expressed
LOC_Os03g49580	eukaryotic peptide chain release factor subunit 1-1, putative, expressed
LOC_Os03g49590	expressed protein
LOC_Os03g49600	Os3bglu7 - beta-glucosidase, exo-beta-glucanse, expressed
LOC_Os03g49610	Os3bglu8 - beta-glucosidase, exo-beta-glucansase, high similarity to Os3bglu7, expressed
LOC_Os03g49620	BRASSINOSTEROID INSENSITIVE 1-associated receptor kinase 1 precursor, putative, expressed
LOC_Os03g49630	expressed protein
LOC_Os03g49640	STE_MEKK_ste11_MAP3K.14 - STE kinases include homologs to sterile 7, sterile 11 and sterile 20 from yeast, expressed
LOC_Os03g49680	expressed protein
LOC_Os03g49690	expressed protein
LOC_Os03g49700	SNARE associated Golgi protein, putative, expressed
LOC_Os03g49710	ribosomal protein S13p/S18e, putative, expressed
LOC_Os03g49720	PAP fibrillin family domain containing protein, expressed
LOC_Os03g49730	zinc finger protein, putative, expressed
LOC_Os03g49740	expressed protein
LOC_Os03g49750	protein kinase family protein, putative, expressed
LOC_Os03g49770	oxysterol-binding protein, putative, expressed
LOC_Os03g49790	pattern formation protein EMB30, putative, expressed
LOC_Os03g49800	phosphatidylinositol-4-phosphate 5-kinase, putative, expressed
LOC_Os03g49820	expressed protein
LOC_Os03g49830	expressed protein

		LOC_Os03g49860	expressed protein
		LOC_Os03g49880	TCP family transcription factor, putative, expressed
		LOC_Os03g49900	zinc finger, C3HC4 type domain containing protein, expressed
qSG4	50	LOC_Os04g23460	expressed protein
		LOC_Os04g23550	basic helix-loop-helix family protein, putative, expressed
		LOC_Os04g23580	xylosyltransferase, putative, expressed
		LOC_Os04g23600	D-mannose binding lectin family protein, expressed
		LOC_Os04g23610	expressed protein
		LOC_Os04g23620	D-mannose binding lectin family protein, expressed
		LOC_Os04g23630	expressed protein
		LOC_Os04g23660	ligA, putative, expressed
		LOC_Os04g23689	expressed protein
		LOC_Os04g23700	lectin protein kinase family protein, putative, expressed
		LOC_Os04g23720	lectin protein kinase family protein, putative, expressed
		LOC_Os04g23760	lectin protein kinase family protein, putative, expressed
		LOC_Os04g23820	methionyl-tRNA synthetase, putative, expressed
		LOC_Os04g23830	exonuclease, putative, expressed
		LOC_Os04g23849	expressed protein
		LOC_Os04g23890	AGC_PVPK_like_kin82y.10 - ACG kinases include homologs to PKA, PKG and PKC, expressed
		LOC_Os04g23910	OsMADS25 - MADS-box family gene with MIKCc type-box, expressed
		LOC_Os04g23940	chalcone synthase, putative, expressed
		LOC_Os04g24020	expressed protein
		LOC_Os04g24110	anthocyanin 3-O-beta-glucosyltransferase, putative, expressed
		LOC_Os04g24120	expressed protein
		LOC_Os04g24130	expressed protein
		LOC_Os04g24140	ribose-5-phosphate isomerase A, putative, expressed
		LOC_Os04g24160	expressed protein
		LOC_Os04g24170	RNA recognition motif containing protein, putative,

			expressed
		LOC_Os04g24180	heat shock protein binding protein, putative, expressed
		LOC_Os04g24190	growth-regulating factor 11, putative, expressed
		LOC_Os04g24200	expressed protein
		LOC_Os04g24220	OsWAK32 - OsWAK receptor-like protein kinase, expressed
		LOC_Os04g24250	expressed protein
		LOC_Os04g24290	wall-associated receptor kinase 3 precursor, putative, expressed
		LOC_Os04g24294	OsWAK35d - OsWAK short gene, expressed
		LOC_Os04g24300	OsWAK35a - OsWAK short gene, expressed
		LOC_Os04g24319	jasmonate-induced protein, putative, expressed
		LOC_Os04g24328	jasmonate-induced protein, putative, expressed
		LOC_Os04g24340	phytase, putative, expressed
		LOC_Os04g24410	kinesin heavy chain isolog, putative, expressed
		LOC_Os04g24414	expressed protein
		LOC_Os04g24430	sucrose synthase, putative, expressed
		LOC_Os04g24460	phytase, putative, expressed
		LOC_Os04g24469	jasmonate-induced protein, putative, expressed
		LOC_Os04g24478	jasmonate-induced protein, putative, expressed
		LOC_Os04g24510	OsWAK36 - OsWAK receptor-like protein kinase, expressed
		LOC_Os04g24520	ribosomal protein L51, putative, expressed
		LOC_Os04g24530	AMP-binding domain containing protein, expressed
		LOC_Os04g24540	expressed protein
		LOC_Os04g24550	ATP-binding region, ATPase-like domain containing protein, expressed
		LOC_Os04g24600	cysteine proteinase 1 precursor, putative, expressed
		LOC_Os04g24610	expressed protein
		LOC_Os04g24670	expressed protein
qSG6.1	126	LOC_Os06g24040	expressed protein
		LOC_Os06g24070	myb-like DNA-binding domain containing protein, expressed

LOC_Os06g24130	expressed protein
LOC_Os06g24180	cytochrome P450 84A1, putative, expressed
LOC_Os06g24190	expressed protein
LOC_Os06g24210	expressed protein
LOC_Os06g24240	expressed protein
LOC_Os06g24250	expressed protein
LOC_Os06g24260	expressed protein
LOC_Os06g24290	SCP-like extracellular protein, expressed
LOC_Os06g24390	expressed protein
LOC_Os06g24404	GDSL-like lipase/acylhydrolase, putative, expressed
LOC_Os06g24430	expressed protein
LOC_Os06g24460	pleiotropic drug resistance protein 2, putative, expressed
LOC_Os06g24470	expressed protein
LOC_Os06g24480	expressed protein
LOC_Os06g24490	expressed protein
LOC_Os06g24500	expressed protein
LOC_Os06g24520	expressed protein
LOC_Os06g24540	expressed protein
LOC_Os06g24550	expressed protein
LOC_Os06g24580	expressed protein
LOC_Os06g24594	exonuclease, putative, expressed
LOC_Os06g24704	acyl-coenzyme A oxidase, putative, expressed
LOC_Os06g24711	expressed protein
LOC_Os06g24730	hydrolase, alpha/beta fold family domain containing protein, expressed
LOC_Os06g24840	SacI homology domain containing protein, expressed
LOC_Os06g24850	OsIAA22 - Auxin-responsive Aux/IAA gene family member, expressed
LOC_Os06g24870	bromodomain domain containing protein, expressed
LOC_Os06g24890	expressed protein
LOC_Os06g24910	expressed protein
LOC_Os06g24920	SAR DNA-binding protein-like, putative, expressed

LOC_Os06g24990	xylanase inhibitor protein 1 precursor, putative, expressed
LOC_Os06g25010	glycosyl hydrolase, putative, expressed
LOC_Os06g25020	expressed protein
LOC_Os06g25090	expressed protein
LOC_Os06g25130	expressed protein
LOC_Os06g25240	expressed protein
LOC_Os06g25250	endoribonuclease Dicer, putative, expressed
LOC_Os06g25294	60S ribosomal protein L18a-1, putative, expressed
LOC_Os06g25420	expressed protein
LOC_Os06g25439	NAD dependent epimerase/dehydratase family protein, putative, expressed
LOC_Os06g25460	expressed protein
LOC_Os06g25470	expressed protein
LOC_Os06g25500	expressed protein
LOC_Os06g25510	expressed protein
LOC_Os06g25540	expressed protein
LOC_Os06g25590	expressed protein
LOC_Os06g25605	expressed protein
LOC_Os06g25670	expressed protein
LOC_Os06g25940	expressed protein
LOC_Os06g25950	expressed protein
LOC_Os06g26000	expressed protein
LOC_Os06g26050	expressed protein
LOC_Os06g26080	expressed protein
LOC_Os06g26110	expressed protein
LOC_Os06g26170	expressed protein
LOC_Os06g26180	expressed protein
LOC_Os06g26234	1,4-alpha-glucan-branching enzyme, putative, expressed
LOC_Os06g26270	prefoldin, putative, expressed
LOC_Os06g26340	CGMC_MAPKCMGC_2.10 - CGMC includes CDA, MAPK, GSK3, and CLKC kinases, expressed

LOC_Os06g27360	expressed protein
LOC_Os06g27370	expressed protein
LOC_Os06g27400	expressed protein
LOC_Os06g27530	expressed protein
LOC_Os06g27560	glycosyltransferase protein, putative, expressed
LOC_Os06g27570	expressed protein
LOC_Os06g27590	expressed protein
LOC_Os06g27610	expressed protein
LOC_Os06g27650	expressed protein
LOC_Os06g27670	expressed protein
LOC_Os06g27760	peptide methionine sulfoxide reductase msrB, putative, expressed
LOC_Os06g27770	isoflavone reductase homolog IRL, putative, expressed
LOC_Os06g27790	pentatricopeptide, putative, expressed
LOC_Os06g27800	tm-1GCR237 protein, putative, expressed
LOC_Os06g27820	expressed protein
LOC_Os06g27830	expressed protein
LOC_Os06g27850	peroxidase precursor, putative, expressed
LOC_Os06g27860	poor homologous synapsis 1 protein, putative, expressed
LOC_Os06g27870	signal recognition particle receptor subunit beta, putative, expressed
LOC_Os06g27890	STE_MEK_ste7_MAP2K.8 - STE kinases include homologs to sterile 7, sterile 11 and sterile 20 from yeast, expressed
LOC_Os06g27910	oleosin, putative, expressed
LOC_Os06g27920	zinc ion binding protein, putative, expressed
LOC_Os06g27970	FKBP12-interacting protein of 37 kDa, putative, expressed
LOC_Os06g27980	methyltransferase domain containing protein, expressed
LOC_Os06g28000	carboxyl-terminal peptidase, putative, expressed
LOC_Os06g28030	expressed protein
LOC_Os06g28050	expressed protein

LOC_Os06g28060	ATP-binding region, ATPase-like domain containing protein, expressed
LOC_Os06g28124	glycosyltransferase, putative, expressed
LOC_Os06g28144	expressed protein
LOC_Os06g28160	expressed protein
LOC_Os06g28170	expressed protein
LOC_Os06g28194	expressed protein
LOC_Os06g28240	expressed protein
LOC_Os06g28250	expressed protein
LOC_Os06g28260	expressed protein
LOC_Os06g28270	glycerol 3-phosphate permease, putative
LOC_Os06g28290	expressed protein
LOC_Os06g28300	zinc knuckle family protein, expressed
LOC_Os06g28420	expressed protein
LOC_Os06g28470	expressed protein
LOC_Os06g28480	polygalacturonase inhibitor 1 precursor, putative, expressed
LOC_Os06g28520	expressed protein
LOC_Os06g28524	expressed protein
LOC_Os06g28550	nmrA-like family domain containing protein, expressed
LOC_Os06g28560	expressed protein
LOC_Os06g28590	bg55, putative, expressed
LOC_Os06g28600	expressed protein
LOC_Os06g28630	expressed protein
LOC_Os06g28670	polygalacturonase, putative, expressed
LOC_Os06g28680	expressed protein
LOC_Os06g28690	expressed protein
LOC_Os06g28720	expressed protein
LOC_Os06g28730	expressed protein
LOC_Os06g28740	expressed protein
LOC_Os06g28770	expressed protein
LOC_Os06g28790	expressed protein
LOC_Os06g28800	expressed protein

		LOC_Os06g28820	cycloartenol synthase, putative, expressed
		LOC_Os06g28840	expressed protein
		LOC_Os06g28890	expressed protein
		LOC_Os06g28950	expressed protein
		LOC_Os06g28960	expressed protein
		LOC_Os06g28970	bolA, putative, expressed
		LOC_Os06g29010	expressed protein
qSG6.2	67	LOC_Os06g29310	ulp1 protease family protein, putative, expressed
		LOC_Os06g29350	myosin, putative, expressed
		LOC_Os06g29360	expressed protein
		LOC_Os06g29370	expressed protein
		LOC_Os06g29380	phospholipid-transporting ATPase, putative, expressed
		LOC_Os06g29390	expressed protein
		LOC_Os06g29400	surp module family protein, putative, expressed
		LOC_Os06g29430	cytidine and deoxycytidylate deaminase zinc-binding region family protein, expressed
		LOC_Os06g29440	expressed protein
		LOC_Os06g29450	expressed protein
		LOC_Os06g29460	expressed protein
		LOC_Os06g29470	peroxidase precursor, putative, expressed
		LOC_Os06g29490	expressed protein
		LOC_Os06g29540	expressed protein
		LOC_Os06g29560	expressed protein
		LOC_Os06g29590	expressed protein
		LOC_Os06g29650	CDP-diacylglycerol--inositol 3-phosphatidyltransferase 1, putative, expressed
		LOC_Os06g29670	expressed protein
		LOC_Os06g29679	expressed protein
		LOC_Os06g29690	dynein light chain type 1 domain containing protein, expressed
		LOC_Os06g29700	OsFBD11 - F-box and FBD domain containing protein, expressed
		LOC_Os06g29710	OsFBD12 - F-box and FBD domain containing protein,

	expressed
LOC_Os06g29730	RALFL28 - Rapid ALkalinization Factor RALF family protein precursor, expressed
LOC_Os06g29740	OsFBX197 - F-box domain containing protein, expressed
LOC_Os06g29790	phosphate transporter 1, putative, expressed
LOC_Os06g29800	pentatricopeptide, putative, expressed
LOC_Os06g29804	expressed protein
LOC_Os06g29810	lectin protein kinase family protein, putative, expressed
LOC_Os06g29830	expressed protein
LOC_Os06g29844	MATE efflux family protein, putative, expressed
LOC_Os06g29870	far1-like, putative
LOC_Os06g29880	expressed protein
LOC_Os06g29900	integral membrane protein, putative, expressed
LOC_Os06g29940	expressed protein
LOC_Os06g29950	MATE domain containing protein, expressed
LOC_Os06g29970	expressed protein
LOC_Os06g29994	MATE efflux family protein, putative, expressed
LOC_Os06g30020	PSP domain containing protein, expressed
LOC_Os06g30030	DUF617 domain containing protein, expressed
LOC_Os06g30060	expressed protein
LOC_Os06g30090	helix-loop-helix DNA-binding domain containing protein, expressed
LOC_Os06g30130	cysteine-rich receptor-like protein kinase 10 precursor, putative, expressed
LOC_Os06g30179	cytochrome P450, putative, expressed
LOC_Os06g30210	expressed protein
LOC_Os06g30230	expressed protein
LOC_Os06g30250	expressed protein
LOC_Os06g30280	expressed protein
LOC_Os06g30310	alpha-glucan water dikinase, chloroplast precursor, putative, expressed
LOC_Os06g30320	NOC3 - Putative nucleolar complex subunit 3,

			expressed
		LOC_Os06g30330	U-box domain containing protein, putative, expressed
		LOC_Os06g30370	osMFT1 MFT-Like1 homologous to Mother of FT and TFL1 gene; contains Pfam profile PF01161: Phosphatidylethanolamine-binding protein, expressed
		LOC_Os06g30380	GTP binding protein, putative, expressed
		LOC_Os06g30390	oxidoreductase/ transition metal ion binding protein, putative, expressed
		LOC_Os06g30400	expressed protein
		LOC_Os06g30420	PPR repeat domain containing protein, putative, expressed
		LOC_Os06g30430	disease resistance protein RPM1, putative, expressed
		LOC_Os06g30440	OsGH3.7 - Probable indole-3-acetic acid-amido synthetase, expressed
		LOC_Os06g30450	expressed protein
		LOC_Os06g30460	2-oxo acid dehydrogenases acyltransferase domain containing protein, expressed
		LOC_Os06g30470	expressed protein
		LOC_Os06g30480	expressed protein
		LOC_Os06g30500	cytochrome P450, putative, expressed
		LOC_Os06g30530	expressed protein
		LOC_Os06g30540	expressed protein
		LOC_Os06g30550	expressed protein
		LOC_Os06g30590	expressed protein
		LOC_Os06g30600	expressed protein
qSG8.1	21	LOC_Os08g12740	disease resistance protein RGA3, putative, expressed
		LOC_Os08g12750	serine/threonine-protein kinase HT1, putative, expressed
		LOC_Os08g12760	YT521-B, putative, expressed
		LOC_Os08g12780	chloroplast envelope membrane protein, putative, expressed
		LOC_Os08g12790	cytokinin inducible protein, putative, expressed
		LOC_Os08g12800	glucan endo-1,3-beta-glucosidase precursor, putative,

			expressed
		LOC_Os08g12820	proteasome/cyclosome repeat containing protein, expressed
		LOC_Os08g12830	cytidylyltransferase domain containing protein, expressed
		LOC_Os08g12840	FabA-like domain containing protein, expressed
		LOC_Os08g12850	pentatricopeptide, putative, expressed
		LOC_Os08g12890	DNA binding protein, putative, expressed
		LOC_Os08g12960	speckle-type POZ protein-like, putative, expressed
		LOC_Os08g12970	expressed protein
		LOC_Os08g12980	expressed protein
		LOC_Os08g13000	MBTB20 - Bric-a-Brac, Tramtrack, Broad Complex BTB domain with Meprin and TRAF Homology MATH domain, expressed
		LOC_Os08g13010	expressed protein
		LOC_Os08g13014	expressed protein
		LOC_Os08g13020	BTB and MATH domain containing protein, putative, expressed
		LOC_Os08g13030	MBTB21 - Bric-a-Brac, Tramtrack, Broad Complex BTB domain with Meprin and TRAF Homology MATH domain, expressed
		LOC_Os08g13060	MBTB22 - Bric-a-Brac, Tramtrack, Broad Complex BTB domain with Meprin and TRAF Homology MATH domain, expressed
		LOC_Os08g13070	MBTB23 - Bric-a-Brac, Tramtrack, Broad Complex BTB domain with Meprin and TRAF Homology MATH domain, expressed
qSG8.2	118	LOC_Os08g26810	expressed protein
		LOC_Os08g26820	plant protein of unknown function domain containing protein, expressed
		LOC_Os08g26830	expressed protein
		LOC_Os08g26840	plant protein of unknown function domain containing protein, expressed
		LOC_Os08g26850	plant protein of unknown function domain containing

	protein, expressed
LOC_Os08g26860	expressed protein
LOC_Os08g26870	wound responsive protein, putative, expressed
LOC_Os08g26875	expressed protein
LOC_Os08g26880	bZIP transcription factor domain containing protein, expressed
LOC_Os08g26890	expressed protein
LOC_Os08g26940	expressed protein
LOC_Os08g26950	expressed protein
LOC_Os08g26980	expressed protein
LOC_Os08g26990	response regulator receiver domain containing protein, expressed
LOC_Os08g27010	APE1, putative, expressed
LOC_Os08g27020	expressed protein
LOC_Os08g27030	lipid phosphatase protein, putative, expressed
LOC_Os08g27040	lipid phosphatase protein, putative, expressed
LOC_Os08g27050	expressed protein
LOC_Os08g27060	expressed protein
LOC_Os08g27070	co-chaperone protein SBA1, putative, expressed
LOC_Os08g27080	expressed protein
LOC_Os08g27090	ribosomal protein S17, putative, expressed
LOC_Os08g27100	expressed protein
LOC_Os08g27140	expressed protein
LOC_Os08g27150	chloroplastic group IIA intron splicing facilitator CRS1, chloroplast precursor, putative, expressed
LOC_Os08g27160	expressed protein
LOC_Os08g27170	calmodulin binding protein, putative, expressed
LOC_Os08g27190	OsFBX288 - F-box domain containing protein, expressed
LOC_Os08g27200	expressed protein
LOC_Os08g27210	LTPL3 - Protease inhibitor/seed storage/LTP family protein precursor, putative, expressed
LOC_Os08g27220	AP2 domain containing protein, expressed

LOC_Os08g27230	expressed protein
LOC_Os08g27240	ARID/BRIGHT DNA-binding domain containing protein, expressed
LOC_Os08g27250	expressed protein
LOC_Os08g27290	hAT dimerisation domain-containing protein, putative, expressed
LOC_Os08g27330	expressed protein
LOC_Os08g27370	expressed protein
LOC_Os08g27440	expressed protein
LOC_Os08g27490	expressed protein
LOC_Os08g27540	expressed protein
LOC_Os08g27580	expressed protein
LOC_Os08g27600	expressed protein
LOC_Os08g27640	expressed protein
LOC_Os08g27660	expressed protein
LOC_Os08g27674	LTPL130 - Protease inhibitor/seed storage/LTP family protein precursor, putative, expressed
LOC_Os08g27676	expressed protein
LOC_Os08g27678	protease inhibitor/seed storage/LTP family, putative, expressed
LOC_Os08g27690	expressed protein
LOC_Os08g27700	expressed protein
LOC_Os08g27710	expressed protein
LOC_Os08g27720	pirin, putative, expressed
LOC_Os08g27759	expressed protein
LOC_Os08g27780	OsWAK77 - OsWAK receptor-like cytoplasmic kinase OsWAK-RLCK, expressed
LOC_Os08g27810	OsWAK115 - OsWAK receptor-like protein OsWAK-RLP, expressed
LOC_Os08g27824	NOL1/NOP2/sun family protein, putative, expressed
LOC_Os08g27840	phosphoenolpyruvate carboxylase, putative, expressed
LOC_Os08g27850	endothelial differentiation-related factor 1, putative, expressed

LOC_Os08g27860	EARLY flowering protein, putative, expressed
LOC_Os08g27870	EARLY flowering protein, putative, expressed
LOC_Os08g27880	expressed protein
LOC_Os08g27950	expressed protein
LOC_Os08g27970	expressed protein
LOC_Os08g28000	expressed protein
LOC_Os08g28010	expressed protein
LOC_Os08g28080	coatomer subunit delta, putative, expressed
LOC_Os08g28120	expressed protein
LOC_Os08g28170	nucleobase-ascorbate transporter, putative, expressed
LOC_Os08g28180	PPR repeat domain containing protein, putative, expressed
LOC_Os08g28190	actin, putative, expressed
LOC_Os08g28200	expressed protein
LOC_Os08g28214	tesmin/TSO1-like CXC domain containing protein, expressed
LOC_Os08g28230	IscA-like iron-sulfur assembly protein, mitochondrial precursor, putative, expressed
LOC_Os08g28240	carotenoid cleavage dioxygenase, putative, expressed
LOC_Os08g28250	expressed protein
LOC_Os08g28300	expressed protein
LOC_Os08g28370	expressed protein
LOC_Os08g28400	expressed protein
LOC_Os08g28410	retinal pigment epithelial membrane protein, expressed
LOC_Os08g28460	NBS-LRR disease resistance protein, putative, expressed
LOC_Os08g28470	disease resistance protein RPM1, putative, expressed
LOC_Os08g28480	expressed protein
LOC_Os08g28510	expressed protein
LOC_Os08g28540	resistance protein LR10, putative, expressed
LOC_Os08g28550	expressed protein
LOC_Os08g28560	expressed protein
LOC_Os08g28570	resistance protein, putative, expressed

LOC_Os08g28600	resistance protein, putative, expressed
LOC_Os08g28660	expressed protein
LOC_Os08g28670	pathogenesis-related Bet v I family protein, putative, expressed
LOC_Os08g28680	ubiquitin-conjugating enzyme, putative, expressed
LOC_Os08g28690	expressed protein
LOC_Os08g28700	dnaJ domain containing protein, expressed
LOC_Os08g28710	receptor protein kinase CRINKLY4 precursor, putative, expressed
LOC_Os08g28730	NAD dependent epimerase/dehydratase family protein, putative, expressed
LOC_Os08g28740	expressed protein
LOC_Os08g28780	SKP1, putative, expressed
LOC_Os08g28784	expressed protein
LOC_Os08g28790	dirigent, putative, expressed
LOC_Os08g28800	Skp1 family, dimerisation domain containing protein, expressed
LOC_Os08g28810	expressed protein
LOC_Os08g28820	Skp1 family, dimerisation domain containing protein, expressed
LOC_Os08g28830	pentatricopeptide, putative, expressed
LOC_Os08g28840	hydrolase, NUDIX family, domain containing protein, expressed
LOC_Os08g28850	expressed protein
LOC_Os08g28860	hydrolase, NUDIX family, domain containing protein, expressed
LOC_Os08g28870	receptor-like protein kinase 5 precursor, putative, expressed
LOC_Os08g28880	patatin, putative, expressed
LOC_Os08g28890	protein kinase family protein, putative, expressed
LOC_Os08g28900	OsRR8 type-A response regulator, expressed
LOC_Os08g28940	OsFBX289 - F-box domain containing protein, expressed

		LOC_Os08g28950	response regulator receiver domain containing protein, expressed
		LOC_Os08g28960	expressed protein
		LOC_Os08g28980	exonuclease, putative, expressed
		LOC_Os08g28970	expressed protein
		LOC_Os08g29020	wall-associated kinase-like 2, putative, expressed
		LOC_Os08g29030	expressed protein
		LOC_Os08g29040	protein kinase domain containing protein, expressed
qSG8.3	18	LOC_Os08g29970	para-hydroxybenzoate--polyprenyltransferase, mitochondrial precursor, putative
		LOC_Os08g30014	expressed protein
		LOC_Os08g30020	membrane protein, putative, expressed
		LOC_Os08g30060	proton pump interactor, putative, expressed
		LOC_Os08g30070	expressed protein
		LOC_Os08g30080	1-aminocyclopropane-1-carboxylate oxidase homolog 4, putative, expressed
		LOC_Os08g30090	expressed protein
		LOC_Os08g30095	expressed protein
		LOC_Os08g30100	1-aminocyclopropane-1-carboxylate oxidase homolog 1, putative, expressed
		LOC_Os08g30140	expressed protein
		LOC_Os08g30150	1-aminocyclopropane-1-carboxylate oxidase homolog 1, putative, expressed
		LOC_Os08g30170	expressed protein
		LOC_Os08g30210	1-aminocyclopropane-1-carboxylate oxidase homolog 1, putative, expressed
		LOC_Os08g30240	1-aminocyclopropane-1-carboxylate oxidase homolog 1, putative, expressed
		LOC_Os08g30340	PAS2, putative, expressed
		LOC_Os08g30370	expressed protein
		LOC_Os08g30380	expressed protein
		LOC_Os08g30410	expressed protein
qSG8.4	72	LOC_Os08g44360	male sterility protein 2, putative, expressed

LOC_Os08g44370	inosine-uridine preferring nucleoside hydrolase family protein, putative, expressed
LOC_Os08g44380	L1P family of ribosomal proteins domain containing protein, expressed
LOC_Os08g44390	EF hand family protein, putative, expressed
LOC_Os08g44400	glutathione S-transferase, N-terminal domain containing protein, expressed
LOC_Os08g44410	harpin-induced protein 1 domain containing protein, expressed
LOC_Os08g44420	kinesin-related protein, putative, expressed
LOC_Os08g44430	vesicle-associated membrane protein 727, putative, expressed
LOC_Os08g44440	multiple myeloma tumor-associated protein 2, putative, expressed
LOC_Os08g44450	L1P family of ribosomal proteins domain containing protein, expressed
LOC_Os08g44460	expressed protein
LOC_Os08g44470	expressed protein
LOC_Os08g44480	40S ribosomal protein S25, putative, expressed
LOC_Os08g44510	UDP-N-acetylglucosamine--peptide N-acetylglucosaminyltransferase SPINDLY, putative, expressed
LOC_Os08g44520	peptidyl-prolyl cis-trans isomerase, putative, expressed
LOC_Os08g44530	dihydroxy-acid dehydratase, putative, expressed
LOC_Os08g44540	expressed protein
LOC_Os08g44550	expressed protein
LOC_Os08g44560	expressed protein
LOC_Os08g44590	gibberellin 20 oxidase 2, putative, expressed
LOC_Os08g44610	expressed protein
LOC_Os08g44620	zinc-binding protein, putative, expressed
LOC_Os08g44640	OsSCP41 - Putative Serine Carboxypeptidase homologue, expressed
LOC_Os08g44650	expressed protein

LOC_Os08g44660	EF hand family protein, putative, expressed
LOC_Os08g44670	calreticulin precursor, putative, expressed
LOC_Os08g44680	photosystem I reaction center subunit II, chloroplast precursor, putative, expressed
LOC_Os08g44740	expressed protein
LOC_Os08g44750	auxin-induced protein 5NG4, putative, expressed
LOC_Os08g44760	domain of unknown function DUF966 domain containing protein, expressed
LOC_Os08g44770	copper/zinc superoxide dismutase, putative, expressed
LOC_Os08g44780	expressed protein
LOC_Os08g44790	expansin precursor, putative, expressed
LOC_Os08g44800	expressed protein
LOC_Os08g44810	lactate/malate dehydrogenase, putative, expressed
LOC_Os08g44820	no apical meristem protein, putative, expressed
LOC_Os08g44830	ZOS8-14 - C2H2 zinc finger protein, expressed
LOC_Os08g44840	transferase family protein, putative, expressed
LOC_Os08g44850	C2 domain containing protein, putative, expressed
LOC_Os08g44860	aminopeptidase, putative, expressed
LOC_Os08g44870	MATE efflux family protein, putative, expressed
LOC_Os08g44880	expressed protein
LOC_Os08g44900	expressed protein
LOC_Os08g44910	DNA binding protein, putative, expressed
LOC_Os08g44930	SNARE domain containing protein, putative, expressed
LOC_Os08g44940	DUF260 domain containing protein, putative, expressed
LOC_Os08g44950	zinc finger, C3HC4 type domain containing protein, expressed
LOC_Os08g44960	AP2 domain containing protein, expressed
LOC_Os08g45000	inorganic phosphate transporter, putative, expressed
LOC_Os08g45010	ABC transporter, ATP-binding protein, putative, expressed
LOC_Os08g45030	multidrug resistance protein, putative, expressed
LOC_Os08g45040	zinc finger protein 593, putative, expressed

		LOC_Os08g45050	expressed protein
		LOC_Os08g45060	protein Kinase-like protein TMKL1 precursor, putative, expressed
		LOC_Os08g45070	expressed protein
		LOC_Os08g45080	expressed protein
		LOC_Os08g45090	expressed protein
		LOC_Os08g45100	expressed protein
		LOC_Os08g45110	AP2 domain containing protein, expressed
		LOC_Os08g45120	expressed protein
		LOC_Os08g45130	histone-lysine N-methyltransferase, putative, expressed
		LOC_Os08g45140	OsGrx_S12 - glutaredoxin subgroup I, expressed
		LOC_Os08g45150	GDSL-like lipase/acylhydrolase, putative, expressed
		LOC_Os08g45160	TENA/THI-4 family protein, putative, expressed
		LOC_Os08g45170	carboxyl-terminal peptidase, putative, expressed
		LOC_Os08g45180	kinase, pfkB family, putative, expressed
		LOC_Os08g45190	PGR5, putative, expressed
		LOC_Os08g45200	expressed protein
		LOC_Os08g45210	Mpv17 / PMP22 family domain containing protein, expressed
		LOC_Os08g45220	expressed protein
		LOC_Os08g45230	ENTH domain containing protein, expressed
		LOC_Os08g45240	RNA recognition motif containing protein, putative, expressed
qSG11.1	24	LOC_Os11g10790	expressed protein
		LOC_Os11g10800	dirigent, putative, expressed
		LOC_Os11g10840	expressed protein
		LOC_Os11g10850	dirigent, putative, expressed
		LOC_Os11g10870	dirigent, putative, expressed
		LOC_Os11g10880	expressed protein
		LOC_Os11g10890	expressed protein
		LOC_Os11g10910	chloroplast nucleoid DNA-binding protein, putative, expressed
		LOC_Os11g10920	carboxyl-terminal proteinase, putative, expressed

		LOC_Os11g10930	expressed protein
		LOC_Os11g10940	expressed protein
		LOC_Os11g10980	pyruvate kinase, putative, expressed
		LOC_Os11g10990	heat shock protein DnaJ, putative, expressed
		LOC_Os11g11000	ELMO/CED-12 family protein, putative, expressed
		LOC_Os11g11020	DAG protein, chloroplast precursor, putative, expressed
		LOC_Os11g11030	expressed protein
		LOC_Os11g11040	expressed protein
		LOC_Os11g11050	initiation factor 2 subunit family domain containing protein, expressed
		LOC_Os11g11060	GHMP kinases ATP-binding protein, putative, expressed
		LOC_Os11g11070	mRNA-capping enzyme, putative, expressed
		LOC_Os11g11090	expressed protein
		LOC_Os11g11100	bZIP transcription factor domain containing protein, expressed
		LOC_Os11g11110	expressed protein
		LOC_Os11g11130	expressed protein
qSG11.2	9	LOC_Os11g18300	expressed protein
		LOC_Os11g18310	cycloartenol synthase, putative
		LOC_Os11g18320	expressed protein
		LOC_Os11g18340	cycloartenol synthase, putative, expressed
		LOC_Os11g18366	cycloartenol synthase, putative, expressed
		LOC_Os11g18382	expressed protein
		LOC_Os11g18470	expressed protein
		LOC_Os11g18570	cytochrome P450, putative, expressed
		LOC_Os11g18630	expressed protein
qSG11.3	53	LOC_Os11g31090	transferase family protein, putative, expressed
		LOC_Os11g31100	gibberellin response modulator protein, putative, expressed
		LOC_Os11g31110	expressed protein
		LOC_Os11g31140	expressed protein
		LOC_Os11g31190	nodulin MtN3 family protein, putative, expressed

LOC_Os11g31250	expressed protein
LOC_Os11g31280	expressed protein
LOC_Os11g31330	no apical meristem protein, putative, expressed
LOC_Os11g31340	no apical meristem protein, putative, expressed
LOC_Os11g31360	no apical meristem protein, putative, expressed
LOC_Os11g31380	no apical meristem protein, putative, expressed
LOC_Os11g31390	expressed protein
LOC_Os11g31400	expressed protein
LOC_Os11g31420	expressed protein
LOC_Os11g31430	expressed protein
LOC_Os11g31440	expressed protein
LOC_Os11g31450	expressed protein
LOC_Os11g31470	expressed protein
LOC_Os11g31480	ATP binding protein, putative, expressed
LOC_Os11g31500	ATP binding protein, putative, expressed
LOC_Os11g31530	BRASSINOSTEROID INSENSITIVE 1-associated receptor kinase 1 precursor, putative, expressed
LOC_Os11g31540	BRASSINOSTEROID INSENSITIVE 1-associated receptor kinase 1 precursor, putative, expressed
LOC_Os11g31550	BRASSINOSTEROID INSENSITIVE 1-associated receptor kinase 1 precursor, putative, expressed
LOC_Os11g31560	BRASSINOSTEROID INSENSITIVE 1-associated receptor kinase 1 precursor, putative, expressed
LOC_Os11g31570	expressed protein
LOC_Os11g31590	armadillo, putative, expressed
LOC_Os11g31610	expressed protein
LOC_Os11g31620	OsFBL55 - F-box domain and LRR containing protein, expressed
LOC_Os11g31630	expressed protein
LOC_Os11g31640	serine palmitoyltransferase 2, putative, expressed
LOC_Os11g31650	expressed protein
LOC_Os11g31660	expressed protein
LOC_Os11g31690	expressed protein

		LOC_Os11g31700	expressed protein
		LOC_Os11g31705	expressed protein
		LOC_Os11g31710	expressed protein
		LOC_Os11g31715	expressed protein
		LOC_Os11g31740	expressed protein
		LOC_Os11g31770	expressed protein
		LOC_Os11g31780	expressed protein
		LOC_Os11g31800	expressed protein
		LOC_Os11g31810	expressed protein
		LOC_Os11g31820	expressed protein
		LOC_Os11g31850	expressed protein
		LOC_Os11g31880	phosphoglycerate mutase, putative, expressed
		LOC_Os11g31890	NLI interacting factor-like phosphatase, putative, expressed
		LOC_Os11g31900	acyl carrier protein, putative, expressed
		LOC_Os11g31930	expressed protein
		LOC_Os11g31940	GDSL-like lipase/acylhydrolase, putative, expressed
		LOC_Os11g31950	expressed protein
		LOC_Os11g31980	OsSCP63 - Putative Serine Carboxypeptidase homologue, expressed
		LOC_Os11g32010	expressed protein
		LOC_Os11g32020	expressed protein
qSG12	27	LOC_Os12g29160	LTPL105 - Protease inhibitor/seed storage/LTP family protein precursor, putative, expressed
		LOC_Os12g29170	expressed protein
		LOC_Os12g29180	expressed protein
		LOC_Os12g29220	nodulin MtN3 family protein, putative, expressed
		LOC_Os12g29240	latency associated nuclear antigen, putative
		LOC_Os12g29280	disease resistance protein RGA3, putative, expressed
		LOC_Os12g29290	disease resistance protein RGA3, putative, expressed
		LOC_Os12g29300	CESA10 - cellulose synthase, expressed
		LOC_Os12g29320	expressed protein
		LOC_Os12g29330	no apical meristem protein, putative, expressed

LOC_Os12g29340	expressed protein
LOC_Os12g29350	ATP binding protein, putative, expressed
LOC_Os12g29370	ZmEBE-1 protein, putative, expressed
LOC_Os12g29390	expressed protein
LOC_Os12g29400	GRAM domain containing protein, expressed
LOC_Os12g29410	expressed protein
LOC_Os12g29430	OsWAK125 - OsWAK receptor-like protein OsWAK-RLP, expressed
LOC_Os12g29434	wall-associated receptor kinase 3 precursor, putative, expressed
LOC_Os12g29480	SAM domain containing protein, putative, expressed
LOC_Os12g29500	expressed protein
LOC_Os12g29510	expressed protein
LOC_Os12g29520	auxin response factor, putative, expressed
LOC_Os12g29540	expressed protein
LOC_Os12g29550	expressed protein
LOC_Os12g29560	DHHC zinc finger domain containing protein, expressed
LOC_Os12g29570	expressed protein
LOC_Os12g29580	AGC_PVPK_like_kin82y.19 - ACG kinases include homologs to PKA, PKG and PKC, expressed

Table S6. The polymorphic sites of *OsPK5* in the high-SNP-density dataset of RDP1.

NO.	SNP ID.	Alleles	Position (bp)
*SNP1	SNP-11.6054913.	A/G	6059162
*SNP2	SNP-11.6055096.	C/T	6059345
*SNP3	SNP-11.6055197.	G/C	6059446
*SNP4	SNP-11.6055216.	A/G	6059465
*SNP5	SNP-11.6055262.	A/G	6059511
SNP6	SNP-11.6056709.	C/T	6060958
*SNP7	SNP-11.6059294.	A/G	6063543
*SNP8	SNP-11.6059626.	T/C	6063875
SNP9	SNP-11.6060148.	G/A	6064397
*SNP10	SNP-11.6060349.	G/A	6064598
SNP11	SNP-11.6060903.	C/T	6065152

* indicates significant SNP.

Table S7. Phenotypic data of the extreme accessions with two different haplotypes of *OsPK5*.

NSFTV ID.	Haplotypes	GR_3d (%)		GR_5d (%)		GR_7d (%)		GI	
		2016	2017	2016	2017	2016	2017	2016	2017
NSFTV102	Hap1	99.33 ± 0.94	96.00 ± 0.00	99.33 ± 0.94	98.00 ± 0.00	99.33 ± 0.94	98.00 ± 0.00	20.11 ± 0.75	18.89 ± 0.69
NSFTV3	Hap1	93.33 ± 1.89	96.00 ± 2.00	98.67 ± 1.89	97.00 ± 1.00	98.67 ± 1.89	98.00 ± 0.00	19.36 ± 0.17	22.60 ± 0.40
NSFTV349	Hap1	92.67 ± 0.94	98.00 ± 0.00	99.33 ± 0.94	100.00 ± 0.00	99.33 ± 0.94	100.00 ± 0.00	22.54 ± 0.27	23.67 ± 0.08
NSFTV235	Hap1	94.00 ± 3.27	97.00 ± 1.00	97.33 ± 2.49	99.00 ± 1.00	97.33 ± 2.49	100.00 ± 0.00	22.77 ± 0.44	23.56 ± 0.31
NSFTV203	Hap1	87.33 ± 5.73	89.00 ± 5.00	100.00 ± 0.00	100.00 ± 0.00	100.00 ± 0.00	100.00 ± 0.00	18.96 ± 0.10	19.93 ± 0.15
NSFTV319	Hap2	0.00 ± 0.00	0.00 ± 0.00	0.00 ± 0.00	3.00 ± 1.00	0.00 ± 0.00	3.00 ± 1.00	0.00 ± 0.00	0.35 ± 0.10
NSFTV321	Hap2	0.00 ± 0.00	0.00 ± 0.00	2.67 ± 0.94	8.00 ± 0.00	2.67 ± 0.94	18.00 ± 2.00	0.33 ± 0.12	1.69 ± 0.19
NSFTV360	Hap2	0.00 ± 0.00	2 ± 2.00	0.67 ± 0.94	14.00 ± 4.00	0.67 ± 0.94	20.00 ± 4.00	0.07 ± 0.09	2.17 ± 0.38
NSFTV318	Hap2	0.00 ± 0.00	0.00 ± 0.00	0.00 ± 0.00	3.00 ± 1.00	0.67 ± 0.94	4.00 ± 2.00	0.06 ± 0.08	0.38 ± 0.18
NSFTV317	Hap2	0.00 ± 0.00	0.00 ± 0.00	0.67 ± 0.94	2.00 ± 0.00	1.33 ± 1.89	4.00 ± 0.00	0.12 ± 0.17	0.39 ± 0.03

Table S8. Verification of the polymorphic sites in the coding region of *OsPK5* between Hap1 and Hap2 by resequencing.

**Haplotypes**	**NSFTV ID.**	**Accession name**	**6059511 (SNP5)**	**6061971***	**6063875 (SNP8)**
			Nonsynonymous variant	Synonymous variant	Nonsynonymous variant
Hapl	NSFTV71	IR 36	A	A	T
Hapl	NSFTV313	BR24	A	A	T
Hapl	NSFTV642	Zhenshan 97B	A	A	T
Hap2	NSFTV152	T 1	G	T	C
Hap2	NSFTV318	DJ 24	G	T	C
Hap2	NSFTV329	Kachilon	G	T	C

* indicates new variant identified by resequencing.

Table S9. The primer pairs used in this study.

MSU ID.	Primer names	Primer sequence (5'–3')	Use
LOC_Os03g50885	*OsActin*-F	AGGAAGGCTGGAAGAGGACC	qRT-PCR
	OsActin-R	CGGGAAATTGTGAGGGACAT	
	18S-F	ATGGTGGTGACGGGTGAC	
	18S-R	CAGACACTAAAGCGCCCGGTA	
LOC_Os05g45590	*OsHXK2*-F	TCAGGTCATCCCATCTTCC	
	OsHXK2-R	GGACTCTACGAACAATTTCTCC	
LOC_Os01g53930	*OsHXK6*-F	TCTATGCTCTCGATCTTGGG	
	OsHXK6-R	TCAGGTGAGGTGGAATGG	
LOC_Os01g53680	*OsPFK1*-F	TTCACGGACTACCTCTCC	
	OsPFK1-R	GCTTGACGGTCGAATAGG	
LOC_Os05g44922	*OsPFK2*-F	GAAGCCAAAGAGAACGATAGG	
	OsPFK2-R	CGAAGATGATGCAGACTGG	
LOC_Os11g05110	*OsPK1*-F	AAGCCTGCTGTTGTTACTCGT	
	OsPK1-R	TCACTCCCGTCAAGTACAGC	
LOC_Os07g08340	*OsPK2*-F	CATGAGGAACTGGAACTTAAA	
	OsPK2-R	GACGAAAACGGCATCTAC	
LOC_Os04g37619	*OsZEP*-F	GGATGCCATTGAGTTTGGTT	
	OsZEP-R	TGGCTGACTGAAGTCTCTCG	
LOC_Os03g44380	*OsNCED3*-F	CCCTCCCAAACCATCCAAACCGA	
	OsNCED3-R	TGTGAGCATATCCTGGCGTCGTGA	
LOC_Os12g42280	*OsNCED5*-F	ACATCCGAGCTCCTCGTCGTGAA	
	OsNCED5-R	TTGGAAGGTGTTTTGGAATGAACCA	
LOC_Os02g47470	*OsCYP707A5*-F	CCTCGCAACCAAGTACAGGT	
	OsCYP707A5-R	ACTCCTGCTCGGTGTTCTTG	
LOC_Os01g64000	*OsABI5*-F	AGCGGTGAACCAGTTTGATT	
	OsABI5-R	ATCTGCCTGTTTCCTCTCCA	
LOC_Os03g63970	*OsGA20ox1*-F	AGGGTGTACCCGGACTTCA	
	OsGA20ox1-R	GTGACGATGATGATTAAGCCAGT	
LOC_Os05g06670	*OsGA2ox1*-F	TACGCCAGCAGCTTCACGG	
	OsGA2ox1-R	TCCATCAGCTCCAGCGACA	
LOC_Os01g55240	*OsGA2ox3-F*	TCGTTGCAGGTTCTGACCA	

	OsGA2ox3-R	CCTGTTGTCTCCAAGCCTT	
LOC_Os05g33730	*OsGID1*-F	GTCGTCCTTGTTCCACTAATC	
	OsGID1-R	CTTGTCTTGACTACACTGACC	
LOC_Os03g49990	*OsSLR1*-F	GTGCAGCAGGAGAACTTCG	
	OsSLR1-R	CGGCGAAGGCGGCGTCG	
	OsPK5-BsF	AATAATGGTCTCAGGCGCATCACTCACCAAGATTGT	CRISPR vector construction
	OsPK5-F0	GCATCACTCACCAAGATTGTGTTTTAGAGCTAGAAATAGC	
	OsPK5-BsR	CCAAACTTAATTGGCAAGACGCTTCTTGGTGCC	
	OsPK5-R0	ATTATTGGTCTCTAAACCCAAACTTAATTGGCAAGA	
	OsU3-FD3	GACAGGCGTCTTCTACTGGTGCTAC	Bacteria PCR Sequencing
	TaU3-RD	CTCACAAATTATCAGCACGCTAGTC	
	TaU3-FD2	TTGACTAGCGTGCTGATAATTTGTG	
	OsPK5-CRISPR-F1	ATGTGGGGATTTGATGCGTG	
	OsPK5-CRISPR-R1	CATGCCGTCAACTTACAGGG	
	OsPK5-CRISPR-F2	CACAATTCAACTGGTGGACCA	
	OsPK5-CRISPR-R2	ACACAAAGAAACAATTCACTATGCA	
